# Peripheral mechanism of a carbonyl hydrosilylation catalysed by an SiNSi iron pincer complex†
†Electronic supplementary information (ESI) available: Experimental procedures and computational details, characterisation, crystallographic and quantum-chemical calculation data as well as NMR spectra. CCDC 1416378. For ESI and crystallographic data in CIF or other electronic format see DOI: 10.1039/c5sc02855h


**DOI:** 10.1039/c5sc02855h

**Published:** 2015-09-14

**Authors:** Toni T. Metsänen, Daniel Gallego, Tibor Szilvási, Matthias Driess, Martin Oestreich

**Affiliations:** a Institut für Chemie , Technische Universität Berlin , Straße des 17. Juni 115 , 10623 Berlin , Germany . Email: matthias.driess@tu-berlin.de ; Email: martin.oestreich@tu-berlin.de; b Department of Inorganic and Analytical Chemistry , Budapest University of Technology and Economics , Szent Gellért tér 4 , 1111 Budapest , Hungary

## Abstract

Combined experimental and theoretical analysis of the carbonyl hydrosilylation catalysed by an iron(0) pincer complex reveals an unprecedented mechanism of action. The iron(0) complex is in fact a precatalyst that is converted into an iron(ii) catalyst through oxidative addition of a hydrosilane. Neither the hydrogen atom nor the silicon atom bound to the iron(ii) centre are subsequently transferred onto the carbonyl acceptor, instead remaining at the sterically inaccessible iron(ii) atom throughout the catalytic cycle. A series of labelling, crossover and competition experiments as well as the use of a silicon-stereogenic hydrosilane as a stereochemical probe suggest that the iron(ii) site is not directly involved in the hydrosilylation. Strikingly, it is the silyl ligand attached to the iron(ii) atom that acts as a Lewis acid for carbonyl activation in this catalysis. The whole catalytic process occurs on the *periphery* of the transition metal. Computation of the new *peripheral* as well as plausible alternative *inner* and *outer* sphere mechanisms support the experimental findings.

## Abstract 2

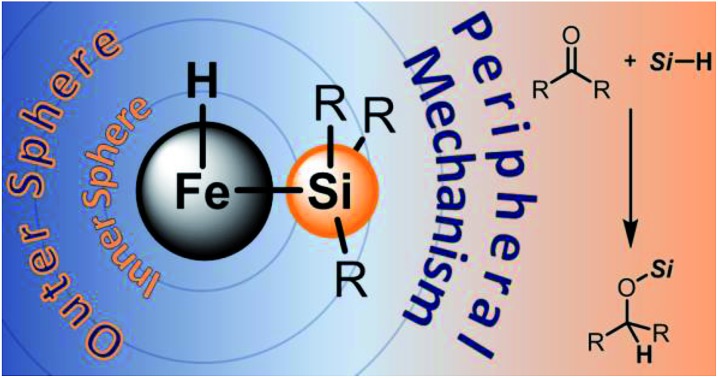
The metal centre remains a spectator through the peripheral mechanism investigated by a combined spectroscopic, crystallographic, and computational analysis.

## Introduction

Iron-catalysed carbonyl hydrosilylation can be traced back to seminal reports by Brunner^1^ but these contributions were decades ahead of their time. With few exceptions, catalyst development was focused on complexes of rare transition metals while limited progress had been made involving abundant transition metals. The pressing demand for sustainable processes finally shifted iron catalysis into the limelight,^2^ and several iron-based catalysts for carbonyl hydrosilylation with different ligand designs were introduced in recent years.^3^ The advent of these new catalysts immediately poses the question whether the mechanisms are similar to those established for rare transition-metal complexes or totally unprecedented.^4^ However, little detail is known about the mechanisms of action of iron complexes in catalytic hydrosilylation.

Mechanisms of transition-metal-catalysed hydrosilylations exhibit a wide variety of modes of activation.^5^ However, the known mechanisms are characterised as either *inner* sphere^6^ where both the substrate and the hydrosilane are directly in contact with the metal or *outer* sphere^7^,^8^ where only one of the two is in contact with the metal centre. The proposed mechanisms for iron-catalysed hydrosilylations range from *inner* sphere mechanisms with σ-bond-metathesis-type Si–H bond cleavage at an iron–oxygen bond3k,3l to *outer* sphere mechanisms with iron acting as a Lewis acid,3*h* either activating the hydrosilane or the carbonyl group.

**Fig. 1:**
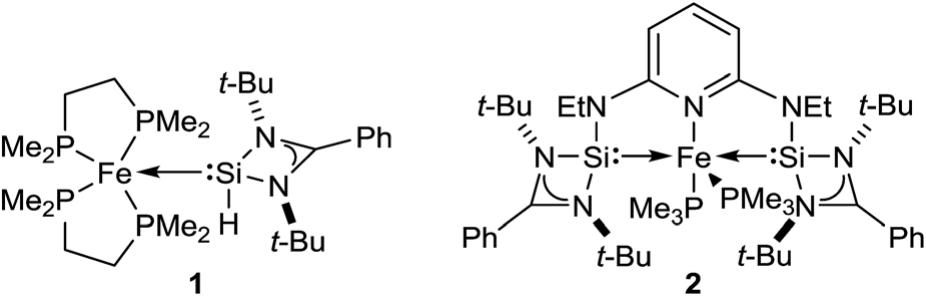
Iron(0) complexes **1** (ref. 9*a*) and **2** (ref. 9*b*) introduced by Driess and co-workers.

Driess and co-workers recently introduced silylenes as σ-donor ligands in iron-based catalysis, and iron(0) complexes **1** and **2** (Fig. 1) were applied to carbonyl hydrosilylation.^9^ Cooperativity between the iron(0) atom and the silicon(ii) hydride in **1** was postulated to be relevant in the catalytic cycle.9*a* The SiNSi iron(0) pincer complex **2** was, in turn, believed to be a precatalyst9b,^10^ but a detailed mechanistic analysis remained challenging. We report here the disclosure of a unique mechanism of a transition-metal-catalysed carbonyl hydrosilylation that takes place neither *inner* nor *outer* sphere but on the *periphery* of the metal centre without its direct involvement.

## Results and discussion

### Optimisation and scope

The SiNSi pincer complex **2** (2.5 mol%) was found to catalyse the hydrosilylation of various acetophenones **3a–3i** with silane **4a** at elevated temperatures^11^ (Table 1, entries 1–9; see the ESI† for the optimisation of the reaction conditions). Both electron-donating (entries 1 and 3) as well as -withdrawing (entry 6) substituents at the aryl group were tolerated with the exception of a Et_2_N group in the *para* position (entry 2). The reaction was, however, sensitive toward steric hindrance. Substituents in the *ortho* position significantly lowered the yield (entries 7–9), and a 2,6-disubstituted substrate did not react (**3j**, entry 10). Benzophenone (**3k**, entry 11) reacted readily while propiophenone and isobutyrophenone (**3l** and **3m**, entries 12 and 13) afforded 18 and 16% yield, respectively. Hydrosilylation of cyclopropyl substituted ketone (**3n**, entry 14) proceeded efficiently^12^ (see the ESI† for full scope).

**Table 1:** Carbonyl hydrosilylation with precatalyst **2**^*a*^

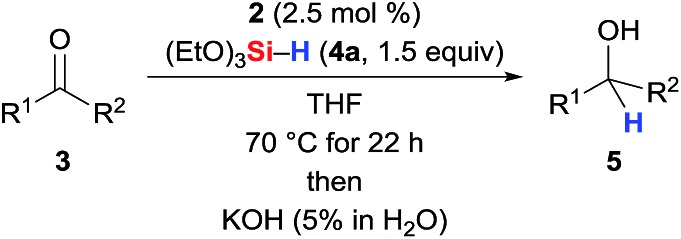					
Entry	**3**	R^1^		R^2^	Yield of **5**^*b*^ (%)
1	**3a**	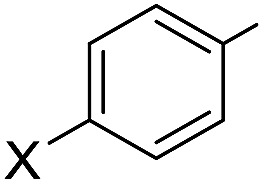	X = MeO	Me	>99 (**5a**)
2	**3b**		X = Et_2_N	Me	40 (**5b**)
3	**3c**		X = Me	Me	82 (**5c**)
4	**3d**		X = Br	Me	>99 (**5d**)
5	**3e**		X = H	Me	93 (**5e**)
6	**3f**		X = CF_3_	Me	95 (**5f**)
7	**3g**	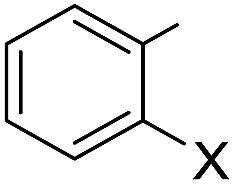	X = MeO	Me	70 (**5g**)
8	**3h**		X = Me	Me	70 (**5h**)
9	**3i**		X = Cl	Me	49 (**5i**)
10	**3j**	Mes		Me	0 (**5j**)
11	**3k**	Ph		Ph	60 (**5k**)
12	**3l**	Ph		Et	18 (**5l**)
13	**3m**	Ph		i-Pr	16 (**5m**)
14	**3n**	c-Pr		Me	>99 (**5n**)

^*a*^Reactions were performed on 0.10 mmol scale employing precatalyst **2** (2.5 mol%) and (EtO)_3_SiH (**4a** 1.5 equiv.).

^*b*^Average yield from two runs determined by GLC-MS analysis and ^1^H NMR spectroscopy using anisole as internal standard.

### Isolation of the active catalyst

**Scheme 1:**
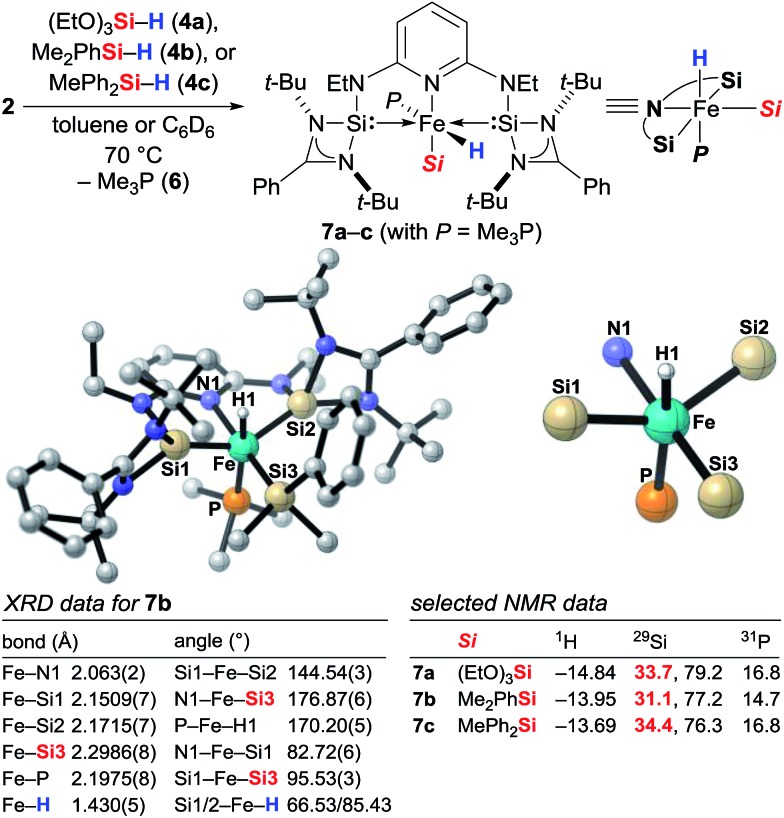
Identification of the catalytically active iron(ii) complexes **7** from iron(0) precatalyst **2** and molecular structure of **7b**. Hydrogen atoms except for the iron hydride are omitted for clarity.

To gain insight into the mechanism, we investigated the reaction between iron(0) complex **2** and hydrosilanes **4a–c** (**2** → **7a–c**, Scheme 1). Heating at 70 °C, a new set of distinct signals appeared in the ^1^H as well as in the ^29^Si and ^31^P NMR spectra. The ^1^H NMR spectrum clearly indicated the formation of an iron hydride, and detailed NMR analysis revealed that the hydride was likely to be *trans* to the apical phosphine ligand. The silyl group was assigned by 2D NMR experiments to be in the equatorial position *trans* to the pyridine ligand. We then obtained single crystals of **7b** (*Si* = Me_2_PhSi) suitable for X-ray diffraction analysis, and that confirmed the molecular structure deduced from the NMR analysis. The structure shows a distorted octahedral iron(ii) coordination environment. The hydride was located tilted toward one of the silylene donor arms deviated from the *trans* coordination to the Me_3_P ligand (P–Fe–H1 170.20(5)°), a situation similar to that of known iron(ii) hydride pincer complexes.3*h* The Fe–Si bond distances 2.1509(7)/2.1715(7) Å for Fe–Si1/Si2 and 2.2986(8) for Fe–Si3 are within the range of iron silylene and silyl complexes.9a,^13^ The *t*-Bu groups encage the iron hydride with a Si1–Fe–Si2 angle far from the linearity, 144.54(3)°.

**Scheme 2:**
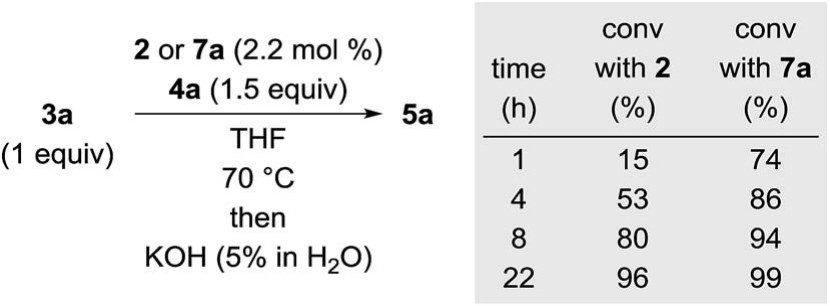
Kinetic profiles of the iron(0) and iron(ii) complexes **2** and **7a** in hydrosilylation.

To validate whether the thus formed iron(ii) complex **7** is the active catalyst, we measured the kinetic profiles for the hydrosilylation of **3a** with hydrosilane **4a** catalysed by **2** or **7a** (Scheme 2). Conversion with **2** was only 15% after 1 h while the reaction had reached 74% with **7a**. The reaction with **7a** continued with significantly higher rate reaching 86% at 4 h compared to only 53% with **2**. After 22 h, nearly full conversion is obtained for both. The greater initial rate of the catalysis with **7a** strongly supports the assignment of the iron(ii) complex **7** as the active catalyst.

### Stoichiometric experiments: hydride transfer

**Scheme 3:**
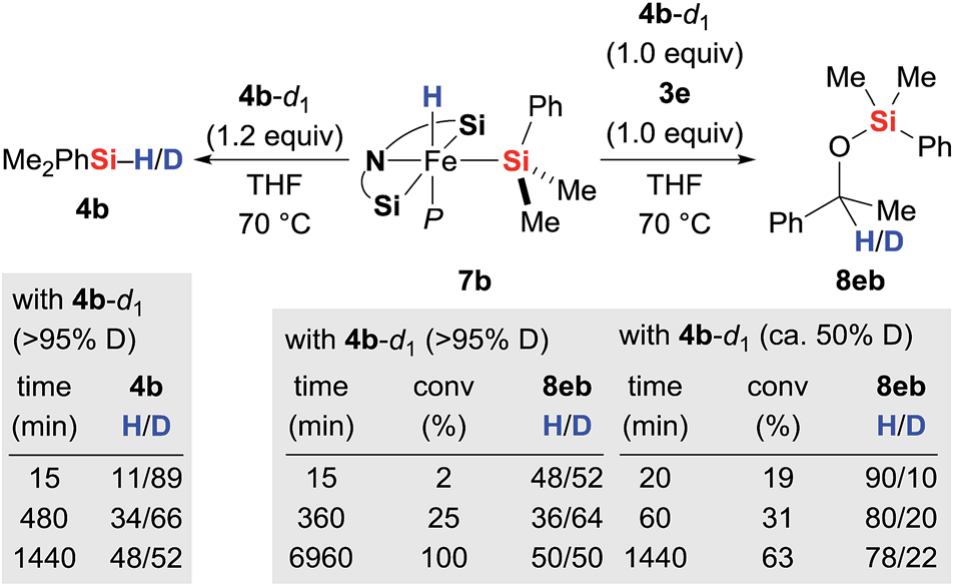
H/D scrambling at the silicon and iron atoms and identification of the hydride source.

With the iron(ii) hydrides **7** in hand, we had a closer look at the hydride transfer. Maintaining **7b** and deuterium-labelled hydrosilane **4b**-*d*_1_ in THF at 70 °C resulted in slow H/D exchange, visible both at the silicon and iron atoms (Scheme 3, left). The stoichiometric reaction between iron(ii) hydride **7b**, hydrosilane **4b**-*d*_1_ (>95% deuteration grade), and ketone **3e** was puzzling though (Scheme 3, right). Initially, the H : D ratio at the methine position of silyl ether **8eb** is nearly 50 : 50. However, it quickly decreases to 36 : 64 at 25% conversion within 6 hours and then gradually increases again, returning to 50 : 50 at full conversion after a few days. Meanwhile, the corresponding reaction with partially deuterated **4b**-*d*_1_ (*ca.* 50% deuteration grade) yielded **8eb** with little deuterium incorporation at 19% conversion (H/D = 90 : 10). That ratio subsequently decreases to 78 : 22 to reach equilibrium after 24 hours. These results reveal that even though the hydride at the silicon atom in **4** is exchanging with the iron-bound hydride in **7**, hydride transfer to the carbonyl carbon atom of **3** most likely occurs from the hydrosilane **4** and not from complex **7**.^14^ Also, the reaction with partially deuterated hydrosilane indicates that the kinetic isotope effect (KIE) of the hydride transfer is significant. Precise value of the KIE could not be measured due to competing H/D exchanges (*vide infra*).

**Scheme 4:**
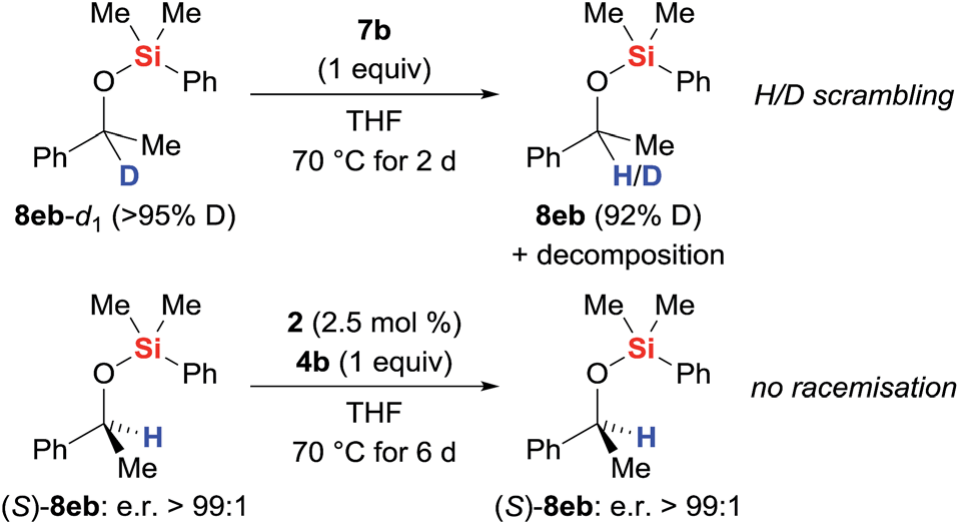
H/D scrambling at the methine carbon atom.

The possible H/D exchange at the methine position of silyl ether **8eb** was verified using **8eb**-*d*_1_ (Scheme 4, top). Treatment of **8eb**-*d*_1_ with equimolar amounts of the iron(ii) hydride **7b** indeed led to H/D scrambling. Conversely, no erosion of the enantiomeric purity was seen when subjecting enantiopure silyl ether (*S*)-**8eb** to the typical protocol (precatalyst **2** and hydrosilane **4b** generate catalyst **7b**, Scheme 4, bottom). The configurational stability of (*S*)-**8eb** suggests that the hydride transfer itself is irreversible, and a concerted mechanism involving frontside attack at the asymmetrically substituted carbon atom is needed to explain the hydrogen atom exchange between the catalyst and the product.

**Scheme 5:**
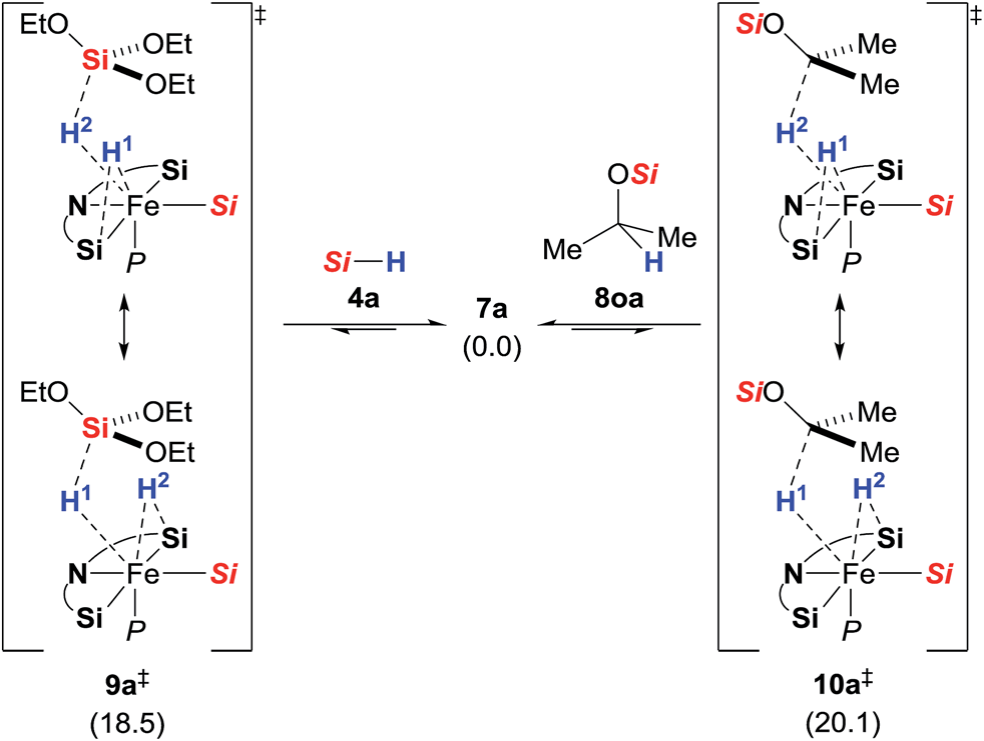
Silylene-assisted H/D scrambling at the hydrosilane silicon atom (**9a^‡^**, left) and the methine carbon atom (**10a^‡^**, right); Gibbs free energies given in parentheses in kcal mol^–1^; *Si* = Si(OEt)_3_.

These unusual scramblings were then investigated by DFT calculations.^15^ Both were found to proceed *via* a silylene-assisted concerted mechanism (**9a^‡^** for Si–H and **10a^‡^** for C–H, Scheme 5) where the hydride on the iron atom is first shifted to the silicon atom of the adjacent donor-stabilised silylene ligand forming a pentacoordinate silicon atom^16^ while the Si–H or C–H bond interact with the now accessible iron centre. Both transition states are paired with their corresponding isomer where the second silylene ligand accepts the hydride. Attempts to locate the transient intermediates between the two degenerate conformations were not successful.

After the transition state, the silylene-bound hydrogen atom migrates back to the silicon and carbon atom, respectively. The activation barriers of the scrambling reactions (18.5 kcal mol^–1^ for Si–H and 20.1 kcal mol^–1^ for C–H) are energetically accessible under the reaction conditions.

### Stoichiometric experiments: phosphine dissociation

**Scheme 6:**
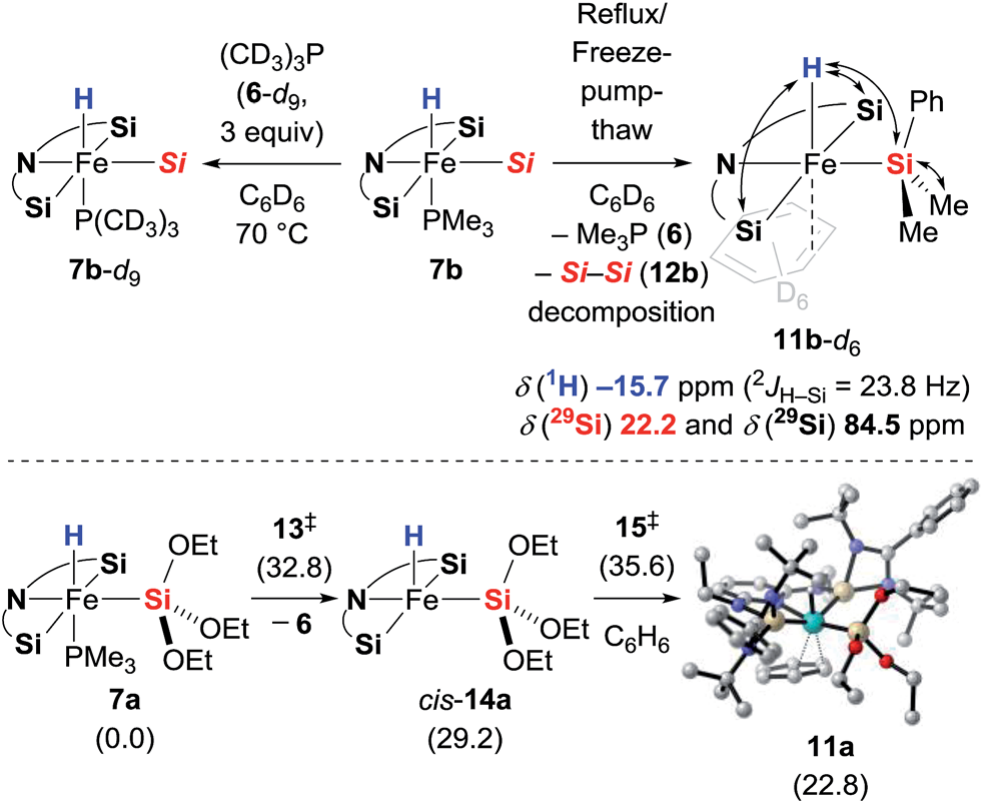
Phosphine scrambling and dissociation. Double-ended arrows in **11b**-*d*_6_ show ^1^H, ^29^Si HMQC NMR correlation. Gibbs free energies given in parentheses in kcal mol^–1^ [*Si* = SiMe_2_Ph].

To probe the potential lability of the phosphine, we performed phosphine crossover experiments. Slow exchange of the phosphine ligand with (CD_3_)_3_P (**6**-*d*_9_) was observed (Scheme 6, top left). When **7b** was subjected to repeated reflux/freeze–pump–thaw cycles, we detected a new iron hydride species that was tentatively assigned as the expected phosphine-dissociated iron silyl hydride **11b**-*d*_6_ with C_6_D_6_ as an η^2^-ligand (Scheme 6, top right). The generation of this new iron compound was accompanied by formation of disilane **12b** and, with longer reaction times, decomposition into a complex mixture. The role of the disilane **12b** in the formation of the complex **11b** remains unexplained. Unfortunately, attempts to isolate **11b**-*d*_6_ were unsuccessful. Its generation in the presence of acetophenone (**3e**) did not lead to the formation of the silyl ether **8eb** (see the ESI† for details), providing further evidence against the role of **11** as an intermediate in the catalytic reaction. In fact, the dissociation of the Me_3_P (**6**) is significantly slower in the presence of ketone **3e**. The formation **11** was also investigated computationally (Scheme 6, bottom). Phosphine dissociation from **7a** gives energetically highly unfavoured intermediate *cis*-**14a**^17^ that readily coordinates benzene to form adduct **11a**.

**Scheme 7:**
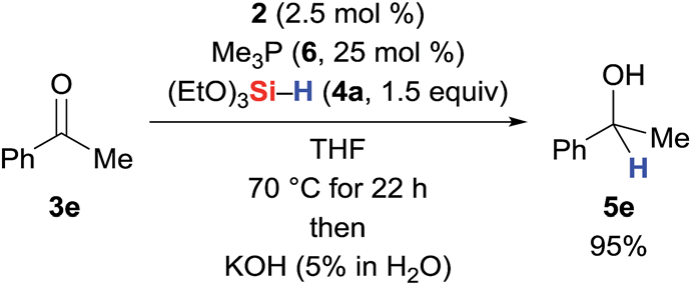
Effect of excess Me_3_P on the catalytic activity.

It must be noted here that catalysis with Guan's related iron(ii) POCOP-pincer complex is thwarted by additional Me_3_P (**6**), indicating dissociation of one of the phosphine ligands as part of the catalytic cycle.3*h* When we added 25 mol% of Me_3_P (**6**, 10 equiv./catalyst) to the reaction mixture, the reaction was unaffected (Scheme 7, *cf.*Table 1, entry 5).

**Scheme 8:**
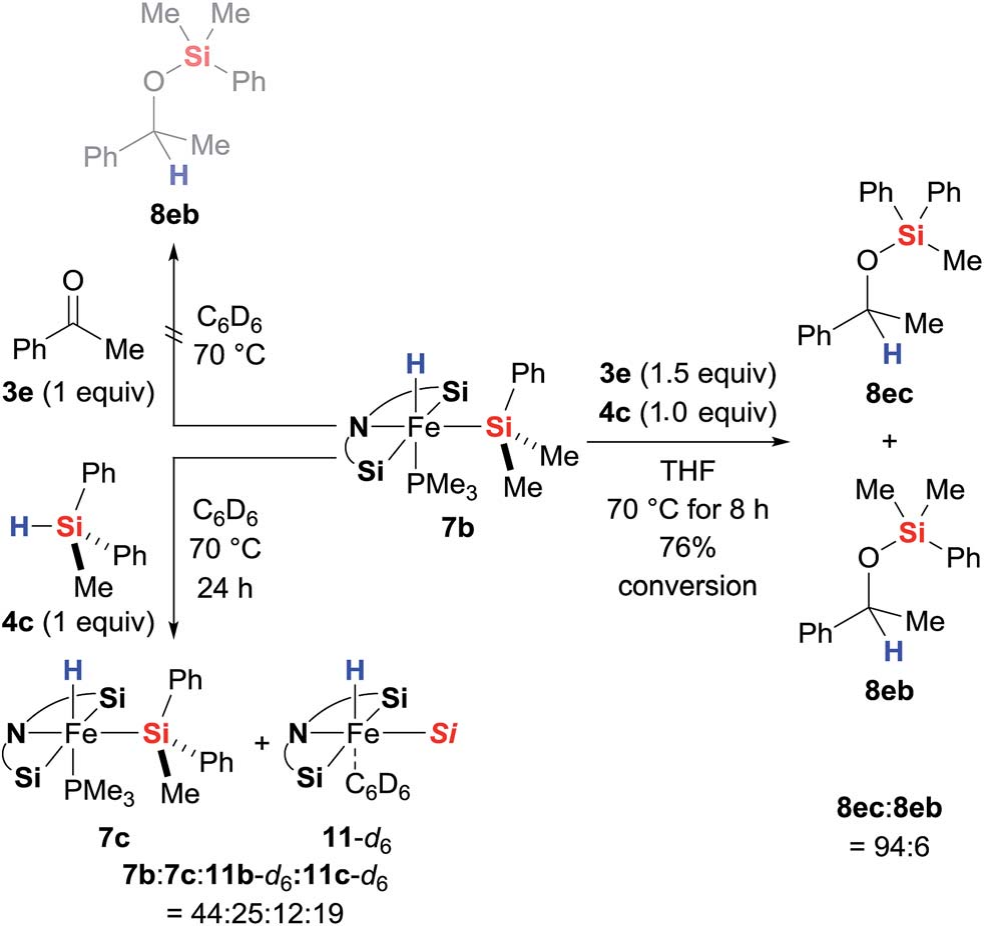
Stoichiometric control and crossover experiments.

As is to be expected from the above observations, the hydride complex **7b** did not produce any silyl ether **8eb** when reacted stoichiometrically with ketone **3e** in the absence of a hydrosilane (Scheme 8, top left). What was fascinating though is that the silyl ligand in **7b** also remains untouched throughout the catalysis: **7b** derived from Me_2_PhSiH (**4b**) catalyses the hydrosilylation of **3e** with MePh_2_SiH (**4c**) with hardly any incorporation of the Me_2_PhSi moiety into the product; silyl ether **8ec** rather than **8eb** is formed almost exclusively (Scheme 8, right). This crossover experiment unequivocally proves that iron(ii) complexes **7** are the actual catalysts, originating from oxidative addition of hydrosilanes **3** to the iron(0) complex **2**; **2** is a precatalyst. During the crossover experiment no changes in the characteristic signals of complex **7b** in the ^1^H and ^31^P NMR spectra were detected. However, when the assumed inability of **7b** and **4c** to exchange their silyl groups was examined with another control experiment (Scheme 8, bottom left), we observed slow exchange with *ca.* 36% conversion of **7b** to **7c** after 24 h. The Me_2_PhSi/MePh_2_Si scrambling was accompanied with formation of phosphine-dissociated, benzene-stabilised compounds **11b** and **11c**. Only traces of Me_2_PhSiH (**4b**) were observed, indicating that the exchange (**7b** to **7c**) is in fact a side product of the decomposition rather than simple scrambling of the silyl groups.

### Hydrosilylation with a silicon-stereogenic hydrosilane

**Scheme 9:**
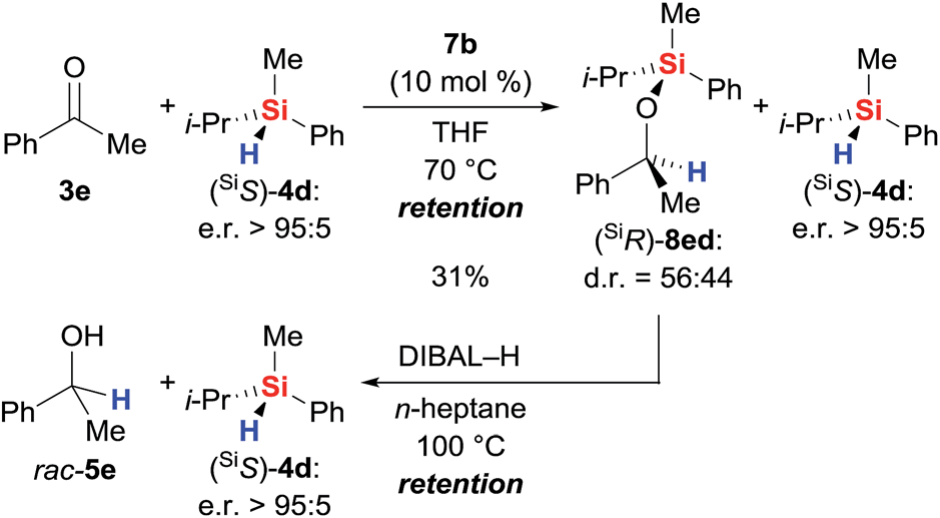
Silicon-stereogenic hydrosilane as a stereochemical probe.

With sufficient knowledge of the active catalyst, we decided to analyse the stereochemical course at the silicon atom of the reacting hydrosilane (Scheme 9).^18^ Catalyst **7b** promoted the reaction between highly enantioenriched hydrosilane (^Si^*S*)-**4d** (e.r. > 95 : 5) and ketone **3e** but conversion was slow as expected from the data obtained with achiral triorganosilane **4b** (see Table S1, entry 12 in the ESI†). After 6 days, we were able to isolate the silyl ether (^Si^*R*)-**8ed** in 31% yield; diastereoselection was poor. The enantiomeric ratio of unreacted (^Si^*S*)-**4d** was found to be unaffected. Subsequent reductive cleavage of the Si–O bond in (^Si^*R*)-**8ed** (known to proceed with stereoretention at silicon atom^19^) liberated (^Si^*S*)-**4d** with overall retention of the stereochemistry at the silicon atom (e.r. > 95 : 5). Hence, the hydrosilylation step involves frontside attack at the silicon atom, and that makes a mechanism involving Lewis-acid activation of the hydrosilane unlikely.^18^

### DFT calculation of conventional *inner* and *outer* sphere mechanisms^15^

**Scheme 10:**
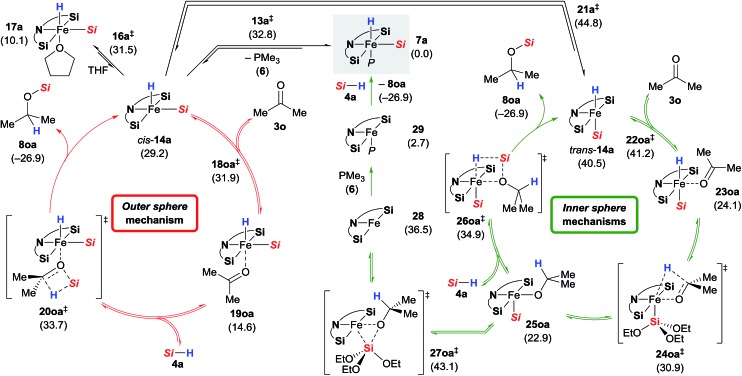
Alternative mechanisms. Gibbs free energies given in parentheses in kcal mol^–1^ [*Si* = Si(OEt)_3_].

Based on the combined findings, we propose that the active iron(ii) catalyst **7** is generated from the iron(0) precatalyst **2** by oxidative addition of hydrosilane **4** to the zero-valent iron atom (Scheme 1). As expected due to the steric congestion around the iron(ii) centre in **7a** (grey box), we did not locate any structure resulting from direct insertion of the ketone C=O group into the iron hydride **7a** or the silylene ligands (not shown). Instead, we were able to find a minimum structure for the phosphine-dissociated complex *cis*-**14a** (Scheme 10, left). In agreement with the experiments, *cis*-**14a** is however significantly higher in energy (29.2 kcal mol^–1^ relative to **7a**). The intermediate *cis*-**14a** readily coordinates THF forming the adduct **17a**. This intermediate is however a resting state if not a “mechanistic dead-end”. Ketone coordination to the iron centre of *cis*-**14a** gives intermediate **19oa** with activated carbonyl group. The catalytic cycle is closed by an *outer* sphere concerted hydrosilane addition **20oa^‡^** to the ketone with an activation barrier of 33.7 kcal mol^–1^.

Isomerisation of *cis*-**14a** to *trans*-**14a** was found to be strongly disfavoured, but again, ketone coordination to *trans*-**14a** lowered the energy significantly (Scheme 10, right). The following hydride transfer from **23oa** passes through **24oa^‡^** (30.9 kcal mol^–1^) to afford the iron alkoxide **25oa**. The silylated alcohol is released by an *inner sphere* silylation through **26oa^‡^** with an energy barrier of 34.9 kcal mol^–1^. An alternative *inner sphere* mechanism could be a reductive elimination from the intermediate **25oa**. However, the energy barrier for the transition state **27oa^‡^** was found to be high, and the resulting iron(0) complex **28** is energetically disfavoured. Recoordination of phosphine **6** gives iron(0) complex **29** which oxidatively adds to a silane **4a** to form **7a**.

### *Peripheral* mechanism: support from DFT calculations

**Scheme 11:**
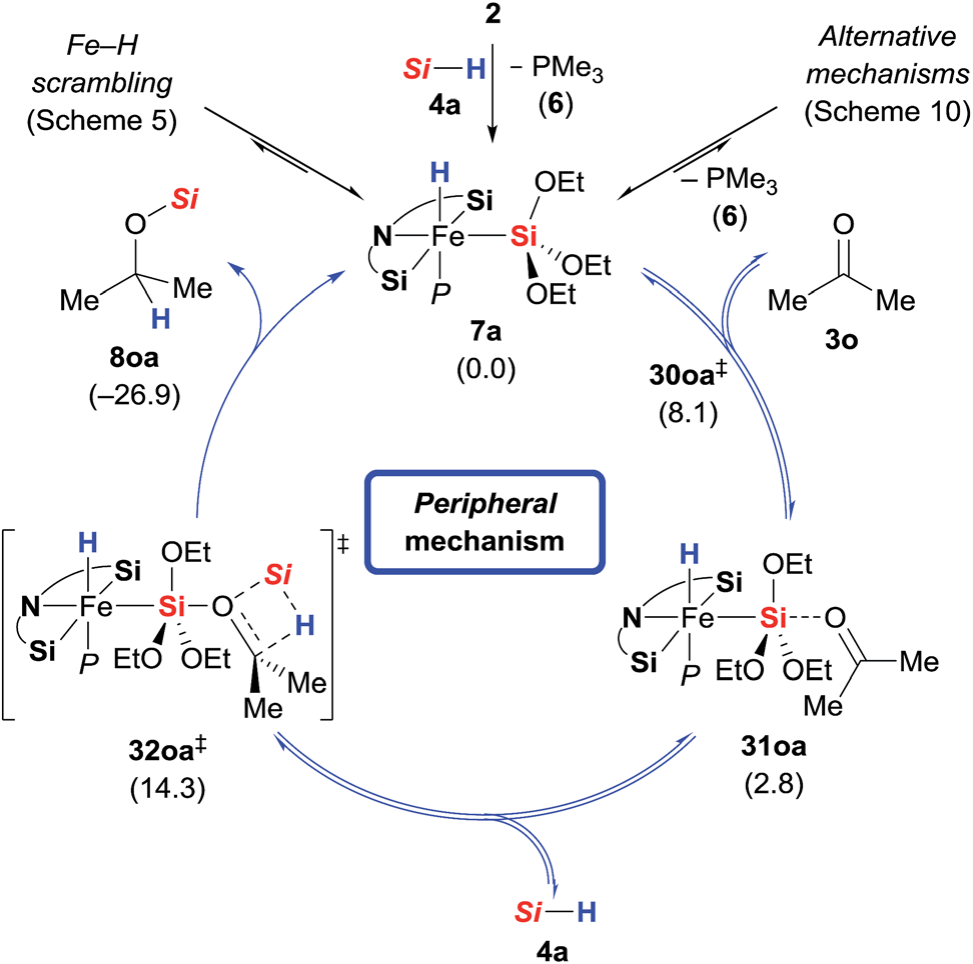
*Peripheral* mechanism. Gibbs free energies given in parentheses in kcal mol^–1^ [*Si* = Si(OEt)_3_].

**Scheme 12:**
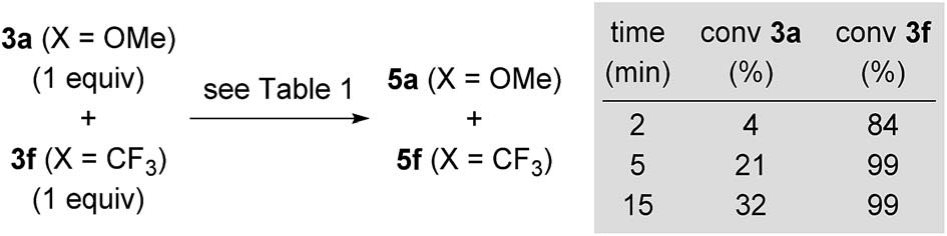
Probing the electronic effect in a competition experiment.

In addition to the high energy barriers, neither *outer* nor *inner sphere* mechanisms give satisfactory fits to the experimental evidence. Hence, we looked for an adduct of **7a** and **3o** wherein the silyl group would act as a Lewis acid (Scheme 11).^8^,^18^,^20^ Coordination of the ketone to the silyl group *via* low energy transition state **30oa^‡^** (8.1 kcal mol^–1^) led to intermediate **31oa** (2.8 kcal mol^–1^) with a pentacoordinate silicon atom, and the C=O double bond being significantly elongated compared to its equilibrium distance from 1.211 to 1.251 Å, indicating activation.^21^ Lewis pair formation is followed by coordination of hydrosilane **4a** to the carbonyl group in **31oa**, and the hydrosilylation event releases **8oa** through transition state **32oa^‡^** with retention at the silicon atom. In accordance with our labelling experiments (*cf.*Scheme 3), this is the rate-determining step (14.3 kcal mol^–1^). To further validate this, we conducted a competition experiment between electron-rich **3a** and electron-deficient **3f** (Scheme 12). The *para* substitution in **3** exerts a pronounced electronic effect, and F_3_C-substituted **3f** was consumed significantly faster than MeO-substituted **3a**. This reactivity pattern is not unprecedented, and it has been seen previously in the activation of carbonyl compounds with silicon-based Lewis acids.^22^ The carbonyl carbon atom in **3f** (X = CF_3_) is more positively polarised accelerating the hydride transfer, than that of donor-substituted **3a** (X = OMe). The reactivity is also in agreement with the proposed KIE based on the control reactions with deuterated silane **4b**-*d*_1_ (Scheme 3).

## Conclusions

The value of the present study is that it demonstrates, to our knowledge for the first time, an unusual case where the transition metal of a catalyst complex is not directly involved in the catalytic process. Activation of both substrate and reagent as well as the bond-forming and -breaking events happen in the ligand sphere, *i.e.*, on the *periphery* of the transition metal. Coincidentally, the mechanism becomes *outer sphere* at silicon.^22^ The more conventional *inner* or *outer* sphere mechanisms do not apply to this unique catalyst.

## Supplementary Material

Supplementary information

Crystal structure data
