# Supplementary material for: Peripheral mechanism of a carbonyl hydrosilylation catalysed by an SiNSi iron pincer complex
Source: Chem Sci. 2015 Sep 14;6(12):7143–9. doi: 10.1039/c5sc02855h (PMC5951209; doi:10.1039/c5sc02855h)
Supplement: Supplementary file 1 [file SC-006-C5SC02855H-s001.pdf]

Electronic Supplementary Material (ESI) for Chemical Science.
This journal is © The Royal Society of Chemistry 2015

Peripheral mechanism of a carbonyl hydrosilylation catalysed by an SiNSi iron pincer complex

Toni T. Metsänen, Daniel Gallego, Tibor Szilvási, Matthias Driess,*
and Martin Oestreich*

Institut für Chemie, Technische Universität Berlin,
Straße des 17. Juni 115, 10623 Berlin, Germany
and
Department of Inorganic and Analytical Chemistry, Budapest University of Technology and Economics, Szent Gellért tér 4, 1111 Budapest, Hungary
martin.oestreich@tu-berlin.de;
matthias.driess@tu-berlin.de

Electronic Supplementary Information

Table of Contents

**1**	**General Information**	**S4**
1.1	Experimental Details	S4
1.2	Single-Crystal X-ray Structure Determinations	S4
1.3	Computational Details	S5
**2**	**General Procedure for the Hydrosilylation of Ketones with Iron(0) Complex 2 (GP 1)**	**S6**
2.1	Optimisation with 4-Methoxyacetophenone (**3a**)	S6
2.2	Substrate Scope	S7
**3**	**Identification of the Active Iron(II) Complex 7**	**S8**
3.1	General Procedure for the Stoichiometric Reaction of Iron(0) Complex **2** with Hydrosilanes **4**	S8
3.1.1	*Si* = $(EtO)_3Si$ (**7a**)	S8
3.1.2	*Si* = Me_2PhSi (**7b**)	S9
3.1.3	*Si* = $MePh_2Si$ (**7c**)	S9
3.2	Crystallographic Data for **7b**	S10
**4**	**Mechanistic Experiments**	**S14**
4.1	Kinetics: Iron(0) complex **2** vs. Iron(II) complex **7a**	S14
4.1.1	Iron(0) complex **2**	S14
4.1.2	Iron(II) complex **7a**	S14

4.2 Scrambling at the Si–H Bond S15
4.2.1 Hydrosilane Si–D/Fe–H Scrambling Using Me_2PhSi–D (**4b**-d_1) S15
4.2.2 Deuteration Studies with Me_2PhSi–D (**4b**-d_1) S16
4.3 Scrambling at the C–H Bond S16
4.3.1 Scrambling Experiment with Deuterated Silyl Ether **8eb**-d_1 S16
4.3.2 Racemisation Experiment with Enantiopure Silyl Ether (*S*)-**8eb** S18
4.4 Phosphine Scrambling S18
4.4.1 Synthesis of Trimethylphosphine-d_9 (**6**-d_9) S18
4.4.2 Scrambling between Complex **7b** and Trimethylphosphine-d_9 (**6**-d_9) S19
4.5 Phosphine Dissociation S19
4.5.1 Phosphine Dissociation in the Presence of Acetophenone (**3e**) S20
4.6 Silyl Scrambling S21
4.7 Silane Cross-over Experiment S22
4.8 Hydrosilylation with Silicon-Stereogenic Hydrosilane S23
4.8.1 Catalytic Reaction between Acetophenone (**3e**) with Enantioenriched Acyclic Hydrosilane (^{Si}S)-**4d** S23
4.8.2 Reductive Si–O Bond Cleavage of Silyl Ether **8ed** S24
4.9 Competition Experiment S24
5 Cartesian Geometries at ωB97X-D/6-31G(d)[Fe:cc-pVTZ] in Ångstrom [Å] S26
5.1 Iron(II) Complex **7a** S26
5.2 Acetone (**3o**) S30
5.3 Triethoxysilane (**4a**) S31
5.4 Silyl Ether **8oa** S32
5.5 Tetrahydrofuran S33
5.6 Benzene S34
5.7 Transition State **9a**‡ S34
5.8 Transition State **10oa**‡ S40
5.9 Transition State **13a**‡ S45
5.10 Intermediate *cis*-**14a** S50
5.11 Transition State **15a**‡ S54
5.12 Intermediate **11a** S58
5.13 Transition State **16a**‡ S63
5.14 Intermediate **17a** S67
5.15 Transition State **18oa**‡ S72
5.16 Intermediate **19oa** S76
5.17 Transition State **20oa**‡ S81
5.18 Transition State **21a**‡ S86

5.19 Intermediate *trans*-**14a** S90

5.20 Transition State **22oa**‡ S94

5.21 Intermediate **23oa** S98

5.22 Transition State **24oa**‡ S102

5.23 Intermediate **25oa** S107

5.24 Transition State **26oa**‡ S111

5.25 Transition State **27oa**‡ S116

5.26 Intermediate **28** S120

5.27 Intermediate **29** S124

5.28 Transition State **30oa**‡ S128

5.29 Intermediate **31oa** S133

5.30 Transition State **32oa**‡ S137

6 NMR Spectra S143

7 Gibbs Free Energy Profile S162

8 References S163

1 General Information

1.1 Experimental Details

All experiments and manipulations were conducted under dry oxygen-free nitrogen using standard Schlenk techniques or in an MBraun glovebox with an atmosphere of purified nitrogen or argon. Solvents were dried by standard methods and freshly distilled prior use. C_6D_6 and toluene-d_8 were dried over sodium and benzophenone, distilled and stored on 4 Å molecular sieves prior to use. Liquid ketones were degassed and stored on 4 Å molecular sieves prior to use. Triethoxysilane **4a**, dimethylphenylsilane **4b**, methyldiphenylsilane **4c**, triethylsilane **4e**, phenylsilane **4f**, and diphenylsilane **4g** were obtained from commercial sources and degassed, distilled, and stored under nitrogen atmosphere. PMHS **4h** was obtained from commercial sources and used without further purification. Iron(0) complex **2**,[S1] enantiomerically enriched hydrosilane (^{Si}*S*)-**4d**,[S2] silyl ether (*S*)-**8eb**,[S3] and tris(pentafluorophenyl)borane[S4] were prepared according to reported procedures. ^{1}H, ^{13}C, ^{29}Si, and ^{31}P NMR spectra were recorded on Bruker AV700, AV500, or AV400 instruments. The NMR chemical shifts are reported in parts per million (ppm) and are referenced to the residual solvent resonance as internal standard (^{1}H, C_6D_5H, 7.15 ppm; toluene-d_8, 2.09 ppm; $CHCl_3$, 7.26 ppm; ^{13}C, C_6D_6, 128.0 ppm; toluene-d_8, 20.4 ppm; $CDCl_3$, 77.16 ppm) or an external standard (^{31}P, 85% H_3PO_4, 0.0 ppm; ^{29}Si, TMS, 0.0 ppm). All signals were unambiguously assigned by a combination of 2D NMR ^{1}H−^{1}H COSY, HSQC, HMBC correlation spectroscopy. Data are reported as follows: chemical shift, multiplicity (br s = broad singlet, s = singlet, d = doublet, t = triplet, q = quartet, sept = septet, m = multiplet), coupling constants (Hz), and integration. Mass spectra were recorded using APCI or ESI as ionization source and a LTQ Orbitrap XL as analyser. IR spectra were recorded on a Perkin-Elmer Spectrum 100 FT-IR. GC−MS measurements were conducted on a Shimadzu GC-2010 gas chromatograph (30 m Rxi-5ms column) linked to a Shimadzu GCMA-QP 2010 Plus mass spectrometer. Enantiomeric excesses were determined by analytical high pressure liquid chromatography (HPLC) analysis on an *Agilent Technologies* 1290 Infinity or an Agilent Technologies 1200 Infinity instrument with a chiral stationary phase using a *Daicel Chiralcel* OJ-RH column (MeCN/H_2O mixtures as solvent), a *Daicel Chiralcel* OJ-H column (*n*-heptane/*i*PrOH mixtures as solvent), or a *Daicel Chiralpak* IB column (*n*-heptane/*i*PrOH mixtures as solvent).

1.2 Single-Crystal X-ray Structure Determinations

The crystal was mounted on a glass capillary in perfluorinated oil and measured in a cold nitrogen flow. The data were collected on an Agilent Technologies SuperNova (single source) at 150 K (Cu K_α radiation, λ= 1.5418 Å). The structure was solved by direct methods

and refined on *F2* with the SHELX-97 software package.[S5] The positions of the hydrogen atoms were calculated and considered isotropically according to a riding model except the hydride on the iron centre which could be defined from the Fourier electron density map.

1.3 Computational Details

Due to the possible importance of zwitterionic and charge transfer resonance structures we intended to choose long-range corrected density functional which can correctly describe the physical interactions in such systems. Dispersion correction seemed necessary to provide realistic description of the problem, because of the large size of the reactants and the catalyst (over 100 atoms all together). Therefore, we chose ωB97X-D density functional[S6] which satisfied these conditions and benchmark calculations showed that it had been able to provide accurate results for reaction energies, kinetics, as well as noncovalent interactions.[S7] For iron atoms, we employed the all-electron triple-ζ cc-pVTZ basis set[S8] which allows flexible description. For other atoms (H, C, N, O, Si, and P), we applied 6-31G(d) basis set.[S9] Frequency calculations (temperature = 343 K) were carried out to evaluate stationary points; minima no imaginary frequency, transition states with one imaginary frequency. All calculations were carried out with GAUSSIAN 09 program package.[S10] The structures were drawn using CYLview 1.0b.

2 General Procedure for the Hydrosilylation of Ketones with Iron(0) Complex 2 (GP 1)

A Schlenk flask equipped with a magnetic stirrer is charged with solution of ketone **3** (0.10 mmol, 1.0 equiv), hydrosilane **4**, and iron(0) complex **2** in the indicated solvent (2 mL). The mixture is maintained at indicated temperature for 22 h and then quenched with 2 mL of a KOH solution (5% in H_2O). The reaction mixture is maintained for further 2 h at room temperature and subsequently extracted with diethyl ether (3 × 5.0 mL). The combined organic layers are dried over Na_2SO_4 and filtered. Anisole (internal standard) is added, and an aliquot is taken for GC-MS analysis.

2.1 Optimisation with 4-Methoxyacetophenone (3a)

According to **GP 1**, 4-methoxyacetophenone (**3a**, 15 mg, 0.10 mmol, 1.0 equiv) was hydrosilylated under various conditions (Table S1).

Table S1. Hydrosilylation of 4-methoxyacetophenone (**3a**) using iron(0) complex **2** as precatalyst.

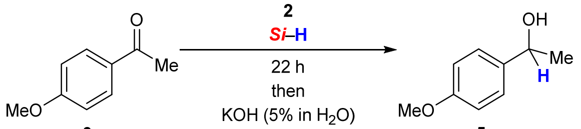

3a **5a**

entry	solvent	temp. (°C)	hydrosilane **4** (equiv)	catalyst loading (mol %)	yield of **5a** (%)
1	hexanes	70	$(EtO)_3SiH$ (3)	2.5	89
2	toluene	70	$(EtO)_3SiH$ (3)	2.5	92
3	THF	70	$(EtO)_3SiH$ (3)	2.5	96
4	dioxane	70	$(EtO)_3SiH$ (3)	2.5	85
5	DMA	70	$(EtO)_3SiH$ (3)	2.5	95
6	THF	40	$(EtO)_3SiH$ (3)	2.5	68
7	THF	50	$(EtO)_3SiH$ (3)	2.5	86
8	THF	60	$(EtO)_3SiH$ (3)	2.5	91
9	THF	80	$(EtO)_3SiH$ (3)	2.5	99
10	THF	40	$PhSiH_3$ (3)	2.5	48
11	THF	40	Ph_2SiH_2 (3)	2.5	40
12	THF	40	Me_2PhSiH (3)	2.5	26
13	THF	40	Et_3SiH (3)	2.5	1
14	THF	40	PMHS (3)	2.5	62
15	THF	70	PMHS (3)	2.5	43
16	THF	70	$(EtO)_3SiH$ (1.1)	2.5	73
17	THF	70	$(EtO)_3SiH$ (1.5)	2.5	>99
18	THF	70	$(EtO)_3SiH$ (2)	2.5	>99
19	THF	70	$(EtO)_3SiH$ (2.5)	2.5	>99
20	THF	70	$(EtO)_3SiH$ (4)	2.5	98
21	THF	70	$(EtO)_3SiH$ (1.5)	0	0
22	THF	70	$(EtO)_3SiH$ (1.5)	0.5	77
23	THF	70	$(EtO)_3SiH$ (1.5)	1.0	88

2.2 Substrate Scope

According to **GP 1**, various ketones **3** (0.10 mmol, 1.0 equiv) were hydrosilylated with triethoxysilane (**4a**, 25 mg, 0.15 mmol, 1.5 equiv), and iron(0) complex **2** (2.2 mg, 2.5 μmol, 2.5 mol %).

Removal of diethyl ether and dissolution in $CDCl_3$ permitted the control also by NMR spectroscopy. The NMR spectra of the corresponding products were in accordance with the reported alcohols in the corresponding references (Table S2).

Table S2. Substrate scope for the hydrosilylation of ketones using iron(0) complex **2** as precatalyst.

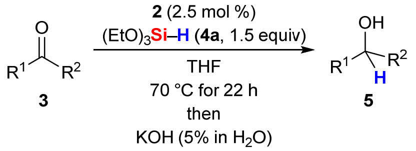

entry	3	R^1	R^2	yield of **5** (%)	ref.
1	3a	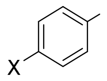 X = OMe	Me	>99 (**5a**)	S1,S11
2	3b	X = Et_2N	Me	40 (**5b**)	S13
3	3c	X = Me	Me	82 (**5c**)	S1,S14
4	3d	X = Br	Me	>99 (**5d**)	S1,S15
5	3e	X = H	Me	93 (**5e**)	S1,S11
6[a]				95 (**5e**)	
7	3f	X = CF_3	Me	95 (**5f**)	S1,S11
8	3g	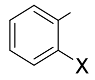 X = OMe	Me	70 (**5g**)	S14
9	3h	X = Me	Me	70 (**5h**)	S16
10	3i	X = Cl	Me	49 (**5i**)	S14
11	3j	Mes	Me	0 (**5j**)	S14
12	3k	Ph	Ph	60 (**5k**)	S16
13	3l	Ph	Et	18 (**5l**)	S14
14	3m	Ph	*i*-Pr	16 (**5m**)	S14
15	3n	*c*-Pr	Me	>99 (**5n**)	S20
16	3p	4-pyridyl	Me	92 (**5p**)	S17
17	3q	2-tolyl	Ph	41 (**5q**)	S14
18	3r	4-Br-C_6H_4	Ph	72 (**5r**)	S18
19	3s	2-furanyl	Me	84 (**5s**)	S19
20	3t	2,6-Me_2-4-*t*-Bu-C_6H_2	Me	3 (**5t**)	S3
21	3u	4-*t*-Bu-cyclohexanone		25 (*cis*-**5u**) (d.r. > 20:1)	S21

[a] In the presence of 25 mol % of PMe_3 (**6**).

3 Identification of the Active Iron(II) Complex 7

3.1 General Procedure for the Stoichiometric Reaction of Iron(0) complex 2 with Hydrosilanes 4

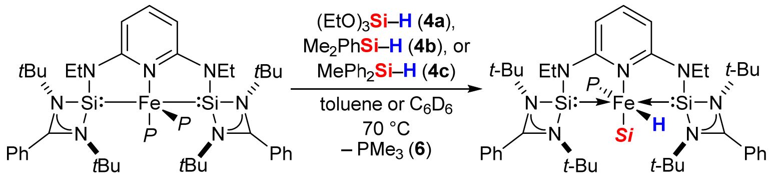

2 **6**

Complex **2** (44.5 mg, 50.0 µmol, 1.00 equiv) was weighed in a Schlenk flask with a magnetic stirrer. The hydrosilane **4** (0.15 mmol, 3 equiv) was weighed in a vial and dissolved in 2.0 mL of toluene (0.5 mL of C_6D_6 for NMR studies) and added into the Schlenk flask. The reaction mixture was heated in an oil bath at 70 °C changing the colour from dark purple to dark red. The time for completion (100% conversion by NMR) varied depending on the hydrosilane used: $(EtO)_3SiH$ 12 h, Me_2PhSiH 6 days, and $MePh_2SiH$ reached 90% conversion after 6 days. The reaction mixture was concentrated, and the product was obtained as a crude red oil. The crude product was dissolved in C_6H_6 and cold sublimation of the solvent *in vacuo* afforded the desired product as a red powder.

3.1.1 *Si* = $(EtO)_3Si$ (7a)

^{1}H NMR (500 MHz, C_6D_6, 298 K): δ(ppm) = –14.83 (d, $^2J_{H\text{-}P}$ = 3.2 Hz, $^2J_{H\text{-}Si}$ = 19.3 Hz, 1H, Fe–H), 1.19 (s, 18H, NC(CH_3)$_3$), 1.41 (t, $^3J_{H\text{-}H}$ = 7.0 Hz, 6H, NCH'H-CH_3), 1.47 (s, 18H, NC(CH_3)$_3$), 1.48 (d, $^2J_{H\text{-}P}$ = 6.2 Hz, 9H, P(CH_3)$_3$), 1.58 (t, $^3J_{H\text{-}H}$ = 7.1 Hz, 9H, SiOCH$_2$CH_3), 3.38 (dq, $^2J_{H\text{-}H}$ = 14.0 Hz, $^3J_{H\text{-}H}$ = 7.0 Hz, 2H, NCH'*H*–CH$_3$), 3.57 (dq, $^2J_{H\text{-}H}$ = 14.0 Hz, $^3J_{H\text{-}H}$ = 7.0 Hz, 2H, NC*H*'H–CH$_3$), 4.33 (q, $^3J_{H\text{-}H}$ = 6.9 Hz, 6H, SiOCH_2CH$_3$), 5.91 (d, $^3J_{H\text{-}H}$ = 8.1 Hz, 2H, 3,5-H py), 6.96-7.08 (m, 6H, C$_{arom.}$*H*), 7.24 (t, $^3J_{H\text{-}H}$ = 8.0 Hz, 1H, 4-H py), 7.23 (d, $^3J_{H\text{-}H}$ = 7.2 Hz, 2H, C$_{arom.}$*H*), 7.72 (d, $^3J_{H\text{-}H}$ = 7.6 Hz, 2H, arom. C*H*). **^{13}C{^{1}H} NMR** (126 MHz, C_6D_6, 298 K): δ(ppm) = 15.3 (NCH$_2$–CH$_3$), 19.8 (SiOCH$_2$CH$_3$), 25.9 (d, $^1J_{C\text{-}P}$ = 18.2 Hz, P(*C*H$_3$)$_3$), 31.9 (NC(*C*H$_3$)$_3$), 32.6 (NC(*C*H$_3$)$_3$), 38.9 (N*C*H$_2$-CH$_3$), 53.7 (N*C*(CH$_3$)$_3$), 54.1 (N*C*(CH$_3$)$_3$), 56.9 (SiO*C*H$_2$CH$_3$), 94.2 (3,5-C$_{arom.}$ py), 127.1(C$_{arom.}$), 128.5 (C$_{arom.}$), 129.0 (C$_{arom.}$), 129.7 (C$_{arom.}$), 131.0 (4-C$_{arom.}$ py), 132.7 (C$_{arom.}$ quaternary Ph), 133.9 (C$_{arom.}$), 168.1 (2,6-C$_{arom.}$ py), 171.7 (N*C*N). **^{29}Si{^{1}H} NMR** (80 MHz, C_6D_6, 298 K): δ(ppm) = 33.7 (d, $^2J_{Si\text{-}P}$ = 58.8 Hz, Si(OEt)$_3$), 79.2 (d, $^2J_{Si\text{-}P}$ = 24.3 Hz, Si:→Fe). **^{31}P{^{1}H} NMR** (202 MHz, C_6D_6, 298 K): δ(ppm) = 16.8.

3.1.2 *Si* = Me_2PhSi (7b)

^{1}H NMR (500 MHz, C_6D_6, 298 K): δ(ppm) = –13.95 (d, $^2J_{H\text{-}P}$ = 1.4 Hz, $^2J_{H\text{-}Si}$ = 19.7 Hz, 1H, Fe–H), 1.06 (s, 18H, NC(CH^A_3)$_3$), 1.08 (s, 6H, Si(CH_3)$_2$Ph), 1.28 (s, 18H, NC(CH^B_3)$_3$), 1.38 (t, $^3J_{H\text{-}H}$ = 7.0 Hz, 6H, NCH'H-CH_3), 1.44 (d, $^2J_{H\text{-}P}$ = 6.3 Hz, 9H, P(CH_3)$_3$), 3.33 (dq, $^2J_{H\text{-}H}$ = 13.2 Hz, $^3J_{H\text{-}H}$ = 6.6 Hz, 2H, NCH'*H*-CH$_3$), 3.57 (dq, $^2J_{H\text{-}H}$ = 13.2 Hz, $^3J_{H\text{-}H}$ = 6.6 Hz, 2H, NC*H'*H-CH$_3$), 5.90 (d, $^3J_{H\text{-}H}$ = 7.6 Hz, 2H, 3,5-H py), 6.93-7.02 (m, 8H, C$_{arom.}$*H*), 7.24 (t, $^3J_{H\text{-}H}$ = 7.6 Hz, 1H, 4-H py), 7.33 (t, $^3J_{H\text{-}H}$ = 7.1 Hz, 1H, 3-C$_{arom.}$*H* SiPh), 7.45 (m, 1H, C$_{arom.}$*H* SiPh), 7.50 (m, 2H, C$_{arom.}$*H* SiPh), 7.76 (m, 2H, C$_{arom.}$*H* Ph). 8.57 (d, $^3J_{H\text{-}H}$ = 7.1 Hz, 1H, 2-C$_{arom.}$*H* SiPh). **^{13}C{^{1}H} NMR** (126 MHz, C_6D_6, 298 K): δ(ppm) = 15.1 (NCH$_2$-*C*H$_3$), 16.3 (Si(*C*H$_3$)$_2$), 25.7 (d, $^1J_{C\text{-}P}$ = 16.5 Hz, P(*C*H$_3$)$_3$), 31.7 (NC(*C*H$^A_3$)$_3$), 32.5 (NC(*C*H$^B_3$)$_3$), 38.8 (N*C*H$_2$-CH$_3$), 53.6 (N*C*(CH$^B_3$)$_3$), 54.0 (N*C*(CH$^A_3$)$_3$), 94.3 (3,5-C$_{arom.}$ py), 125.0 (C$_{arom.}$ Si*Ph*), 126.0 (C$_{arom.}$), 126.1 (C$_{arom.}$ Si*Ph*), 126.9 (C$_{arom}$), 128.4 (C$_{arom}$), 128.6 (C$_{arom.}$), 129.6 (C$_{arom.}$), 130.9 (C$_{arom.}$), 132.8 (4-C$_{arom.}$ py), 133.3 (C$_{arom.}$), 134.3 (C$_{arom.}$), 136.4 (o-C$_{arom.}$ Si*Ph*), 160.5 (C$_{arom.}$ quaternary Si*Ph*) 168.3 (2,6-C$_{arom.}$ py), 171.8 (N*C*N). **^{29}Si NMR, ^{1}H-^{29}Si HMQC NMR** (500 MHz / 99 MHz, C_6D_6, 298 K): δ(ppm) = 31.1 (SiMe$_2$Ph), 77.2 (Si:→Fe). **^{31}P{^{1}H} NMR** (202 MHz, C_6D_6, 298 K): δ(ppm) = 14.7. **IR (KBr pellet, cm^{-1}):** $\tilde{\nu}$ /cm^{-1} = 2020. **ESI-MS (m/z):** calcd for [$C_{50}H_{79}FeN_7PSi_3^+$] (M – H)$^+$ 948.47863; found 948.54423.

3.1.3 *Si* = $MePh_2Si$ (7c)

^{1}H NMR (500 MHz, C_6D_6, 298 K): δ(ppm) = –13.69 (br s, 1H, Fe–H), 1.06 (s, 18H, NC(CH^A_3)$_3$), 1.24 (s, 18H, NC(CH^B_3)$_3$), 1.34 (m, 6H, NCH'H-CH_3), 1.36 (m, 9H, P(CH_3)$_3$), 1.37 (m, 3H, SiCH_3Ph$_2$), 3.33 (dq, $^2J_{H\text{-}H}$ = 13.0 Hz, $^3J_{H\text{-}H}$ = 6.0 Hz, 2H, NCH'*H*-CH_3), 3.56 (dq, $^2J_{H\text{-}H}$ = 13.0 Hz, $^3J_{H\text{-}H}$ = 6.5 Hz, 2H, NC*H'*H-CH_3), 5.86 (d, $^3J_{H\text{-}H}$ = 7.8 Hz, 2H, 3-5-H py), 6.90-7.05 (m, 10H, C$_{arom.}$*H*), 7.24 (t, $^3J_{H\text{-}H}$ = 7.8 Hz, 1H, 4-H py), 7.34 (t, $^3J_{H\text{-}H}$ = 7.2 Hz, 4H, 3-C$_{arom.}$*H* SiPh), 7.71 (d, $^3J_{H\text{-}H}$ = 6.7 Hz, 2H, C$_{arom}$*H* SiPh). 8.22 (d, $^3J_{H\text{-}H}$ = 6.7 Hz, 4H, 2-C$_{arom.}$*H* SiPh). **^{13}C{^{1}H} NMR** (126 MHz, C_6D_6, 298 K): δ(ppm) = 15.0 (NCH$_2$-*C*H$_3$), 25.8 (d, $^1J_{C\text{-}P}$ = 16.7 Hz, P(*C*H$_3$)$_3$), 29.2 (Si*C*H$_3$Ph$_2$), 31.7 (NC(*C*H$^A_3$)$_3$), 32.4 (NC(*C*H$^B_3$)$_3$), 38.7 (N*C*H$_2$-CH$_3$), 53.7 (N*C*(CH$^B_3$)$_3$), 54.1 (N*C*(CH$^A_3$)$_3$), 94.3 (3,5-C$_{arom.}$ py), 125.0 (C$_{arom.}$ Si*Ph*), 126.2 (C$_{arom.}$ SiPh), 126.8 (C$_{arom.}$), 128.3 (C$_{arom.}$), 129.1 (2×C$_{arom.}$ Si*Ph*), 129.8 (C$_{arom.}$), 130.7 (4-C$_{arom.}$ py), 133.2 (C$_{arom.}$), 135,6 (C$_{arom.}$), 136.4 (o-C$_{arom}$ Si*Ph*), 137.2 (C$_{arom.}$), 159.8 (C$_{arom.}$ quaternary Si*Ph*), 168.5 (2,6-C$_{arom.}$ py), 172.3 (N*C*N). **^{29}Si NMR, ^{1}H-^{29}Si HMQC** (500 MHz / 99 MHz, C_6D_6, 298 K): δ(ppm) = 34.4 (SiMePh$_2$), 76.3 (Si:→Fe). **^{31}P{^{1}H} NMR** (202 MHz, C_6D_6, 298 K): δ(ppm) = 16.8.

3.2 Crystallographic Data for 7b

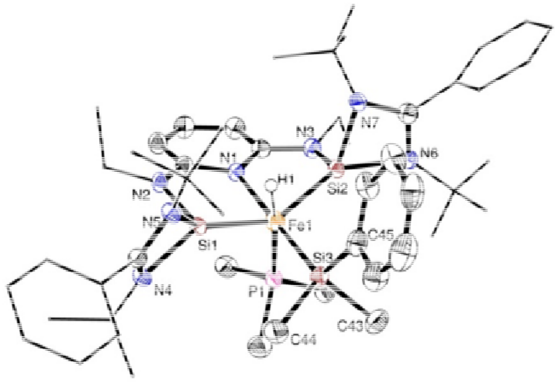

Table S3. Crystallographic data for compound **7b**.

Empirical formula	$2\times(C_{50}H_{80}FeN_7PSi_3) \cdot C_7H_8$
M_r	1992.73
Space group	P-1
Crystal system	Triclinic
a [Å]	11.5288(5)
b [Å]	12.5203(5)
c [Å]	21.1550(11)
α [°]	73.555(4)
β [°]	75.604(4)
γ [°]	74.650(4)
V [Å^3]	2774.0(2)
Z	1
ρ_{calcd} [mg m^{-3}]	1.193
Wavelength [Å]	1.54184
μ ($Mo_{K\alpha}$) [mm^{-1}]	3.379
crystal size [mm^3]	0.45 × 0.12 × 0.04
Θ limits [°]	3.76 to 67.50
completeness to Θ = 67.50° [%]	99.9
reflns measured[a]	18617
independent reflns	9988 [R(int) = 0.0490]
parameters	645
R_1 (R_1 all data)[b]	0.0452 (0.0635)
wR_2 (wR_2 all data)[c]	0.1026 (0.1162)
GOF	1.020
max., min. peaks [eÅ^{-3}]	0.475 and -0.389

[a] Observation criterion: $I>2\sigma(I)$. [b] $R_1 = \Sigma \mid |F_o| - |F_c| \mid / \Sigma |F_o|$. [c] $wR_2 = \{\Sigma[w\text{-}(F_o^2 - F_c^2)^2]/\Sigma[w(F_o^2)^2]\}^{1/2}$

Table S4. Bond lengths [Å] and angles [°] for compound **7b**.

Fe(1)-N(1)	2.063(2)	N(7)-C(26)	1.489(3)
Fe(1)-Si(1)	2.1509(7)	C(8)-C(9)	1.523(4)
Fe(1)-Si(2)	2.1715(7)	C(10)-C(19)	1.500(3)
Fe(1)-P(1)	2.1975(8)	C(11)-C(13)	1.515(4)
Fe(1)-Si(3)	2.2986(8)	C(11)-C(14)	1.528(4)
Si(1)-N(2)	1.786(2)	C(11)-C(12)	1.532(4)
Si(1)-N(5)	1.893(2)	C(15)-C(17)	1.523(4)
Si(1)-N(4)	1.927(2)	C(15)-C(16)	1.529(4)
Si(1)-C(10)	2.364(2)	C(15)-C(18)	1.536(4)
Si(2)-N(3)	1.794(2)	C(19)-C(20)	1.389(4)
Si(2)-N(6)	1.892(2)	C(19)-C(24)	1.397(4)
Si(2)-N(7)	1.916(2)	C(20)-C(21)	1.392(4)
Si(2)-C(25)	2.356(3)	C(21)-C(22)	1.382(4)
Si(3)-C(43)	1.921(3)	C(22)-C(23)	1.375(4)
Si(3)-C(44)	1.925(3)	C(23)-C(24)	1.385(4)
Si(3)-C(45)	1.938(3)	C(25)-C(34)	1.498(4)
P(1)-C(40)	1.839(3)	C(26)-C(27)	1.519(4)
P(1)-C(41)	1.839(3)	C(26)-C(29)	1.526(4)
P(1)-C(42)	1.839(3)	C(26)-C(28)	1.531(4)
N(1)-C(1)	1.371(3)	C(30)-C(33)	1.516(4)
N(1)-C(5)	1.376(3)	C(30)-C(31)	1.519(5)
C(1)-N(2)	1.374(3)	C(30)-C(32)	1.532(4)
C(1)-C(2)	1.402(4)	C(34)-C(35)	1.379(4)
N(2)-C(6)	1.463(3)	C(34)-C(39)	1.394(4)
C(2)-C(3)	1.371(4)	C(35)-C(36)	1.390(4)
N(3)-C(5)	1.375(3)	C(36)-C(37)	1.380(5)
N(3)-C(8)	1.467(3)	C(37)-C(38)	1.372(5)
C(3)-C(4)	1.380(4)	C(38)-C(39)	1.394(4)
N(4)-C(10)	1.321(3)	C(45)-C(46)	1.385(4)
N(4)-C(11)	1.485(3)	C(45)-C(50)	1.408(4)
C(4)-C(5)	1.401(4)	C(46)-C(47)	1.393(4)
N(5)-C(10)	1.339(3)	C(47)-C(48)	1.382(5)
N(5)-C(15)	1.489(3)	C(48)-C(49)	1.379(5)
N(6)-C(25)	1.337(4)	C(49)-C(50)	1.385(5)
N(6)-C(30)	1.488(3)	C(51)-C(52)	1.525(18)
C(6)-C(7)	1.528(4)	C(52)-C(53)	1.374(9)
N(7)-C(25)	1.327(3)	C(52)-C(57)	1.389(10)

C(53)-C(54)	1.386(10)	C(44)-Si(3)-Fe(1)	116.23(9)
C(54)-C(55)	1.362(10)	C(45)-Si(3)-Fe(1)	117.61(10)
C(55)-C(56)	1.373(9)	C(40)-P(1)-C(41)	96.02(15)
C(56)-C(57)	1.370(10)	C(40)-P(1)-C(42)	95.81(15)
		C(41)-P(1)-C(42)	99.13(15)
N(1)-Fe(1)-Si(1)	82.72(6)	C(40)-P(1)-Fe(1)	125.45(10)
N(1)-Fe(1)-Si(2)	78.76(6)	C(41)-P(1)-Fe(1)	120.69(11)
Si(1)-Fe(1)-Si(2)	144.54(3)	C(42)-P(1)-Fe(1)	114.18(10)
N(1)-Fe(1)-P(1)	89.22(6)	C(1)-N(1)-C(5)	117.8(2)
Si(1)-Fe(1)-P(1)	105.82(3)	C(1)-N(1)-Fe(1)	121.12(16)
Si(2)-Fe(1)-P(1)	103.90(3)	C(5)-N(1)-Fe(1)	120.92(16)
N(1)-Fe(1)-Si(3)	176.87(6)	N(1)-C(1)-N(2)	115.6(2)
Si(1)-Fe(1)-Si(3)	95.53(3)	N(1)-C(1)-C(2)	121.4(2)
Si(2)-Fe(1)-Si(3)	101.41(3)	N(2)-C(1)-C(2)	123.0(2)
P(1)-Fe(1)-Si(3)	93.76(3)	C(1)-N(2)-C(6)	118.7(2)
N(2)-Si(1)-N(5)	102.79(10)	C(1)-N(2)-Si(1)	115.61(16)
N(2)-Si(1)-N(4)	98.58(9)	C(6)-N(2)-Si(1)	125.60(17)
N(5)-Si(1)-N(4)	68.26(9)	C(3)-C(2)-C(1)	119.0(2)
N(2)-Si(1)-Fe(1)	103.33(7)	C(5)-N(3)-C(8)	118.1(2)
N(5)-Si(1)-Fe(1)	133.05(7)	C(5)-N(3)-Si(2)	112.63(17)
N(4)-Si(1)-Fe(1)	142.90(8)	C(8)-N(3)-Si(2)	125.58(18)
N(2)-Si(1)-C(10)	105.36(9)	C(2)-C(3)-C(4)	120.4(3)
N(5)-Si(1)-C(10)	34.47(9)	C(10)-N(4)-C(11)	130.2(2)
N(4)-Si(1)-C(10)	33.94(9)	C(10)-N(4)-Si(1)	91.49(16)
Fe(1)-Si(1)-C(10)	151.06(7)	C(11)-N(4)-Si(1)	138.31(17)
N(3)-Si(2)-N(6)	103.59(10)	C(3)-C(4)-C(5)	119.1(2)
N(3)-Si(2)-N(7)	98.86(11)	C(10)-N(5)-C(15)	127.9(2)
N(6)-Si(2)-N(7)	68.64(9)	C(10)-N(5)-Si(1)	92.40(17)
N(3)-Si(2)-Fe(1)	102.13(7)	C(15)-N(5)-Si(1)	137.99(16)
N(6)-Si(2)-Fe(1)	145.31(8)	N(3)-C(5)-N(1)	115.0(2)
N(7)-Si(2)-Fe(1)	129.19(7)	N(3)-C(5)-C(4)	123.9(2)
N(3)-Si(2)-C(25)	106.27(10)	N(1)-C(5)-C(4)	121.1(2)
N(6)-Si(2)-C(25)	34.55(10)	C(25)-N(6)-C(30)	128.0(2)
N(7)-Si(2)-C(25)	34.28(9)	C(25)-N(6)-Si(2)	92.07(16)
Fe(1)-Si(2)-C(25)	149.13(7)	C(30)-N(6)-Si(2)	137.62(18)
C(43)-Si(3)-C(44)	98.63(14)	N(2)-C(6)-C(7)	113.4(2)
C(43)-Si(3)-C(45)	99.22(13)	C(25)-N(7)-C(26)	129.5(2)
C(44)-Si(3)-C(45)	99.58(13)	C(25)-N(7)-Si(2)	91.35(17)
C(43)-Si(3)-Fe(1)	121.55(10)	C(26)-N(7)-Si(2)	138.81(17)

N(3)-C(8)-C(9)	113.4(3)	C(27)-C(26)-C(29)	109.6(2)
N(4)-C(10)-N(5)	107.4(2)	N(7)-C(26)-C(28)	111.1(2)
N(4)-C(10)-C(19)	125.1(2)	C(27)-C(26)-C(28)	110.7(3)
N(5)-C(10)-C(19)	127.5(2)	C(29)-C(26)-C(28)	108.5(3)
N(4)-C(10)-Si(1)	54.57(12)	N(6)-C(30)-C(33)	105.4(2)
N(5)-C(10)-Si(1)	53.13(13)	N(6)-C(30)-C(31)	111.8(2)
C(19)-C(10)-Si(1)	174.43(19)	C(33)-C(30)-C(31)	109.5(3)
N(4)-C(11)-C(13)	106.6(2)	N(6)-C(30)-C(32)	110.3(2)
N(4)-C(11)-C(14)	112.7(2)	C(33)-C(30)-C(32)	108.4(3)
C(13)-C(11)-C(14)	108.2(2)	C(31)-C(30)-C(32)	111.3(3)
N(4)-C(11)-C(12)	110.5(2)	C(35)-C(34)-C(39)	119.7(3)
C(13)-C(11)-C(12)	110.0(2)	C(35)-C(34)-C(25)	119.0(3)
C(14)-C(11)-C(12)	108.8(2)	C(39)-C(34)-C(25)	121.2(3)
N(5)-C(15)-C(17)	110.4(2)	C(34)-C(35)-C(36)	120.5(3)
N(5)-C(15)-C(16)	107.9(2)	C(37)-C(36)-C(35)	119.4(3)
C(17)-C(15)-C(16)	109.7(2)	C(38)-C(37)-C(36)	120.7(3)
N(5)-C(15)-C(18)	111.4(2)	C(37)-C(38)-C(39)	120.2(3)
C(17)-C(15)-C(18)	110.5(3)	C(34)-C(39)-C(38)	119.5(3)
C(16)-C(15)-C(18)	106.8(3)	C(46)-C(45)-C(50)	115.3(3)
C(20)-C(19)-C(24)	119.4(2)	C(46)-C(45)-Si(3)	123.7(2)
C(20)-C(19)-C(10)	120.3(2)	C(50)-C(45)-Si(3)	121.0(2)
C(24)-C(19)-C(10)	120.2(2)	C(45)-C(46)-C(47)	123.2(3)
C(19)-C(20)-C(21)	119.8(3)	C(48)-C(47)-C(46)	119.6(3)
C(22)-C(21)-C(20)	120.3(3)	C(49)-C(48)-C(47)	119.2(3)
C(23)-C(22)-C(21)	120.0(3)	C(48)-C(49)-C(50)	120.2(3)
C(22)-C(23)-C(24)	120.3(3)	C(49)-C(50)-C(45)	122.4(3)
C(23)-C(24)-C(19)	120.1(3)	C(53)-C(52)-C(57)	119.3(7)
N(7)-C(25)-N(6)	107.4(2)	C(53)-C(52)-C(51)	123.4(11)
N(7)-C(25)-C(34)	126.8(3)	C(57)-C(52)-C(51)	117.2(11)
N(6)-C(25)-C(34)	125.7(2)	C(52)-C(53)-C(54)	120.1(7)
N(7)-C(25)-Si(2)	54.37(14)	C(55)-C(54)-C(53)	120.3(7)
N(6)-C(25)-Si(2)	53.39(13)	C(54)-C(55)-C(56)	119.6(7)
C(34)-C(25)-Si(2)	172.0(2)	C(57)-C(56)-C(55)	120.9(7)
N(7)-C(26)-C(27)	111.1(2)	C(56)-C(57)-C(52)	119.7(7)
N(7)-C(26)-C(29)	105.7(2)		

4 Mechanistic Experiments

4.1 Kinetics: Iron(0) complex 2 vs. Iron(II) complex 7a

4.1.1 Iron(0) complex 2

Iron(0) complex **2** (5 mg, 0.005 mmol, 2 mol%) was weighed in a Schlenk flask together with silane **4a** (55 mg, 0.33 mmol, 1.5 equiv) and ketone **3a** (34 mg, 0.22 mmol, 1.0 equiv). 2.0 mL of THF were added through a syringe and the mixture was heated to 70 °C. Aliquots were taken during the course of the reaction, hydrolysed and analysed by GC-MS (red squares).

4.1.2 Iron(II) complex 7a

Iron(0) complex **2** (5 mg, 0.005 mmol, 2 mol%) was weighed in a Schlenk flask together with silane **4a** (55 mg, 0.33 mmol, 1.5 equiv). 2.0 mL of THF was added through a syringe and the mixture was heated to 70 °C for 30 min while the colour changed from dark purple to clear orange. Ketone **3a** (34 mg, 0.22 mmol, 1.0 equiv) was added into the reaction mixture and the heating was continued at 70 °C. Aliquots were taken during the course of the reaction, hydrolysed and analysed by GC-MS (blue diamonds).

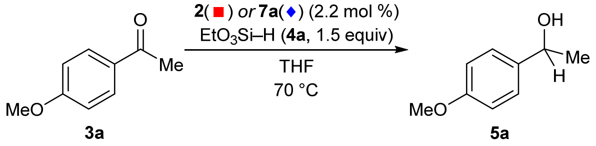

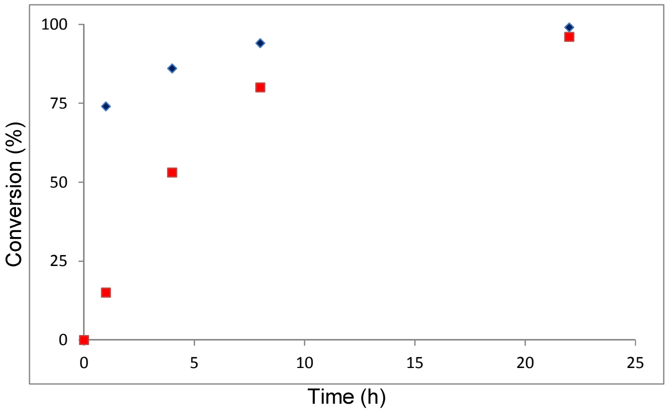

4.2 Scrambling at the Si–H Bond

4.2.1 Hydrosilane *Si*−D/Fe−H Scrambling Using Me_2PhSi–D (4b-d_1)

Complex **7b** (54 mg, 0.057 mmol, 1.0 equiv) was mixed in a Schlenk flask with deuterated dimethylphenylsilane (**4b**-d_1, 9.5 mg, 0.069 mmol, 1.2 equiv) in 2.0 mL of THF. The reaction mixture was heated at 70 °C in an oil bath. The course of the reaction was followed by analysis of an aliquot (0.1 mL) by GC-MS. The deuterated dimethylphenylsilane/dimethylphenylsilane ratio (*Si*−D/*Si*−H, blue diamonds) was calculated according to the intensity of peaks at 122/121 $[M–CH_3]^+$ in the mass spectrum for the GC peak corresponding to the dimethylphenylsilane **4b**.

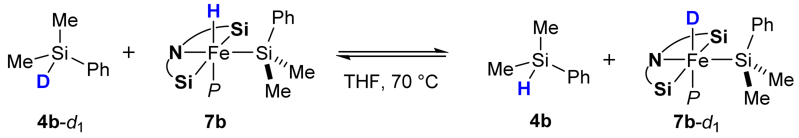

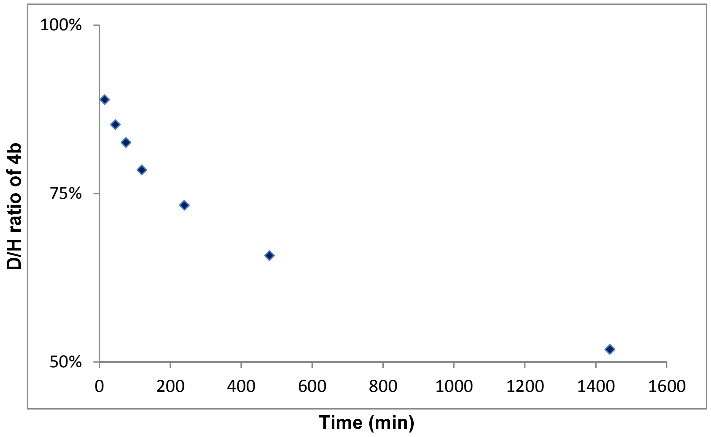

4.2.2 Deuteration Studies with Me_2PhSi–D (4b-d_1)

Complex **7b** (49 mg, 0.051 mmol, 0.88 equiv) was mixed in a Schlenk flask with acetophenone (**3e**, 9.2 mg, 0.077 mmol, 1.3 equiv) and deuterated dimethylphenylsilane (**4b**-d_1, 8.0 mg, 0.058 mmol, 1.0 equiv) in 2.0 mL of THF. The reaction mixture was heated at 70 °C in an oil bath. The course of the reaction was followed by analysis of aliquots (0.1 mL) by GC-MS. The D/H ratio on the product was calculated according to the intensity of peaks at 242/241 $[M–CH_3]^+$ in the mass spectrum for the GC peak corresponding to the silyl ether product **8eb**.

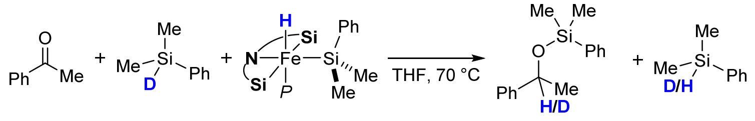

3e **4b**-d_1 **7b** **8eb** **4b**

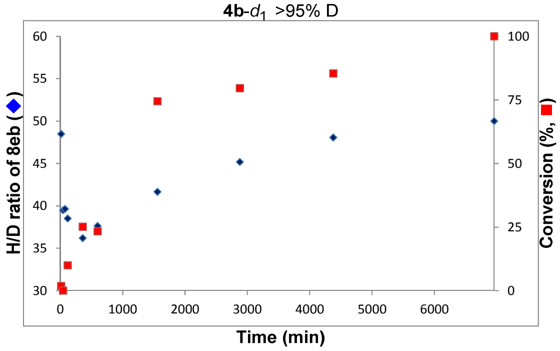

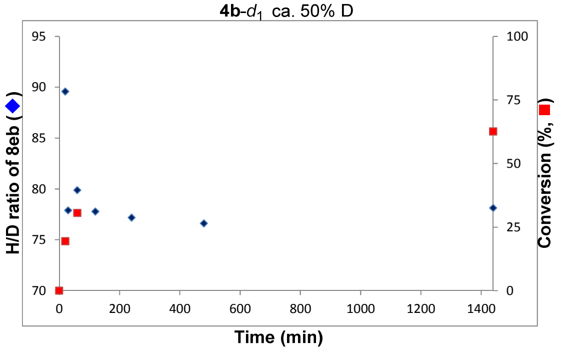

4.3 Scrambling at the C–H Bond

4.3.1 Scrambling Experiment with Deuterated Silyl Ether (8eb-d_1)

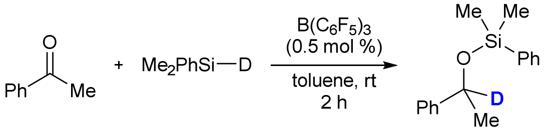

3e **4b-d_1** **8eb-d_1**

According to a literature procedure,[S21] a 2-mL vial was charged with acetophenone (**3e**, 100 mg, 0.84 mmol, 1.0 equiv), deuterated dimethylphenylsilane (**4b**-d_1, 120 mg, 0.87 mmol, 1.0 equiv), and $B(C_6F_5)_3$ (2 mg, 3 µmol, 0.5 mol %) in toluene (0.5 mL). The reaction mixture was stirred at room temperature for 2 h and then subjected directly to flash column chromatography on silica gel using cyclohexane/ethyl acetate (30:1) as eluent, yielding the silyl ether **8eb**-d_1 (180 mg, 0.71 mmol, 84%, >95% D) as a colourless oil.

^{1}H NMR (500 MHz, $CDCl_3$, 298 K): δ(ppm) = 0.29 (s, 3H, Si−*Me*), 0.34 (s, 3H, Si−*Me*), 1.42 (s, 3H, C−*Me*), 7.21–7.39 (m, 8H, Ar), 7.55–7.57 (m, 2H, Ar). **^{13}C{^{1}H} NMR** (126 MHz, $CDCl_3$, 298 K): δ(ppm) = –1.2, –0.7, 26.8, 70.8 (t, $^1J_{C–D}$ = 21.7 Hz), 127.9, 129.7, 133.1, 138.3, 146.3. **^{29}Si DEPT NMR** (99 MHz, $CDCl_3$, 298 K): δ(ppm) = 6.6. **R$_f$** = 0.5 (cyclohexane:ethyl acetate 30:1). **IR** (ATR): $\tilde{\nu}$ /cm^{-1}= 3066 (w), 3023 (w), 2970 (m), 2924 (w), 2130 (w), 1427 (m), 1368 (m), 1251 (s), 1137 (s), 1115 (s), 1095 (m), 1010 (s), 861 (m), 820 (s), 783 (s), 695 (s). **HRMS** (APCI) for $C_{16}H_{20}DOSi$ [$(M+H)^+$]: calcd 258.1419, found 258.1462.

Deuterated silyl ether **8eb**-*d*$_1$ (13 mg, 0.049 mmol, 1.1 equiv) and iron hydride complex **7b** (43 mg, 0.045 mmol, 1.0 equiv) were dissolved in THF (2.0 mL), stirred at 70 °C, and the reaction was followed by analysis of an aliquot (0.1 mL) by GC-MS. The H/D ratio on the product was calculated according to the intensity of peaks at 242/241 $[M–CH_3]^+$ in the mass spectrum for the GC peak corresponding to the silyl ether product **8eb**.

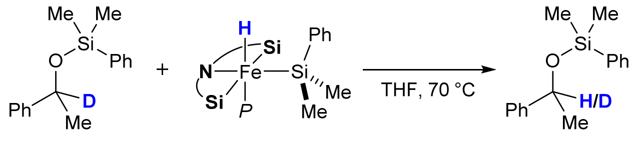

8eb-*d*$_1$ **7b** **8eb**

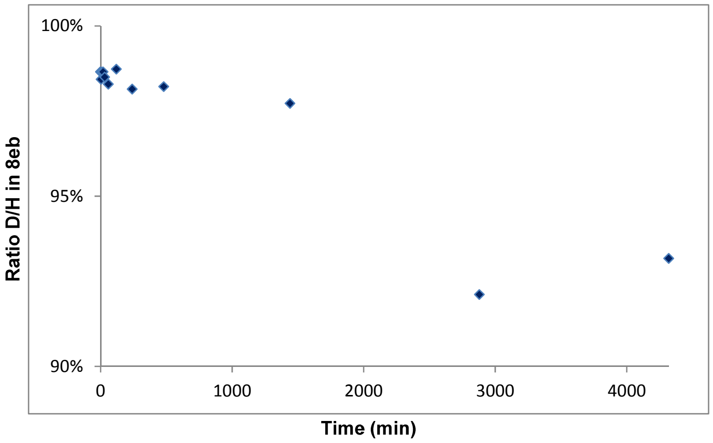

4.3.2 Racemisation Experiment with Enantiopure Silyl Ether (*S*)-8eb

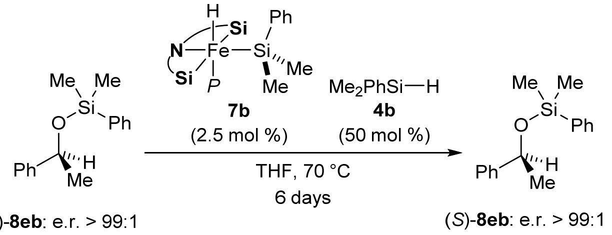

The enantiomerically enriched silyl ether product (*S*)-**8eb** (49 mg, 0.19 mmol, 1.0 equiv, e.r. > 99:1) was subjected to the catalytic conditions in the presence of the dimethylphenylsilane (**4b**, 13 mg, 90 µmol, 0.47 equiv) and the iron hydride complex **7b** (4.8 mg, 5.0 µmol, 2.5 mol %). The reaction mixture was heated up for 6 days at 70 °C, and aliquots passed through a short plug of silica gel and analysed by chiral HPLC analysis showed no racemization during the course of the reaction.

4.4 Phosphine Scrambling

4.4.1 Synthesis of Trimethylphosphine-d_9 (6-d_9)

According to a literature procedure,[S23] in a 100 mL Schlenk flask magnesium turnings (2.3 g, 90 mmol, 3.8 equiv) were thermally and mechanically activated under vacuum. Freshly degassed di-*n*-butyl ether (15 mL) was added followed by rapid addition of methyl iodide-d_3 (0.36 mL, 5.6 mmol, 0.30 equiv) under nitrogen atmosphere at room temperature. After the initiation of the reaction (colour change to dark brown and heat formation), the solution was cooled to 0 °C and the rest of methyl iodide-d_3 (3.3 mL, 50 mmol, 3.0 equiv) was added dropwise. The solution was allowed to warm to rt and stirred for additional 3 h. The solution was cooled to 0 °C and a solution of triphenylphosphite (5.0 g, 15 mmol, 1.0 equiv) in di-*n*-butyl ether (40 mL) was added slowly over 2 h. The solution was warmed to rt and stirred for 30 min. The dropping funnel was replaced by a distillation apparatus equipped with a Vigreux column (10 cm) and the desired deuterated phosphine **6**-d_9 (560 mg, 6.6 mmol, 44%) was distilled at 160 °C (oil bath).

bp 38 °C. **^{2}H NMR** (77 MHz, C_6D_6/C_6H_6, 298 K): δ(ppm) = 0.72. **^{13}C{^{1}H} NMR** (176 MHz, C_6D_6/C_6H_6, 298 K): δ(ppm) = 15.4 (dsept, $J_{C\text{-}P}$ = 6.7, $J_{C\text{–}D}$ = 16.4 Hz). **^{31}P NMR** (202 MHz, C_6D_6/C_6H_6, 298 K): δ(ppm) = –65.5.

4.4.2 Scrambling between Complex 7b and Trimethylphosphine-d_9 (6-d_9)

Complex **7b** (10 mg, 0.011 mmol 1.0 equiv) was mixed with trimethylphosphine-d_9 (**6**-d_9, 2.5 mg, 0.029 mmol, 2.7 equiv) in C_6D_6. The sample was closed under nitrogen in a Young NMR tube, heated to 70 °C, and monitored by ^{1}H and ^{2}H NMR spectroscopy. Slow formation of **7b**-d_9 was observed.

Selected NMR data for **7b**-d_9

^{1}H,^{31}P HMQC NMR (500 MHz / 203 MHz, C_6H_6/C_6D_6, 298 K): δ(^{1}H) = –13.9 / δ(^{31}P) = 12.4 ppm.

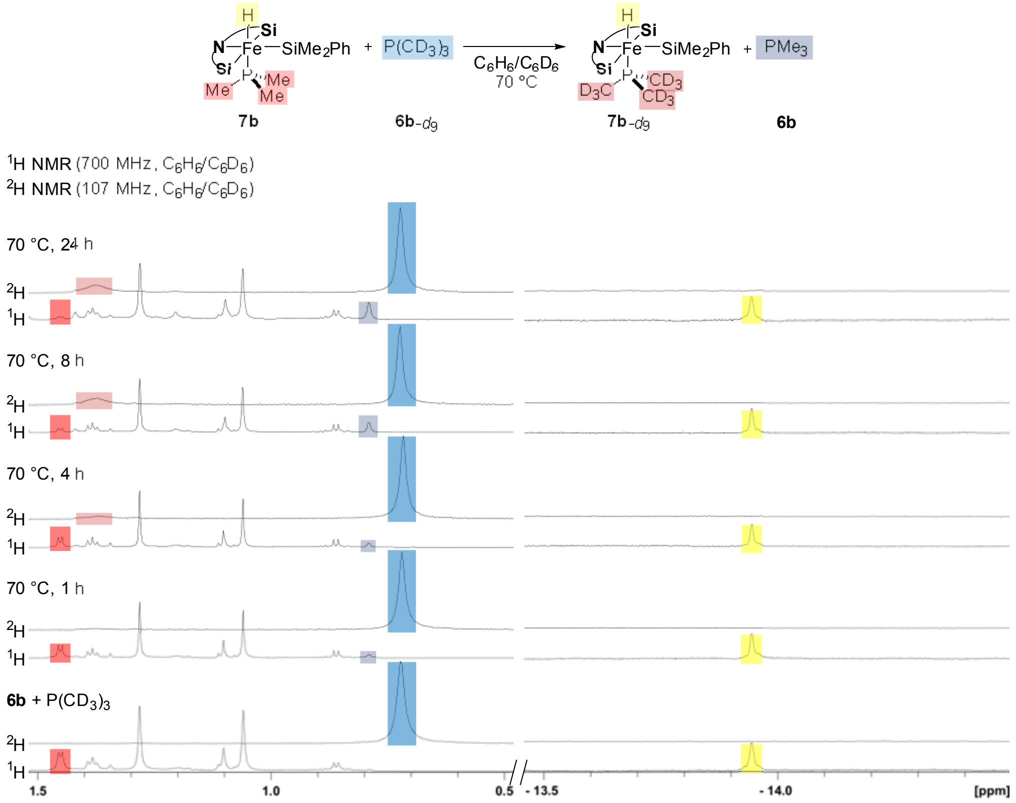

4.5 Phosphine Dissociation

Complex **7b** (5 mg, 0.005 mmol) was dissolved in THF (2.0 mL). The closed system was heated up to 70 °C for a period of 2 h. The sample was frozen and the gas phase was changed by 3 purge-cycles with N_2 while thawing the sample. This procedure was repeated 3 times with the same period of time between each other. After 8 h, all volatiles were removed *in vacuo* and the sample was dissolved in 0.5 mL of C_6D_6 for ^{1}H and ^{31}P{^{1}H} NMR analyses.

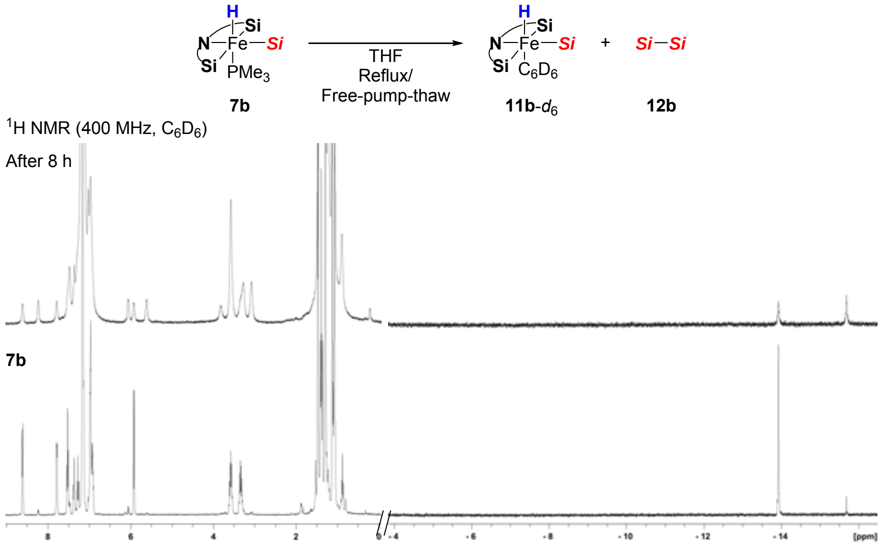

4.5.1 Phospine Dissociation in the Presence of Acetophenone (3e)

Complex **7b** (5 mg, 0.005 mmol, 1 equiv) was dissolved in THF (2.0 mL). Acetophenone (**3e**, 2.0 mg, 0.02 mmol, 4 equiv) was added and the closed system was heated up to 70 °C for a period of 2 h. The sample was frozen and the gas phase was changed by 3 purge-cycles with N_2 while thawing the sample. This procedure was repeated 3 times with the same period of time between each other. After 8 h, all volatiles were removed *in vacuo* and the sample was dissolved in 0.5 mL of C_6D_6 for ^1H and $^{31}P\{^1H\}$ NMR analyses.

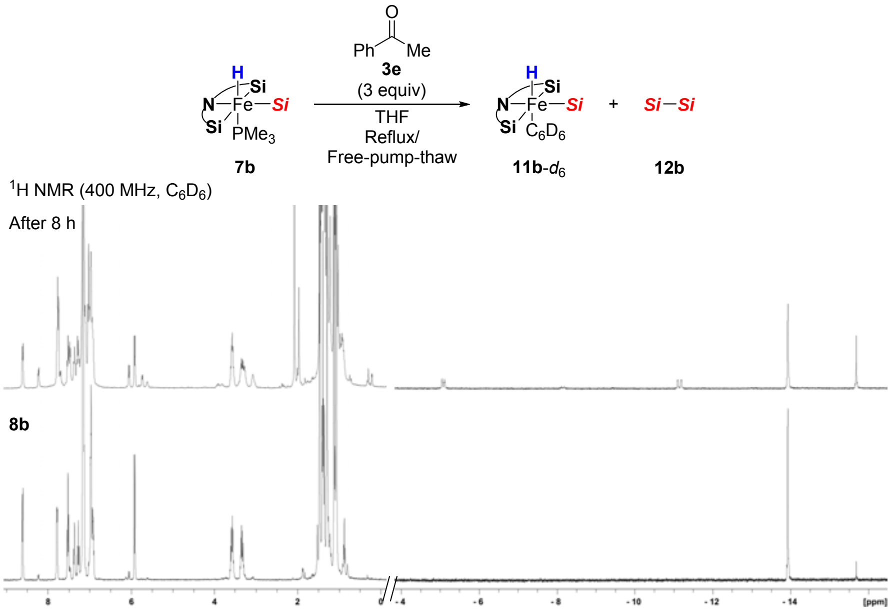

4.6 Silyl Scrambling

Complex **7b** (10 mg, 0.011 mmol 1.0 equiv) was mixed with methyldiphenylsilane **4c** (4.0 mg, 0.020 mmol, 1.8 equiv) in C_6D_6. The sample was closed under nitrogen in a Young NMR tube, heated to 70 °C, and monitored by ^{1}H spectroscopy.

Selected NMR data for **11b**-d_6:

^{1}H NMR (500 MHz, C_6D_6, 298 K): δ = –15.69 (s, $^2J_{H\text{-}Si}$ = 23.8 Hz, 1H, Fe–H) ppm. **^{1}H-^{29}Si HMQC NMR** (500 MHz / 99 MHz, C_6D_6, 298 K): δ(^{1}H) = –15.7 / δ(^{29}Si) = 22.2 ($SiMe_2Ph$), δ(^{1}H) = –15.7 / δ(^{29}Si) = 84.5 (Si:→Fe) ppm.

Selected NMR data for **11c**-d_6:

^{1}H NMR (500 MHz, C_6D_6, 298 K): δ = –15.50 (s, $^2J_{H\text{-}Si}$ = 23.7 Hz, 1H, Fe–H) ppm. **^{1}H,^{29}Si HMQC NMR** (500 MHz / 99 MHz, C_6D_6, 298 K): δ(^{1}H) = –15.5 / δ(^{29}Si) = 27.7 ($SiMe_2Ph$), δ(^{1}H) = –15.5 / δ(^{29}Si) = 83.0 (Si:→Fe) ppm.

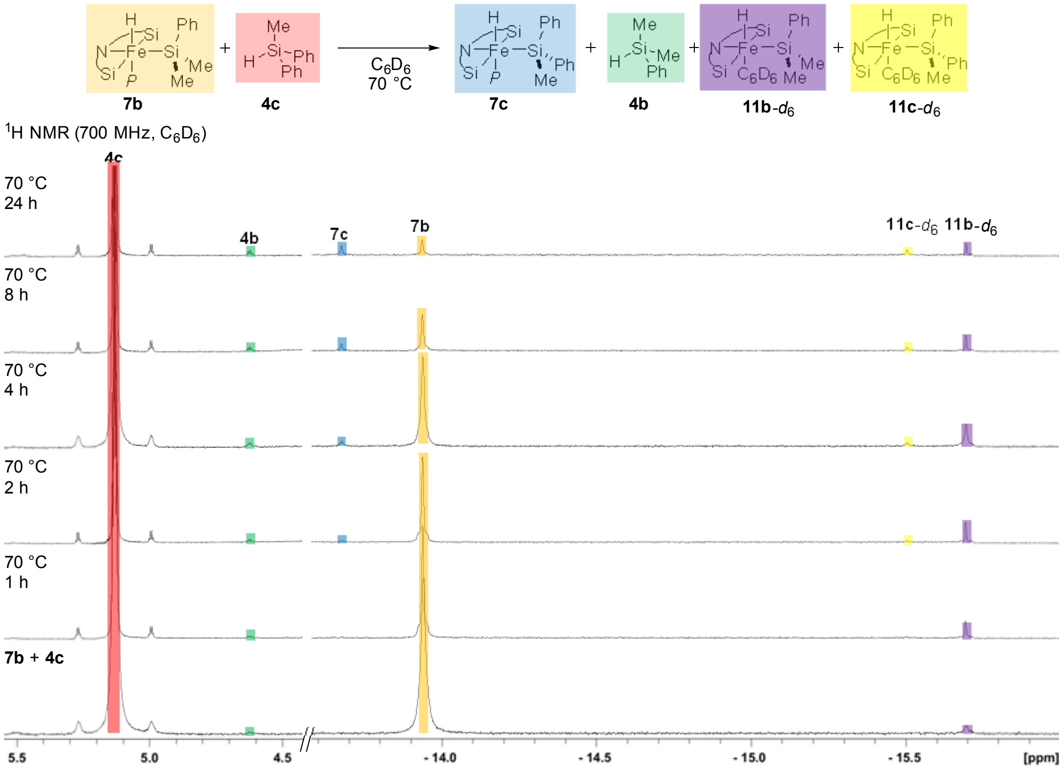

Time (h)	7b	7c	11c-d_6	11b-d_6
	**Iron Hydride Product Distribution**			
0	96	-	-	4
1	91	-	-	9
2	85	1	<1	13
4	79	3	2	16
8	68	10	5	17
24	44	25	12	19

4.7 Silane Cross-over Experiment

Complex **7b** (48 mg, 0.050 mmol, 1.00 equiv) was mixed in a Schlenk flask with acetophenone (**3e**, 8.8 mg, 0.073 mmol, 1.5 equiv), and methyldiphenylsilane (**4c**, 11 mg, 0.053 mmol, 1.1 equiv) in 2.0 mL of THF. The reaction mixture was heated at 70 °C with an oil bath. The course of the reaction was followed by analysis of aliquots (0.1 mL) by GC-MS.

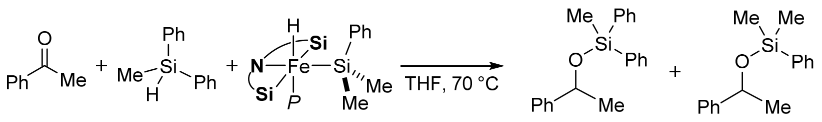

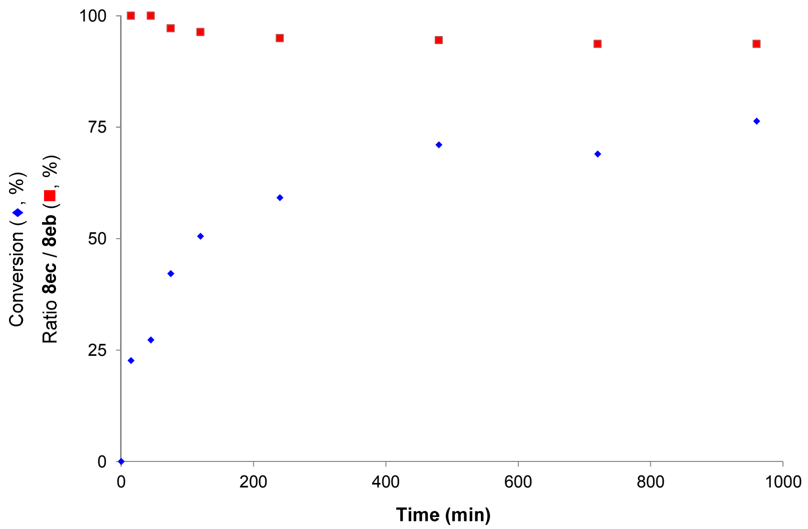

4.8 Hydrosilylation with Silicon-Stereogenic Hydrosilane

4.8.1 Catalytic Reaction between Acetophenone (3e) with Enantioenriched Acyclic Hydrosilane (^{Si}S)-4d

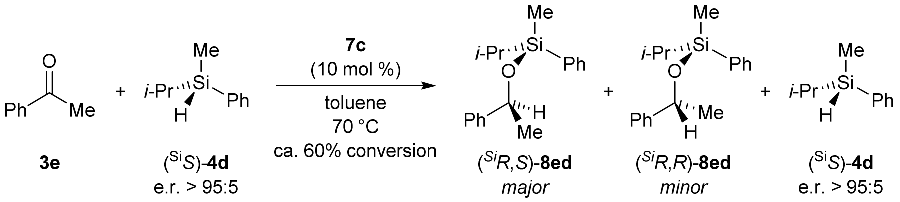

Acetophenone (**3e**, 60 mg, 0.50 mmol, 1.0 equiv), (^{Si}S)-isopropylmethylphenylsilane [(^{Si}S)-**4d**, 82 mg, 0.50 mmol, 1.0 equiv, e.r. > 95:5], and the iron hydride complex **7b** (49 mg, 0.052 mmol, 10 mol %) were dissolved in 3 mL of toluene. The reaction mixture was maintained at 70 °C in an oil bath for 6 days reaching ca. 60% conversion. Purification by flash column chromatography using *n*-pentane:diethyl ether (100:1) as eluting solvent mixture gave silyl ether **8ed** (44 mg, 0.15 mmol, 31% yield, d.r. = 56:44) and unreacted hydrosilane (^{Si}S)-**4d** (10 mg, 0.07 mmol, 15%, e.r. > 95:5).[S24]

4.8.2 Reductive Si–O Bond Cleavage of Silyl Ether 8ed

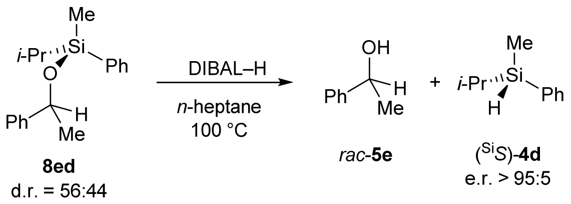

A Schlenk tube equipped with a magnetic stirrer and a reflux condenser was charged with a solution of the silyl ether **8ed** (25 mg, 0.089 mmol, 1.0 equiv) in *n*-heptane (1.0 mL). DIBAL−H (0.5 mL, 0.5 mmol, 6 equiv, 1.0M in *n*-hexane) was added in one portion at room temperature, and the resulting reaction mixture was heated to reflux and maintained at this temperature for 20 h. The reaction mixture was allowed to cool to room temperature and quenched by careful addition of aqueous HCl (1M, 5 mL). The organic layer was separated, and the aqueous phase was extracted with *tert*-butyl methyl ether (3 × 5 mL). The combined organic layers were washed with brine (5 mL), dried over Na_2SO_4, filtered, and the volatiles were evaporated under reduced pressure. The crude product was purified by flash column chromatography on silica gel using *n*-pentane/diethyl ether mixtures (100:0→10:1) as eluent affording the analytically pure hydrosilane (^{Si}S)-**4d** (9.3 mg, 0.057 mmol, 64%, e.r. > 95:5) as colourless oil.[S24]

4.9 Competition Experiment

In a nitrogen-filled glove box, iron(0) complex **2** (4.4 mg, 2.5 mol %, 5.0 µmol), 4-methoxy-acetophenone (**3a**, 30 mg, 0.20 mmol, 1.0 equiv, red square), 4-trifluoromethylacetophenone (**3f**, 38 mg, 0.20 mmol, 1.0 equiv, blue diamond), and triethoxysilane (**4a**, 53 mg, 0.32 mmol, 1.6 equiv) were weighed in a Schlenk flask containing a magnetic stirrer and dissolved in 4 mL of THF. A sample (ca. 0.3 mL) was taken after mixing the reaction mixture (2 min) and quenched with a KOH solution (5% in H_2O). The flask was taken out of the glove box, stirred and heated to 70 °C with an oil bath. Samples of ca. 0.3 mL were taken every 5 minutes, quenched, and hydrolysed with 1 mL of KOH solution (5% in H_2O) for 1 h. The mixture was extracted with diethyl ether (2 × 1 mL). The combined organic layers were dried with an-hydrous Na_2SO_4 and filtered. The sample was analysed by GC-MS. Integration of the peaks for the ketones and the respective alcohols permitted the evaluation of the conversion for each ketone.

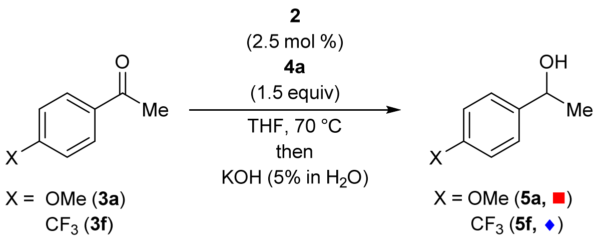
2
(2.5 mol %)
4a
(1.5 equiv)
THF, 70 °C
then
KOH (5% in H2O)
O
Me
X
OH
Me
X
X = OMe (3a)
CF3 (3f)
X = OMe (5a, ■)
CF3 (5f, ◆)

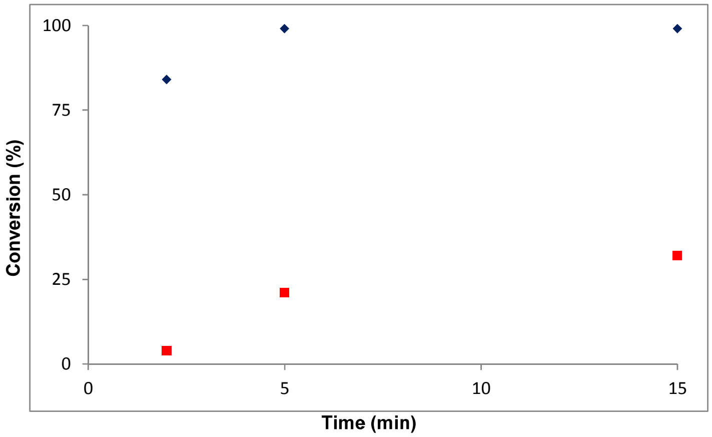
Conversion (%)
Time (min)
0
25
50
75
100
0
5
10
15

5 Cartesian Geometries at ωB97X-D/6-31G(d)[Fe:cc-pVTZ] in Ångstrom [Å]

5.1 Iron(II) Complex 7a

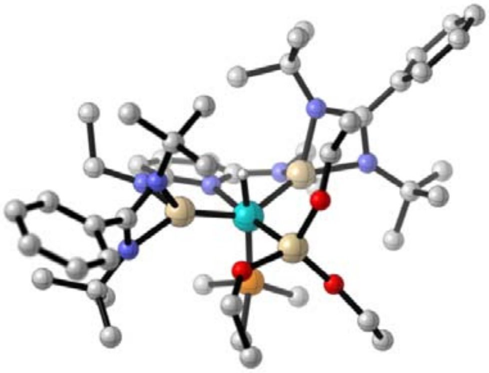

C	5.078283	-2.422990	-1.821078
C	5.170447	-1.740584	-0.595460
C	6.344834	-1.833764	0.168246
C	7.418988	-2.602213	-0.290359
C	7.329255	-3.278457	-1.516092
C	6.157654	-3.187787	-2.279384
C	4.025198	-0.914503	-0.128438
N	2.988076	-1.343686	0.594893
C	2.790386	-2.604194	1.332259
C	1.545203	-2.394496	2.219895
Si	2.059498	0.340764	0.439762
N	3.825089	0.397898	-0.377674
C	4.592059	1.333048	-1.230328
C	3.931620	2.714602	-1.026920
Fe	-0.028991	0.769723	0.196241
P	-0.116212	2.937611	0.482472
C	-1.302226	3.986007	-0.521396
Si	-1.959808	-0.012548	0.588097
N	-1.966958	-0.330477	2.389113
C	-3.033567	-1.008387	3.130094
C	-2.615741	-2.390280	3.656950
N	-2.911095	-1.555775	-0.072287
C	-2.587349	-2.837279	-0.736440
C	-3.569926	-3.968682	-0.355002
C	-4.078999	-0.886507	-0.108913

N	-3.885752	0.323663	0.408563
C	-4.858128	1.346639	0.833957
C	-6.092350	0.737198	1.530024
C	-5.375827	-1.369689	-0.639570
C	-6.218381	-2.166052	0.159253
C	-7.475745	-2.549311	-0.315696
C	-7.900919	-2.140735	-1.590271
C	-7.060218	-1.351970	-2.388163
C	-5.800826	-0.963121	-1.914932
C	-1.179567	-3.268724	-0.289411
C	-2.574492	-2.612568	-2.266418
C	-5.318745	2.209310	-0.357668
C	-4.115583	2.253440	1.829194
Si	-0.199822	0.880666	-2.208302
O	-1.973361	0.849774	-2.272970
C	-3.003846	1.161066	-3.184805
C	-2.957504	2.600596	-3.693530
N	0.185946	0.557449	2.322208
C	-0.874726	0.145439	3.085562
C	-0.840700	0.230818	4.496558
C	0.317822	0.699662	5.110592
C	1.447333	1.011462	4.349704
C	1.370750	0.866961	2.950337
N	2.467969	0.979627	2.120673
C	3.791761	1.049947	2.746847
C	4.200637	2.489256	3.104487
O	0.776424	2.328092	-2.498969
C	1.062167	3.229071	-3.560592
C	1.835400	4.399429	-2.968131
O	0.471022	-0.568649	-2.896750
C	1.377673	-1.469375	-2.326126
C	1.544804	-2.659607	-3.266529
C	4.489339	0.964242	-2.728933
C	6.072281	1.408469	-0.792714
C	1.391804	4.049877	0.450436
C	-0.639529	3.440471	2.220026
C	2.556816	-3.774808	0.352108

C	3.992802	-2.919593	2.249193
H	6.548538	2.265035	-1.300100
H	4.434355	3.466468	-1.658794
H	-0.180292	-0.725658	-0.127063
H	-1.708262	-0.052781	5.089284
H	0.348919	0.811099	6.198117
H	2.372675	1.336450	4.822463
H	-3.885730	-1.126290	2.448935
H	-3.392247	-0.372181	3.962044
H	-2.329803	-3.033199	2.811773
H	-3.450028	-2.869545	4.200127
H	-1.753534	-2.303064	4.335894
H	4.523955	0.643151	2.036253
H	3.828882	0.410796	3.651675
H	4.223345	3.110321	2.196200
H	5.202789	2.507755	3.568967
H	3.478327	2.939337	3.805500
H	-3.822709	1.697125	2.735798
H	-0.438901	-2.501523	-0.544903
H	-0.912955	-4.207803	-0.801178
H	-1.146242	-3.439249	0.795242
H	-2.298802	-3.545771	-2.788343
H	-1.842071	-1.825876	-2.516051
H	-3.674328	-4.032235	0.740231
H	-3.158102	-4.924209	-0.721678
H	-4.563037	-3.831579	-0.805706
H	1.727601	-3.541242	-0.328310
H	2.302893	-4.689020	0.916436
H	3.462859	-3.969409	-0.243253
H	4.876657	-3.232781	1.673535
H	3.717455	-3.738814	2.935642
H	4.245117	-2.031918	2.850898
H	1.768229	-1.685994	3.032553
H	1.248560	-3.358923	2.663073
H	0.711224	-1.987451	1.636052
H	5.082338	0.067525	-2.964041
H	4.873419	1.802627	-3.333712

H	3.438139	0.795779	-3.004554
H	6.136964	1.569263	0.295735
H	6.627906	0.498960	-1.058150
H	2.864282	2.673529	-1.300121
H	4.014880	3.031824	0.022312
H	4.163173	-2.343162	-2.412939
H	6.410593	-1.299602	1.119465
H	1.782877	4.121694	-0.578273
H	2.157497	3.601857	1.100970
H	1.137947	5.060439	0.819669
H	0.183179	3.211954	2.911635
H	-1.511535	2.870956	2.553072
H	-0.860297	4.525362	2.260927
H	-4.447643	2.667223	-0.840022
H	-5.993078	3.006425	-0.001018
H	-5.860787	1.592775	-1.093933
H	-3.213251	2.645717	1.346948
H	-6.756461	0.226550	0.819148
H	-5.781079	0.022967	2.309703
H	-3.571281	-2.294406	-2.612059
H	-5.135730	-0.352437	-2.539068
H	-7.384234	-1.034270	-3.382997
H	-8.885787	-2.438382	-1.960261
H	-8.125610	-3.167542	0.309938
H	-5.884750	-2.469686	1.154321
H	-1.249224	5.047193	-0.212927
H	-2.328575	3.614798	-0.370173
H	-1.039669	3.883624	-1.584948
H	6.079623	-3.714425	-3.234689
H	8.173089	-3.872432	-1.876263
H	8.329158	-2.672937	0.311114
H	-4.761310	3.098578	2.113332
H	-6.658754	1.550797	2.010321
H	-3.949111	1.004072	-2.635445
H	-3.045484	0.478648	-4.061213
H	1.658698	2.773667	-4.370631
H	0.140315	3.631125	-4.014973

H	2.355894	-0.997935	-2.146663
H	1.020233	-1.820593	-1.348902
H	2.229941	-3.404713	-2.835088
H	0.568630	-3.140084	-3.424859
H	1.933712	-2.330266	-4.241387
H	-3.822072	2.794201	-4.348713
H	-2.981742	3.305076	-2.850834
H	-2.035783	2.779478	-4.262469
H	2.045317	5.125846	-3.768440
H	1.241140	4.896955	-2.186728
H	2.789659	4.070428	-2.530040

5.2 Acetone (3o)

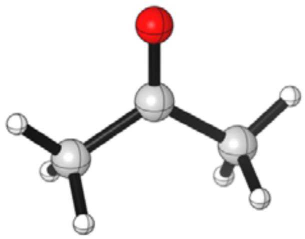

C	1.274692	-0.180094	2.058339
C	0.006395	-0.005077	1.245844
C	-1.277322	0.160314	2.035841
O	0.018038	0.002181	0.034788
H	1.149945	-0.962026	2.815510
H	2.107293	-0.428140	1.398141
H	1.501620	0.750993	2.591501
H	-1.167230	0.933023	2.804659
H	-2.097063	0.416455	1.362766
H	-1.514415	-0.777113	2.553228

5.3 Triethoxysilane (4a)

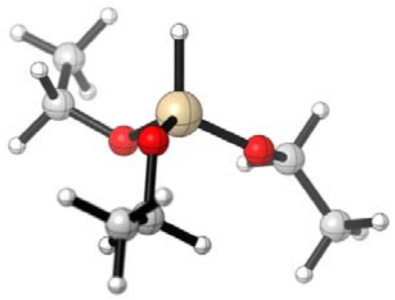

C	1.863680	0.000020	3.481743
C	3.053391	-0.487545	2.671877
O	2.739214	-1.701827	2.007982
Si	2.365361	-1.767664	0.402031
O	1.564050	-3.180525	0.175913
C	0.626388	-3.755539	1.074126
C	0.685087	-5.267091	0.953073
H	1.604863	-0.560434	0.015863
O	3.682417	-1.765679	-0.574082
C	4.666593	-2.793097	-0.544440
C	5.731804	-2.482395	-1.577896
H	3.909104	-0.684372	3.326703
H	3.367023	0.285710	1.955920
H	0.846885	-3.445991	2.102850
H	-0.377797	-3.392287	0.818770
H	5.109703	-2.849020	0.458885
H	4.193893	-3.760157	-0.755925
H	-0.052873	-5.732080	1.615464
H	0.474894	-5.575334	-0.075383
H	1.679423	-5.632343	1.227278
H	6.504097	-3.258805	-1.575727
H	5.289097	-2.433477	-2.577078
H	6.203705	-1.518904	-1.363273
H	2.119982	0.905999	4.041367
H	1.018630	0.227049	2.823267
H	1.548331	-0.771269	4.190898

5.4 Silyl Ether 8oa

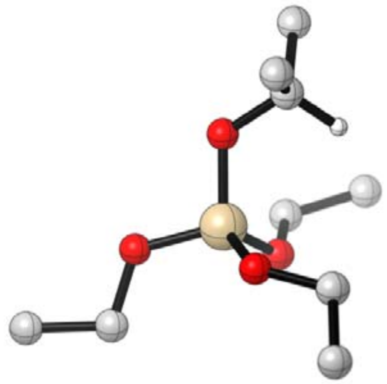

C	0.131313	-0.235600	0.113836
H	-0.107137	-0.781265	1.041523
O	1.507446	-0.422557	-0.214361
C	-0.733010	-0.784265	-1.013598
C	-0.087724	1.249087	0.359710
H	-0.505231	-0.256303	-1.945301
H	-0.528324	-1.848658	-1.163351
H	-1.796977	-0.656117	-0.787026
H	0.171967	1.816946	-0.539530
H	-1.133560	1.450843	0.613685
H	0.545244	1.598502	1.181349
Si	2.341701	-1.727121	0.319473
O	3.698579	-1.742600	-0.588607
O	2.627229	-1.601979	1.937515
O	1.553287	-3.150641	0.119672
C	2.897139	-0.370115	2.590792
C	0.672742	-3.763000	1.048624
C	4.679201	-2.763446	-0.455442
C	1.828415	-0.092432	3.633702
H	3.881956	-0.448617	3.065093
H	2.939078	0.453381	1.866628
C	0.771799	-5.270194	0.904988
H	0.924723	-3.454869	2.070071
H	-0.352580	-3.428382	0.837663
C	5.694191	-2.617133	-1.572524
H	5.169955	-2.676966	0.523279
H	4.197312	-3.748478	-0.503106

H	0.080755	-5.768221	1.593415
H	0.525948	-5.572394	-0.117281
H	1.789065	-5.605600	1.128707
H	6.461384	-3.394590	-1.492970
H	5.202515	-2.707211	-2.545375
H	6.180878	-1.638681	-1.521742
H	2.052590	0.830745	4.178890
H	0.847514	0.016325	3.158810
H	1.773180	-0.916682	4.351286

5.5 Tetrahydrofuran

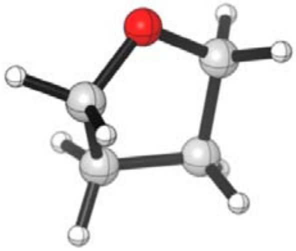

C	0.148690	-0.304677	10.235120
O	-0.531908	0.753841	9.569670
C	-0.656101	1.851565	10.454267
C	-0.754715	1.231901	11.845041
C	0.253212	0.082807	11.723997
H	0.225113	2.510888	10.386886
H	-1.538705	2.424731	10.157696
H	-1.765467	0.845220	12.011234
H	-0.515053	1.935332	12.647274
H	0.031611	-0.755180	12.389950
H	1.261503	0.439233	11.959235
H	-0.433911	-1.220903	10.086796
H	1.138422	-0.454722	9.784550

5.6 Benzene

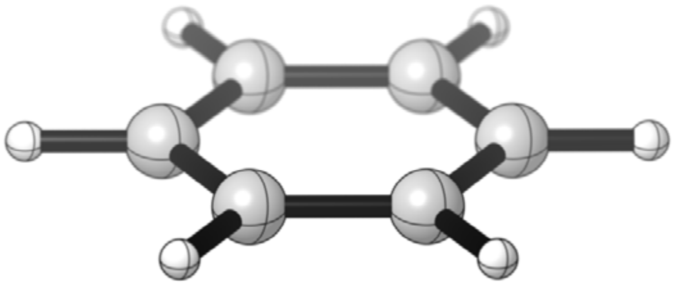

C	-1.498017	2.748940	2.920399
C	-0.517801	1.759962	2.954130
C	0.801580	2.075912	2.638759
C	1.140564	3.381164	2.289622
C	0.160329	4.369941	2.255701
C	-1.159073	4.053921	2.571011
H	2.169575	3.627407	2.043678
H	0.424586	5.387751	1.983164
H	-1.923777	4.824789	2.544491
H	-2.527059	2.502746	3.166228
H	-0.782401	0.742062	3.226236
H	1.566177	1.304908	2.664720

5.7 Transition State 9a‡

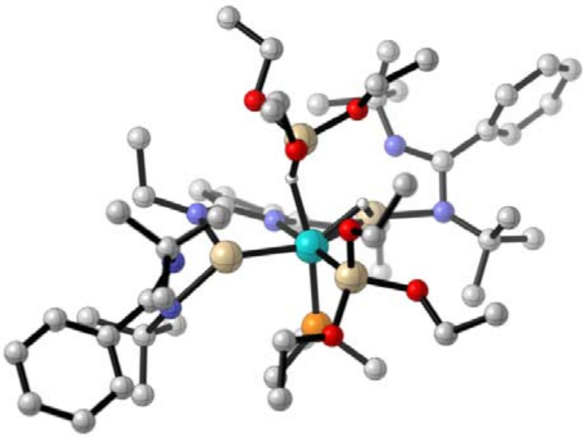

C	7.096135	-0.429482	0.964932
C	6.148857	-0.223079	-0.054233
C	6.591801	0.064400	-1.359750

C	7.960599	0.178793	-1.633256
C	8.903184	-0.015425	-0.608791
C	8.467832	-0.327432	0.689649
C	4.681179	-0.283614	0.235431
N	3.960527	0.671588	0.801627
C	4.442415	2.056800	1.097504
C	5.420268	2.658673	0.048029
N	3.892057	-1.323535	-0.141236
C	4.390441	-2.718436	-0.424415
C	3.147916	-3.618793	-0.545190
Si	2.152888	-0.540733	0.303245
N	1.798069	-1.745960	1.687290
C	2.777816	-1.980930	2.759456
C	2.284452	-1.528236	4.140303
C	0.709957	-2.574728	1.557999
N	-0.215683	-2.238928	0.585568
C	-1.188196	-3.168061	0.275062
C	-1.183099	-4.473528	0.812697
C	-0.312029	-4.744618	1.872730
C	0.597509	-3.779920	2.300236
N	-2.195737	-2.697903	-0.541857
Si	-2.152500	-0.883451	-0.603054
N	-3.795694	-0.405015	-1.393541
C	-4.479359	-0.399953	-0.211346
C	-5.925582	-0.078546	-0.060490
C	-6.914364	-1.016879	-0.416138
C	-8.270281	-0.715281	-0.238177
C	-8.652017	0.530760	0.289041
C	-7.669547	1.470561	0.640893
C	-6.310932	1.165643	0.472871
Fe	-0.009533	-0.390451	-0.508865
P	0.517043	-1.465889	-2.417727
C	0.250193	-3.328945	-2.517393
Si	0.240060	1.525100	-1.733846
O	1.583469	1.741034	-2.803564
C	2.897081	1.999654	-2.341348
C	3.831302	2.183732	-3.541859

O	-0.998883	1.920516	-2.852187
C	-0.874127	2.464639	-4.166353
C	-1.808319	3.666834	-4.342030
O	0.230907	2.878286	-0.687337
C	0.329568	4.227229	-1.131236
C	-0.720870	5.078537	-0.409621
C	-3.365916	-3.536567	-0.813151
C	-3.150108	-4.525266	-1.972874
N	-3.661892	-0.737590	0.763104
C	-4.056491	-1.219043	2.127730
C	-5.201384	-2.262380	2.092900
C	-2.817608	-1.894267	2.747848
C	-4.506884	-0.041146	3.015566
C	-4.231058	0.317566	-2.630598
C	-5.594691	-0.190781	-3.164079
C	-3.180261	0.013120	-3.698381
C	-4.304748	1.835990	-2.366136
C	2.289449	-1.317838	-3.043966
C	-0.195988	-1.113229	-4.126683
C	5.306004	-3.272372	0.695327
C	5.185730	-2.821542	-1.757378
C	5.122237	2.048345	2.487601
C	3.214402	2.991490	1.159044
O	0.359864	2.695051	1.740291
C	0.464065	3.372948	2.970673
C	1.224569	4.607958	2.783108
O	-2.099624	1.722313	1.965952
C	-2.289556	3.093739	2.210029
C	-3.716318	3.403793	2.173494
O	-0.159496	0.705306	3.461226
C	-1.134368	1.020220	4.422174
C	-0.776776	0.407474	5.698790
H	-0.286737	0.012362	1.268582
H	-5.704644	0.155118	-4.204752
H	-3.412950	0.567895	-4.620353
H	-1.446063	0.556891	-0.216124
H	1.265658	-3.984428	3.131249

H	-0.348083	-5.715014	2.373426
H	-1.905965	-5.213150	0.478725
H	3.688761	-1.429120	2.494007
H	3.051890	-3.050367	2.798184
H	2.091258	-0.449078	4.149902
H	3.045378	-1.762667	4.905042
H	1.343041	-2.021880	4.415161
H	-4.201085	-2.869542	-1.064703
H	-3.652574	-4.088479	0.099525
H	-3.005210	-3.979378	-2.918222
H	-4.029896	-5.182167	-2.081417
H	-2.262553	-5.151445	-1.798422
H	2.645216	-3.757611	0.418810
H	2.594006	2.912311	0.257643
H	3.568858	4.029401	1.257804
H	2.589787	2.759582	2.023514
H	5.457855	3.750467	0.189886
H	5.066407	2.459638	-0.972642
H	4.446345	1.603226	3.235096
H	5.353163	3.082154	2.795608
H	6.060910	1.476006	2.463692
H	-3.744083	0.739055	3.060093
H	-4.660651	-0.414699	4.038649
H	-5.455453	0.379460	2.654362
H	-6.177544	-1.810061	1.878488
H	-5.258470	-2.734476	3.086913
H	-4.997044	-3.047519	1.351519
H	-2.624446	-2.867753	2.279430
H	-3.002058	-2.058996	3.820680
H	-1.928583	-1.263344	2.657046
H	-5.085622	2.063952	-1.626652
H	-4.562328	2.357740	-3.302411
H	-3.332920	2.205832	-2.014207
H	-5.626075	-1.292092	-3.162174
H	-6.447635	0.192766	-2.592404
H	-2.193144	0.319218	-3.343240
H	-3.188349	-1.064052	-3.927580

H	-5.542882	1.886729	0.753867
H	-6.612473	-1.981676	-0.824812
H	-0.263271	-0.032988	-4.297101
H	-1.185828	-1.567633	-4.245556
H	0.479362	-1.553056	-4.879046
H	-0.802881	-3.500236	-2.769811
H	0.455484	-3.839356	-1.569515
H	0.888433	-3.756440	-3.308163
H	4.654604	-2.348901	-2.592581
H	5.317981	-3.889344	-1.997601
H	6.183304	-2.374361	-1.671552
H	2.445069	-3.176533	-1.253001
H	6.307374	-2.824967	0.656491
H	4.877393	-3.102832	1.689276
H	6.442944	2.275528	0.148068
H	5.857614	0.208879	-2.151124
H	8.292696	0.415783	-2.646256
H	9.970533	0.071923	-0.822284
H	9.194624	-0.490502	1.488154
H	6.753559	-0.674473	1.971201
H	2.539337	-2.187500	-3.674735
H	3.012455	-1.228775	-2.230710
H	2.333362	-0.402887	-3.646786
H	-7.958974	2.440284	1.050850
H	-9.709371	0.766660	0.425205
H	-9.028936	-1.451653	-0.511021
H	3.447396	-4.607195	-0.926067
H	5.417511	-4.359395	0.548680
H	-1.144882	1.678695	-4.898877
H	0.167771	2.760590	-4.366466
H	1.342714	4.606288	-0.891046
H	0.201165	4.305389	-2.225493
H	2.909310	2.911180	-1.716184
H	3.264101	1.168416	-1.710556
H	4.856372	2.414221	-3.204779
H	3.860092	1.268995	-4.155277
H	3.476275	3.012150	-4.175984

H	-1.735937	4.054099	-5.373379
H	-2.851602	3.382289	-4.144472
H	-1.533714	4.472302	-3.642576
H	-0.601068	6.147975	-0.656249
H	-1.735304	4.755984	-0.694515
H	-0.611420	4.956751	0.680491
H	-1.194788	2.103652	4.540576
H	-2.103818	0.638300	4.101402
H	-1.882707	3.359320	3.186247
H	-1.777941	3.669871	1.440208
H	-0.528644	3.603896	3.357169
H	0.984182	2.742559	3.694685
H	-1.533367	0.651268	6.446071
H	-0.720516	-0.675643	5.581751
H	0.191761	0.784611	6.030298
H	-3.864754	4.467868	2.362740
H	-4.119857	3.152809	1.191564
H	-4.238964	2.825941	2.936430
H	1.304298	5.136659	3.734123
H	2.226136	4.373930	2.417446
H	0.717894	5.245745	2.057473
Si	-0.494112	1.293582	1.978123

5.8 Transition State 10oa‡

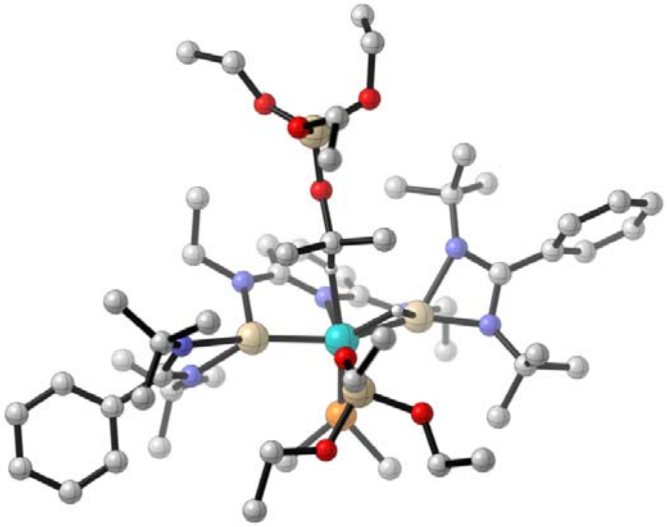

C	-6.738987	-1.357006	0.088629
C	-6.227574	-0.087057	-0.241705
C	-7.120846	0.962220	-0.522114
C	-8.506596	0.750859	-0.458676
C	-9.010324	-0.512462	-0.108491
C	-8.121564	-1.566596	0.164383
C	-4.744760	0.132408	-0.249102
N	-4.056330	0.724896	0.712145
C	-4.611384	1.124690	2.046263
C	-5.659529	0.148722	2.654430
Si	-2.185011	-0.009503	-0.459534
N	-3.947991	-0.331525	-1.252267
C	-4.430229	-0.617163	-2.654293
C	-3.179800	-0.865033	-3.516309
Fe	-0.111024	-1.000386	-0.264184
Si	-0.510646	-2.110346	1.702501
O	-1.915380	-3.105891	1.900999
C	-3.209099	-2.549058	2.067318
C	-4.227224	-3.676495	2.268320
N	0.223189	-0.045017	-2.164813
C	-0.601950	1.006519	-2.530205
C	-0.406971	1.680795	-3.765503
C	0.459835	1.126043	-4.705058

C	1.222595	-0.003197	-4.388358
C	1.168577	-0.487105	-3.063487
N	-1.665623	1.289981	-1.701196
C	-2.517719	2.452902	-1.992310
C	-1.870968	3.783747	-1.588192
N	2.088866	-1.374148	-2.541873
Si	2.035469	-1.342407	-0.724786
N	3.679893	-0.126223	-0.644670
C	4.232278	1.149148	-1.212061
C	5.388598	0.918306	-2.217392
P	-0.791765	-2.919249	-1.234170
C	-0.501336	-3.156340	-3.080681
C	-2.616639	-3.372199	-1.111175
C	-0.253993	-4.663463	-0.759707
C	3.236487	-1.792092	-3.350957
C	2.925113	-2.970317	-4.290862
N	3.589799	-2.262871	-0.185494
C	4.390601	-1.157747	-0.237555
C	5.842314	-1.137611	0.095278
C	6.270726	-0.573273	1.312296
C	7.636484	-0.528171	1.627856
C	8.585623	-1.033930	0.724671
C	8.162408	-1.591260	-0.494662
C	6.798164	-1.646542	-0.806115
C	3.891703	-3.492303	0.614116
C	5.192737	-4.194724	0.149313
C	2.734746	-4.458621	0.365320
C	3.990594	-3.147719	2.114872
C	3.086859	1.845819	-1.970654
C	4.748952	2.070122	-0.087778
O	0.661723	-3.245075	2.223216
C	0.452420	-4.522552	2.827223
C	1.309740	-4.667570	4.089360
O	-0.662110	-1.033952	3.030318
C	-0.870017	-1.448140	4.376414
C	0.083431	-0.675722	5.294830
C	-5.225808	0.562553	-3.267081

C	-5.338487	-1.876651	-2.753184
C	-5.233540	2.536002	1.912346
C	-3.446650	1.220568	3.056415
H	0.358492	0.813087	-0.091722
H	5.192598	-5.218373	0.557812
H	2.866694	-5.365223	0.975697
H	1.383774	-0.793818	0.644390
H	-0.980937	2.569896	-4.008369
H	0.549948	1.581911	-5.693811
H	1.914638	-0.433323	-5.107663
H	-3.451657	2.315550	-1.431719
H	-2.786372	2.472570	-3.063455
H	-1.685666	3.811197	-0.507912
H	-2.536223	4.622003	-1.859126
H	-0.900118	3.929361	-2.079220
H	4.037781	-2.088922	-2.661323
H	3.612924	-0.936136	-3.938189
H	2.688488	-3.873145	-3.706600
H	3.798392	-3.188042	-4.929020
H	2.064506	-2.743759	-4.937624
H	-2.596922	0.050006	-3.668557
H	-2.851372	0.298090	3.076936
H	-3.859780	1.404108	4.061156
H	-2.790430	2.059203	2.808973
H	-5.755572	0.363484	3.730775
H	-5.329878	-0.893810	2.542611
H	-4.501696	3.229558	1.468377
H	-5.514684	2.914886	2.909563
H	-6.135529	2.513457	1.285217
H	3.978813	2.261057	0.663714
H	5.017562	3.041000	-0.527897
H	5.643763	1.643347	0.386631
H	6.326453	0.635078	-1.724136
H	5.562011	1.864402	-2.754789
H	5.122136	0.148695	-2.954819
H	2.869810	1.327380	-2.913387
H	3.391737	2.876952	-2.206244

H	2.178487	1.900982	-1.365048
H	4.844963	-2.483208	2.305938
H	4.146075	-4.073714	2.692640
H	3.061383	-2.673111	2.455028
H	5.226073	-4.262534	-0.949915
H	6.099864	-3.692561	0.504415
H	1.789772	-3.981745	0.638244
H	2.721730	-4.751726	-0.695866
H	5.529849	-0.171571	2.004351
H	6.465187	-2.076953	-1.750995
H	-0.226555	-4.772161	0.331340
H	0.726263	-4.908610	-1.182029
H	-0.991544	-5.372402	-1.170257
H	0.526463	-3.513811	-3.211425
H	-0.607690	-2.226025	-3.649998
H	-1.201636	-3.909960	-3.476337
H	-4.902725	-2.744426	-2.243528
H	-5.456444	-2.131771	-3.819276
H	-6.338365	-1.690569	-2.344017
H	-2.548746	-1.613882	-3.035658
H	-6.239054	0.626382	-2.850563
H	-4.714598	1.519048	-3.111119
H	-6.654544	0.255239	2.206515
H	-6.046669	-2.170455	0.299818
H	-8.506149	-2.552377	0.434690
H	-10.088592	-0.675521	-0.051453
H	-9.190697	1.572314	-0.681655
H	-6.726241	1.942251	-0.792914
H	-2.896729	-4.030762	-1.950464
H	-3.263722	-2.493209	-1.096009
H	-2.740874	-3.912124	-0.166066
H	7.958117	-0.093526	2.576420
H	9.649249	-0.993722	0.968080
H	8.895553	-1.982886	-1.202753
H	-3.484338	-1.248804	-4.502043
H	-5.319347	0.393807	-4.352653
H	0.732508	-5.304723	2.094744

H	-0.611444	-4.664804	3.073855
H	-1.919126	-1.230840	4.657793
H	-0.717528	-2.535510	4.493871
H	-3.224876	-1.872946	2.941777
H	-3.493487	-1.941561	1.188162
H	-5.237987	-3.264033	2.428776
H	-4.253959	-4.339265	1.388718
H	-3.952706	-4.283020	3.146557
H	1.173060	-5.672743	4.524989
H	2.374869	-4.525275	3.857466
H	1.018551	-3.915439	4.839615
H	-0.124445	-0.894536	6.356749
H	1.127887	-0.946616	5.072271
H	-0.036181	0.407465	5.127560
C	0.513390	1.512988	0.837410
O	0.917098	2.785284	0.155185
C	-0.761810	1.800194	1.586817
C	1.634917	1.132464	1.806896
Si	1.269803	4.274204	0.713920
H	-0.587384	2.607189	2.312051
H	-1.563827	2.128123	0.909360
H	-1.063953	0.900917	2.117513
H	1.833594	2.004989	2.452592
H	1.325769	0.300369	2.450646
H	2.553707	0.859650	1.274989
O	0.548305	5.371749	-0.307320
O	0.722706	4.644095	2.249401
O	2.927304	4.459300	0.703484
C	0.176067	6.694283	0.106176
C	3.548340	5.739676	0.877213
C	1.512577	4.553508	3.444207
C	-0.627801	7.351143	-1.015423
H	-0.415338	6.645653	1.036049
H	1.083048	7.292759	0.315541
C	0.751121	3.789626	4.529922
H	2.480828	4.065003	3.236929
H	1.728492	5.581512	3.788363

C	3.832835	6.397607	-0.476426
H	2.923943	6.409472	1.498341
H	4.489965	5.564939	1.425360
H	4.356380	7.358653	-0.335911
H	4.464335	5.737198	-1.091216
H	2.891753	6.575595	-1.016605
H	1.321484	3.808366	5.473854
H	-0.232825	4.253205	4.701883
H	0.595912	2.741669	4.233769
H	-0.911076	8.377527	-0.728405
H	-0.029777	7.394517	-1.939524
H	-1.540697	6.771922	-1.219979

5.9 Transition State 13a‡

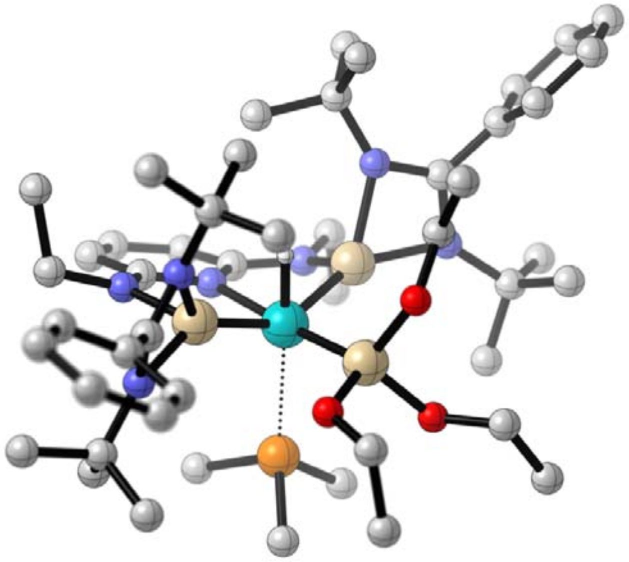

C	-1.408165	3.190742	0.483471
N	-0.204057	2.555103	0.245762
C	0.844488	3.289843	-0.260515
C	0.751075	4.695657	-0.417166
C	-0.426784	5.337769	-0.040257
C	-1.524384	4.599170	0.411350
Fe	0.043763	0.518826	0.696527
Si	0.296858	-1.667728	1.044809
O	-0.149950	-2.801952	-0.189135

C	-1.243651	-2.614823	-1.062187
C	-1.119563	-3.574781	-2.250831
N	1.981870	2.575765	-0.592750
C	3.084007	3.245603	-1.290677
C	2.769032	3.520935	-2.772583
N	-2.483597	2.350165	0.725686
C	-3.838488	2.900695	0.805831
C	-4.176334	3.421654	2.215026
Si	-2.074099	0.605394	0.359093
N	-3.771329	-0.267318	0.546378
C	-4.494247	-1.034734	1.590922
C	-5.943710	-0.516818	1.734512
N	-2.998735	0.460992	-1.339624
C	-2.858289	1.012657	-2.707076
C	-4.132310	1.780139	-3.129374
C	-3.988258	-0.241576	-0.787534
C	-5.110390	-0.908386	-1.508972
C	-4.898470	-2.155689	-2.126975
C	-5.943087	-2.783714	-2.820807
C	-7.201650	-2.167110	-2.909244
C	-7.414775	-0.920909	-2.296804
C	-6.374316	-0.295002	-1.595023
C	-1.681665	2.011237	-2.665978
C	-2.556170	-0.106333	-3.730632
C	-3.738814	-0.779881	2.902293
C	-4.495709	-2.553179	1.308988
P	0.317480	1.107866	3.414532
C	0.797359	2.894521	3.754600
Si	1.984116	0.817516	-0.139109
N	3.851477	0.585402	0.236219
C	4.803394	0.928547	1.311557
C	4.112759	2.017744	2.156769
C	1.523920	0.143205	4.469752
C	-1.104950	1.042412	4.632344
N	2.899634	-0.018225	-1.613582
C	4.036359	-0.106481	-0.894014
C	5.260573	-0.887261	-1.227872

C	6.253475	-0.362367	-2.076218
C	7.414548	-1.102532	-2.341198
C	7.588386	-2.371264	-1.762947
C	6.598325	-2.896510	-0.916891
C	5.438983	-2.155532	-0.645002
C	2.536925	-0.813049	-2.821128
C	2.475177	-2.321669	-2.498184
C	3.534193	-0.546729	-3.974561
C	1.144876	-0.346737	-3.284806
C	6.121236	1.510587	0.748669
C	5.109300	-0.311961	2.184747
O	1.947196	-2.162650	1.229030
C	2.334490	-3.477061	1.605277
C	2.498486	-3.586245	3.128821
O	-0.552168	-2.283468	2.435656
C	-0.988753	-3.637580	2.560057
C	-1.648525	-3.838693	3.927711
H	-6.408715	-0.986529	2.616501
H	-4.230506	-1.326703	3.722356
H	0.074637	0.118764	-0.772491
H	1.587353	5.266676	-0.812372
H	-0.501234	6.424861	-0.122080
H	-2.456762	5.094751	0.667492
H	3.959591	2.586200	-1.218038
H	3.355319	4.182330	-0.770332
H	2.584422	2.567306	-3.289441
H	3.613756	4.033225	-3.264471
H	1.870933	4.149767	-2.870055
H	-4.533107	2.088435	0.547949
H	-3.981673	3.698287	0.052542
H	-4.119432	2.595138	2.941305
H	-5.194171	3.847008	2.244390
H	-3.461035	4.198105	2.526837
H	3.906479	2.908140	1.543751
H	0.387204	-0.606886	-2.538330
H	0.896009	-0.849176	-4.232487
H	1.122608	0.741473	-3.445188

H	2.094067	-2.866064	-3.379027
H	1.805725	-2.502854	-1.646104
H	3.660446	0.536888	-4.132135
H	3.133518	-0.989007	-4.901252
H	4.515459	-0.997484	-3.781457
H	-1.656314	-0.663922	-3.441142
H	-2.386917	0.342235	-4.723306
H	-3.400189	-0.805630	-3.807060
H	-4.982621	1.104960	-3.294536
H	-3.931121	2.318124	-4.069891
H	-4.401521	2.517863	-2.356609
H	-1.936521	2.887834	-2.053718
H	-1.462826	2.351255	-3.690468
H	-0.787297	1.542640	-2.238488
H	-5.035649	-2.795829	0.383483
H	-4.992075	-3.073089	2.144696
H	-3.463517	-2.923503	1.237485
H	-5.946542	0.575241	1.879886
H	-6.552119	-0.763599	0.854173
H	-2.701438	-1.126314	2.809364
H	-3.741748	0.292075	3.148650
H	-3.919047	-2.629128	-2.052132
H	-6.532329	0.674450	-1.120480
H	-1.424271	0.001570	4.761062
H	-1.944677	1.639464	4.248237
H	-0.789701	1.447636	5.609721
H	-0.074260	3.527434	3.527267
H	1.619596	3.213014	3.101446
H	1.083164	3.038653	4.810790
H	4.179724	-0.831594	2.453608
H	5.636224	-0.001885	3.102489
H	5.751779	-1.017284	1.639497
H	3.163482	1.647657	2.557468
H	6.717812	0.749180	0.229883
H	5.907067	2.336391	0.051471
H	3.474147	-2.714541	-2.264699
H	4.660469	-2.547757	0.007563

H	6.726611	-3.883178	-0.467040
H	8.492258	-2.947366	-1.971701
H	8.182543	-0.689401	-2.998418
H	6.110950	0.622902	-2.522630
H	1.640961	0.609180	5.463590
H	2.500942	0.053789	3.984551
H	1.112486	-0.870168	4.581461
H	-5.771742	-3.753147	-3.293128
H	-8.013334	-2.655104	-3.452594
H	-8.390745	-0.435972	-2.364192
H	4.763734	2.303614	2.997378
H	6.721160	1.907186	1.583738
H	3.306040	-3.700566	1.123619
H	1.622875	-4.232686	1.225169
H	-1.706828	-3.882386	1.754543
H	-0.147611	-4.344432	2.456554
H	-2.205151	-2.798842	-0.540977
H	-1.276829	-1.572250	-1.421355
H	-1.968631	-3.446862	-2.944440
H	-0.187232	-3.378251	-2.801659
H	-1.100621	-4.619741	-1.901637
H	2.807199	-4.606769	3.415855
H	3.264348	-2.873854	3.477124
H	1.552341	-3.336650	3.630554
H	-1.995754	-4.880001	4.044103
H	-0.930075	-3.616127	4.733097
H	-2.511467	-3.165273	4.040181

5.10 Intermediate *cis*-14a

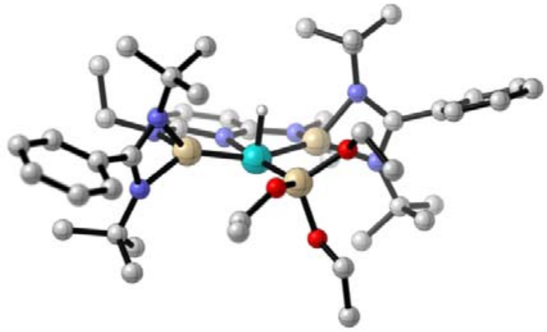

C	-4.959676	-2.107994	-1.269533
C	-5.255469	-0.787792	-0.879638
C	-6.573973	-0.305068	-0.959465
C	-7.595818	-1.140784	-1.435315
C	-7.302628	-2.457346	-1.827498
C	-5.985072	-2.939462	-1.740757
C	-4.146769	0.081155	-0.406222
N	-3.260894	0.728699	-1.177748
C	-3.095408	0.820573	-2.640954
C	-2.149190	2.018650	-2.883772
Si	-2.133957	1.032882	0.295678
N	-3.781949	0.305999	0.872068
C	-4.293278	-0.287817	2.128605
C	-3.309584	0.145639	3.236247
Fe	-0.037443	0.638077	0.486048
N	0.045055	2.743072	0.472877
C	1.242397	3.407500	0.270365
C	1.326030	4.820517	0.319759
C	0.164923	5.559552	0.545692
C	-1.065946	4.915337	0.676073
C	-1.107777	3.503904	0.595996
N	2.347553	2.611745	0.013919
C	3.628212	3.231198	-0.341580
C	3.651920	3.760123	-1.787457
N	-2.302217	2.800974	0.590822
C	-3.583103	3.515469	0.557997
C	-4.043549	3.994018	1.947259
Si	2.071781	0.820628	0.092849

N	3.736639	0.304873	0.885381
C	4.360000	0.429027	2.217882
C	5.766207	1.064194	2.133634
C	4.206678	-0.146884	-0.284217
N	3.289127	0.163697	-1.225795
C	3.137855	-0.407629	-2.590189
C	4.335831	-0.019511	-3.486833
C	5.497499	-0.859179	-0.491867
C	6.601898	-0.162407	-1.020530
C	7.832412	-0.814109	-1.183728
C	7.965674	-2.166965	-0.827942
C	6.864299	-2.864836	-0.306453
C	5.634208	-2.213197	-0.133418
C	1.853706	0.203641	-3.187155
C	2.990116	-1.944931	-2.522292
C	4.442983	-0.951906	2.908268
C	3.432598	1.351681	3.040203
Si	-0.347308	-1.569794	0.545651
O	-1.571276	-2.194447	-0.503445
C	-1.446044	-3.332053	-1.355596
O	0.878263	-2.748975	0.191345
C	2.009819	-2.870936	1.031245
O	-0.821621	-1.995889	2.150369
C	-1.025260	-3.331633	2.603937
C	-4.320577	-1.831194	2.061765
C	-5.702072	0.270567	2.427744
C	-2.445047	-0.474141	-3.177006
C	-4.440901	1.085366	-3.348996
H	-6.048849	-0.105840	3.404042
H	-3.685637	-0.197227	4.213075
H	-0.131441	0.549337	-0.990858
H	2.276494	5.326939	0.175244
H	0.217355	6.649501	0.602051
H	-1.977893	5.488546	0.819186
H	4.405182	2.464002	-0.224848
H	3.880597	4.035586	0.373401
H	3.463173	2.928138	-2.482737

H	4.633206	4.205944	-2.025020
H	2.872926	4.522127	-1.939260
H	-4.329483	2.819060	0.147370
H	-3.520645	4.368804	-0.142223
H	-4.182755	3.129574	2.613556
H	-5.000155	4.538982	1.873346
H	-3.295028	4.659030	2.403758
H	3.359616	2.345902	2.575537
H	0.974038	-0.090237	-2.598854
H	1.730718	-0.153141	-4.221707
H	1.912730	1.303586	-3.195683
H	2.756459	-2.337536	-3.525895
H	2.180696	-2.221567	-1.830436
H	4.487711	1.071607	-3.475687
H	4.123577	-0.331907	-4.522049
H	5.262216	-0.512358	-3.164012
H	-1.553399	-0.716013	-2.583356
H	-2.168520	-0.342458	-4.236505
H	-3.149912	-1.314772	-3.099317
H	-5.118096	0.224120	-3.272948
H	-4.248032	1.280910	-4.416038
H	-4.936741	1.969146	-2.915572
H	-2.597929	2.948325	-2.499868
H	-1.967871	2.128610	-3.964628
H	-1.186946	1.856454	-2.374603
H	-5.103589	-2.192088	1.380897
H	-4.525232	-2.233750	3.067526
H	-3.343349	-2.195457	1.721738
H	-5.680441	1.371395	2.463054
H	-6.421476	-0.049286	1.659560
H	-2.323097	-0.305532	3.055410
H	-3.212286	1.242676	3.261760
H	-3.936138	-2.464100	-1.168755
H	-6.790755	0.722422	-0.662082
H	3.444736	-1.409714	2.962851
H	4.834792	-0.829022	3.931362
H	5.115796	-1.624842	2.357642

H	2.419723	0.921509	3.097793
H	6.477591	0.404564	1.618348
H	5.721244	2.027233	1.601546
H	3.927643	-2.410853	-2.183330
H	4.775635	-2.750494	0.265317
H	6.959569	-3.917904	-0.033951
H	8.923646	-2.674455	-0.958185
H	8.685471	-0.266725	-1.589744
H	6.489043	0.886782	-1.297846
H	-5.755404	-3.965401	-2.036401
H	-8.099289	-3.106514	-2.196981
H	-8.618380	-0.763319	-1.500970
H	3.830449	1.457611	4.061217
H	6.140474	1.247112	3.153655
H	2.725522	-2.055079	0.828884
H	1.727046	-2.781218	2.098947
C	2.672319	-4.231802	0.789673
C	-1.277260	-3.315730	4.114447
H	-1.891238	-3.782723	2.084483
H	-0.145967	-3.961937	2.370379
H	-2.141789	-3.180247	-2.202125
H	-0.426910	-3.400116	-1.771673
C	-1.788644	-4.640544	-0.633023
H	-1.442083	-4.339462	4.492667
H	-0.414200	-2.877506	4.641627
H	-2.165784	-2.706435	4.343820
H	3.558412	-4.356614	1.436962
H	1.961886	-5.044605	1.009627
H	2.983778	-4.324702	-0.263477
H	-1.769047	-5.490077	-1.338946
H	-1.056914	-4.825853	0.165923
H	-2.793512	-4.580982	-0.181667

5.11 Transition State 15a‡

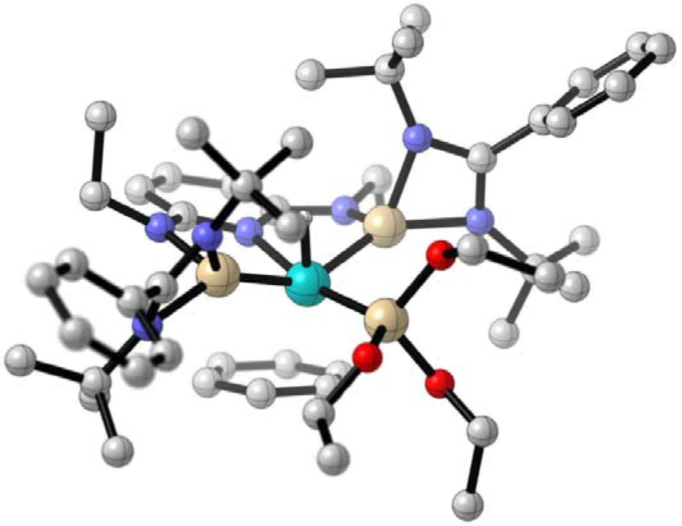

C	-0.527473	1.861621	2.868683
C	0.775473	2.233001	2.522584
C	1.054831	3.577897	2.196099
C	0.055253	4.525048	2.204899
C	-1.247789	4.163771	2.582904
C	-1.526768	2.856414	2.917030
Fe	-0.017688	0.790118	0.546070
Si	-2.108398	0.762741	0.044985
N	-2.897341	0.013512	-1.486726
C	-2.721066	0.068260	-2.945292
C	-2.348778	-1.308284	-3.514767
N	-0.165516	2.548601	-0.509389
C	0.913423	3.022479	-1.185282
C	0.894510	4.301474	-1.770616
C	-0.270847	5.042811	-1.700655
C	-1.417947	4.511704	-1.134193
C	-1.352734	3.217101	-0.594336
N	2.007046	2.186081	-1.279381
Si	1.962126	0.699853	-0.261671
N	2.826391	-0.664328	-1.276150
C	3.990233	-0.477710	-0.643561
C	5.207174	-1.335143	-0.739654
C	5.436826	-2.317536	0.224094
C	6.580306	-3.106603	0.160338

C	7.504287	-2.915181	-0.863426
C	7.279285	-1.934593	-1.826576
C	6.133369	-1.147440	-1.766410
N	-2.470860	2.506513	-0.219375
C	-3.794251	3.047203	-0.473249
C	-4.285258	4.001072	0.614783
Si	-0.162932	-1.282614	1.383123
O	1.178588	-2.323696	1.692872
C	2.264948	-1.855442	2.438889
C	2.988832	-3.028370	3.082357
O	-0.941410	-1.324441	2.918474
C	-0.890958	-2.356646	3.867883
C	-0.495022	-1.796045	5.226981
O	-1.030627	-2.378826	0.381464
C	-0.802995	-3.759414	0.225858
C	-1.730173	-4.611352	1.085300
C	3.039175	2.474214	-2.262808
C	2.571377	2.248166	-3.700576
N	3.866918	0.589742	0.132269
C	4.887883	1.297424	0.922135
C	5.325679	0.480222	2.148088
C	4.225374	2.583201	1.416684
C	6.112540	1.674476	0.073100
C	2.443813	-1.835645	-2.099905
C	0.979349	-1.674007	-2.506670
C	2.577772	-3.148301	-1.310787
C	3.289004	-1.878882	-3.383219
N	-3.757964	-0.052543	0.481516
C	-3.846964	-0.554997	-0.757507
C	-4.736057	-1.653995	-1.217193
C	-4.177449	-2.931649	-1.288970
C	-4.953680	-4.004001	-1.712331
C	-6.283343	-3.799970	-2.077226
C	-6.835936	-2.523223	-2.016170
C	-6.064122	-1.447652	-1.583544
C	-4.440755	-0.515255	1.707141
C	-4.150098	-1.990036	2.010821

C	-5.951648	-0.264631	1.591937
C	-3.891889	0.337484	2.849038
C	-4.004177	0.590788	-3.610249
C	-1.592206	1.071225	-3.223656
H	-6.426866	-0.435992	2.563931
H	-4.379727	0.060295	3.789218
H	0.101839	0.104659	-0.798753
H	1.769695	4.697936	-2.267718
H	-0.297354	6.041629	-2.128392
H	-2.344338	5.069366	-1.138383
H	3.875811	1.803077	-2.053222
H	3.434327	3.492263	-2.136513
H	2.228071	1.214773	-3.814657
H	3.387599	2.425229	-4.410252
H	1.739504	2.910063	-3.959554
H	-4.483160	2.198155	-0.532574
H	-3.826377	3.541021	-1.456168
H	-4.308112	3.488461	1.581532
H	-5.294788	4.361474	0.387194
H	-3.623992	4.866625	0.716009
H	3.791702	3.145792	0.584271
H	0.312219	-1.744278	-1.644100
H	0.720472	-2.468707	-3.214838
H	0.810479	-0.712440	-3.001559
H	2.057175	-3.951534	-1.844572
H	2.120816	-3.035785	-0.321703
H	3.226675	-0.923360	-3.915709
H	2.909356	-2.664807	-4.045165
H	4.338612	-2.099513	-3.179386
H	-1.545799	-1.754148	-2.924396
H	-2.008685	-1.205421	-4.551167
H	-3.202696	-1.990771	-3.506691
H	-4.827345	-0.123763	-3.520247
H	-3.825164	0.761753	-4.677548
H	-4.307512	1.540346	-3.155777
H	-1.895759	2.089172	-2.958520
H	-1.346008	1.059247	-4.290610

H	-0.694288	0.828701	-2.650646
H	-4.595957	-2.656540	1.268116
H	-4.571337	-2.250534	2.988601
H	-3.070360	-2.152577	2.035117
H	-6.146053	0.772056	1.293536
H	-6.423851	-0.935339	0.870240
H	-2.816335	0.175313	2.958677
H	-4.096861	1.394989	2.656689
H	-3.138792	-3.060062	-0.999695
H	-6.491551	-0.450555	-1.531619
H	4.453632	0.190648	2.743371
H	5.985205	1.086570	2.779207
H	5.870527	-0.422913	1.862727
H	3.432861	2.337931	2.124616
H	6.694471	0.800266	-0.225223
H	5.806470	2.219179	-0.826490
H	3.620709	-3.453406	-1.193153
H	4.711002	-2.470144	1.015761
H	6.746735	-3.871160	0.912765
H	8.398640	-3.528880	-0.911558
H	7.997320	-1.780127	-2.626014
H	5.957255	-0.378800	-2.513446
H	-4.518116	-4.997300	-1.759054
H	-6.889763	-4.636948	-2.410164
H	-7.870775	-2.362278	-2.301953
H	4.956440	3.213872	1.932548
H	6.770440	2.327306	0.656745
H	2.955425	-1.294273	1.790918
H	1.943878	-1.149114	3.222798
H	-1.883035	-2.830541	3.942335
H	-0.177758	-3.134405	3.562102
H	-0.965346	-4.002256	-0.836964
H	0.237088	-4.009696	0.460221
H	-0.490422	-2.580002	5.993336
H	0.504438	-1.350308	5.178173
H	-1.198695	-1.014434	5.533053
H	3.893925	-2.695243	3.605100

H	2.337144	-3.534739	3.801797
H	3.272867	-3.762036	2.318613
H	-1.543384	-5.677319	0.906224
H	-1.563462	-4.408820	2.147325
H	-2.783052	-4.403981	0.866156
H	2.065251	3.880221	1.950501
H	0.278919	5.555131	1.943136
H	-2.029276	4.916749	2.625833
H	-2.525024	2.588611	3.240818
H	-0.732010	0.859916	3.232765
H	1.577797	1.516002	2.649525

5.12 Intermediate 11a

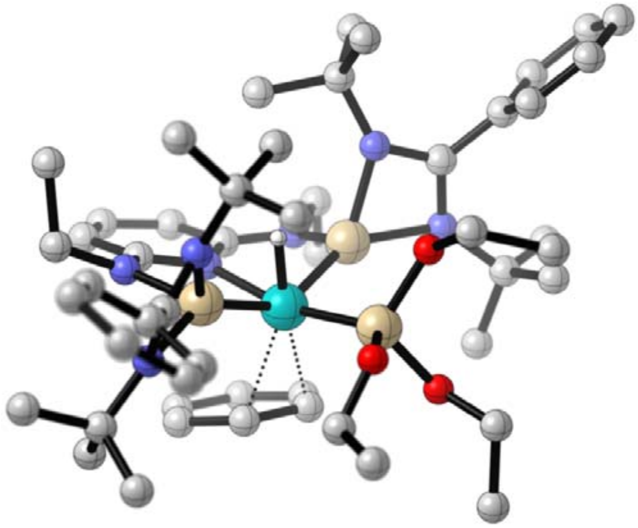

C	0.910938	3.154166	-0.997384
N	-0.183386	2.614335	-0.368122
C	-1.392479	3.271164	-0.457175
C	-1.475481	4.593459	-0.952287
C	-0.321286	5.181761	-1.473845
C	0.873453	4.462030	-1.538641
Fe	-0.012994	0.796268	0.635239
Si	-0.106154	-1.322812	1.329236
O	-0.859526	-1.492437	2.878531
C	-0.826235	-2.645774	3.709711
C	-0.450298	-2.245161	5.141531

N	-2.501781	2.517138	-0.123082
C	-3.848515	3.042898	-0.350446
C	-4.341991	3.951328	0.790445
N	2.041763	2.344794	-1.080363
C	3.124311	2.717266	-1.999170
C	2.718353	2.588068	-3.479432
Si	1.968000	0.767667	-0.195556
N	3.869146	0.535324	0.236474
C	4.945247	1.168023	1.041081
C	4.327635	2.416181	1.688324
N	2.821159	-0.520181	-1.350560
C	2.406976	-1.618365	-2.275691
C	2.562800	-3.013964	-1.624827
C	3.980493	-0.446314	-0.670041
C	5.167180	-1.334826	-0.837302
C	5.379726	-2.388721	0.071700
C	6.497636	-3.223345	-0.066048
C	7.416135	-3.006703	-1.106727
C	7.210199	-1.953137	-2.012345
C	6.088406	-1.121283	-1.879894
C	3.220815	-1.538302	-3.588945
C	0.923289	-1.413774	-2.621320
C	6.138813	1.612039	0.161619
C	5.451590	0.235467	2.166808
Si	-2.099869	0.766951	0.118670
N	-3.776757	-0.072919	0.542582
C	-4.418634	-0.635616	1.762814
C	-5.949981	-0.434527	1.699042
N	-2.903106	0.041052	-1.438556
C	-3.875358	-0.533713	-0.724285
C	-4.784408	-1.598350	-1.230436
C	-4.236504	-2.882294	-1.421975
C	-5.043904	-3.922189	-1.902546
C	-6.393761	-3.681273	-2.211675
C	-6.936340	-2.397961	-2.033991
C	-6.134884	-1.357578	-1.538766
C	-2.782395	0.217922	-2.902908

C	-4.099896	0.777047	-3.488879
C	-1.663465	1.258761	-3.136341
C	-2.428108	-1.117884	-3.595190
C	-3.870453	0.168754	2.952336
C	-4.069862	-2.126668	1.955503
O	-0.978070	-2.341555	0.233998
C	-0.772127	-3.730308	-0.018431
C	-1.712735	-4.626292	0.800760
O	1.289797	-2.338462	1.522020
C	2.313456	-1.972558	2.432266
C	3.007953	-3.236160	2.953134
C	0.804430	2.007590	2.486441
H	-6.386273	-0.698774	2.676060
H	-4.319109	-0.207679	3.884696
H	0.132958	0.176361	-0.721463
H	1.756925	4.897992	-1.998604
H	-0.360868	6.201867	-1.863631
H	-2.423097	5.126021	-0.951703
H	3.963306	2.039903	-1.793927
H	3.485703	3.739374	-1.781829
H	2.418928	1.549141	-3.685820
H	3.561046	2.853196	-4.140573
H	1.867550	3.245934	-3.710648
H	-4.522490	2.177782	-0.438303
H	-3.889442	3.583778	-1.315269
H	-4.354420	3.389694	1.736938
H	-5.361557	4.316895	0.580233
H	-3.675488	4.816456	0.919258
H	3.887427	3.068039	0.920332
H	0.281265	-1.584557	-1.747359
H	0.648397	-2.133574	-3.408180
H	0.745393	-0.400208	-3.006357
H	2.008446	-3.753230	-2.226019
H	2.145862	-2.996009	-0.607863
H	3.138904	-0.534224	-4.034418
H	2.816049	-2.271554	-4.305283
H	4.280054	-1.773724	-3.425550

H	-1.578191	-1.593556	-3.091378
H	-2.160313	-0.927303	-4.647416
H	-3.282123	-1.809121	-3.575349
H	-4.917113	0.046952	-3.402338
H	-3.954293	1.008634	-4.556594
H	-4.384002	1.702899	-2.964797
H	-1.994095	2.260746	-2.828358
H	-1.409587	1.286668	-4.207550
H	-0.766173	1.008934	-2.556168
H	-4.545992	-2.749962	1.186211
H	-4.433258	-2.460497	2.941650
H	-2.982656	-2.258201	1.908668
H	-6.189100	0.619549	1.482615
H	-6.413110	-1.073269	0.935733
H	-2.781380	0.056556	3.013987
H	-4.138286	1.230486	2.840899
H	-3.187025	-3.044096	-1.179089
H	-6.550014	-0.359249	-1.393220
H	4.612039	-0.096039	2.795542
H	6.164007	0.789860	2.799683
H	5.966314	-0.645119	1.760879
H	3.544530	2.121910	2.398397
H	6.673493	0.754605	-0.265684
H	5.796569	2.267819	-0.653265
H	3.613425	-3.331367	-1.585506
H	4.659629	-2.556817	0.871148
H	6.648918	-4.043030	0.639236
H	8.288013	-3.655540	-1.211892
H	7.921686	-1.777239	-2.821660
H	5.925958	-0.297851	-2.576755
H	-4.618108	-4.918560	-2.037710
H	-7.020836	-4.490776	-2.591137
H	-7.983243	-2.206177	-2.277742
H	5.103116	2.973715	2.235408
H	6.847427	2.179035	0.786516
H	3.045734	-1.315936	1.927841
H	1.905177	-1.400518	3.288899

H	-1.826785	-3.121505	3.704527
H	-0.103004	-3.387618	3.325448
H	-0.957744	-3.895564	-1.098362
H	0.272406	-4.015848	0.184532
H	-0.477028	-3.120040	5.814447
H	0.564011	-1.814595	5.162608
H	-1.153763	-1.486181	5.520446
H	3.864105	-2.975952	3.599664
H	2.301224	-3.850839	3.532717
H	3.372364	-3.846604	2.109961
H	-1.548113	-5.686736	0.538866
H	-1.524063	-4.499693	1.876588
H	-2.767356	-4.376683	0.607894
C	1.139890	3.382075	2.244351
C	0.174043	4.370243	2.256961
C	-1.175371	4.046674	2.572344
C	-1.518788	2.735615	2.843651
C	-0.554452	1.679915	2.782126
H	2.170232	3.665034	2.049220
H	0.451079	5.406348	2.050323
H	-1.928725	4.835282	2.622825
H	-2.539382	2.497709	3.136247
H	-0.783680	0.724725	3.245944
H	1.588582	1.303885	2.761529

5.13 Transition State 16a‡

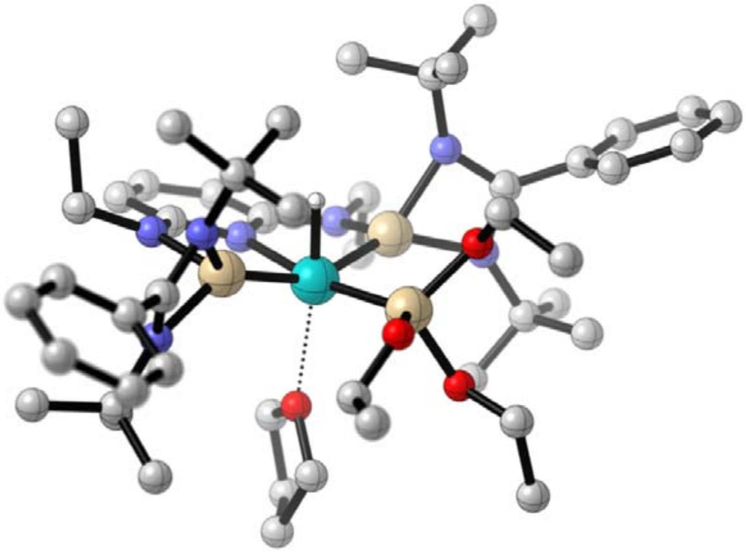

C	0.522786	0.666738	3.378923
O	0.077104	1.417726	2.246735
C	-0.324465	2.734325	2.630663
C	-0.375657	2.717270	4.154279
C	0.704493	1.686341	4.497770
Fe	-0.050819	0.511851	-0.168143
Si	-2.139058	0.787588	-0.535491
N	-3.229863	0.202608	-1.927098
C	-4.102637	-0.304535	-1.062282
C	-5.136942	-1.327689	-1.357003
C	-4.729831	-2.663092	-1.401148
C	-5.664916	-3.654918	-1.672667
C	-6.998611	-3.318459	-1.900086
C	-7.399855	-1.985365	-1.853757
C	-6.468999	-0.986735	-1.581329
Si	1.993829	0.518815	-0.756919
N	2.207375	2.160285	-1.480032
C	1.138457	3.015536	-1.371634
N	-0.011998	2.498515	-0.858888
C	-1.154312	3.242374	-0.914892
C	-1.129477	4.596756	-1.285387
C	0.075598	5.143171	-1.693434
C	1.214848	4.365072	-1.769838

N	-2.325153	2.576435	-0.627990
C	-3.600168	3.259315	-0.762545
C	-3.956744	4.111846	0.455971
N	3.115968	-0.584189	-1.809160
C	2.923637	-1.581943	-2.882703
C	1.554992	-1.301941	-3.511616
C	4.109897	-0.504063	-0.911107
N	3.734439	0.370209	0.010221
C	4.417460	0.955275	1.166953
C	4.497905	-0.054780	2.321288
C	5.403080	-1.242682	-0.921197
C	5.596479	-2.348166	-0.093964
C	6.825610	-2.999082	-0.076516
C	7.869373	-2.544104	-0.878147
C	7.680123	-1.438015	-1.703464
C	6.449663	-0.789578	-1.726993
C	2.908413	-3.011631	-2.322562
C	4.008742	-1.426379	-3.958356
C	3.564698	2.160451	1.594523
C	5.828647	1.448546	0.812869
C	3.410442	2.599723	-2.166772
C	3.250559	2.649544	-3.685959
Si	-0.398897	-1.648176	0.319741
O	0.858792	-2.842127	0.373268
C	1.912922	-2.699995	1.277137
C	2.623827	-4.036429	1.441423
O	-1.107499	-1.905219	1.864764
C	-1.293169	-3.158009	2.469733
C	-1.607446	-2.958679	3.945195
O	-1.441746	-2.535336	-0.726204
C	-1.120752	-3.714755	-1.421582
C	-1.378840	-4.967955	-0.595803
N	-3.777373	0.183015	0.140780
C	-4.339881	-0.095724	1.469392
C	-4.502924	-1.598995	1.732630
C	-5.688590	0.624788	1.605080
C	-3.339514	0.478877	2.480431

C	-3.058672	0.042402	-3.374524
C	-2.385335	-1.297255	-3.689429
C	-4.404184	0.160047	-4.102101
C	-2.145905	1.196318	-3.818562
H	-6.092577	0.487313	2.614535
H	-3.734934	0.366862	3.496183
H	-0.042137	0.138488	-1.658089
H	2.138534	4.788514	-2.138936
H	0.120104	6.189578	-1.984206
H	-2.029797	5.195181	-1.281240
H	4.203225	1.890582	-1.916866
H	3.749818	3.571456	-1.780295
H	2.979097	1.657283	-4.059280
H	4.185573	2.959113	-4.166905
H	2.462291	3.348019	-3.982521
H	-4.367181	2.489606	-0.891283
H	-3.619535	3.867305	-1.678572
H	-4.032517	3.479498	1.346825
H	-4.918089	4.617619	0.311474
H	-3.193865	4.872103	0.651598
H	3.577226	2.938574	0.824999
H	0.757776	-1.472321	-2.785103
H	1.402702	-1.966787	-4.368186
H	1.474719	-0.264609	-3.855564
H	2.637523	-3.714272	-3.118780
H	2.165439	-3.090450	-1.521115
H	4.057612	-0.390593	-4.311716
H	3.774068	-2.069660	-4.813624
H	4.994702	-1.716307	-3.586854
H	-1.461275	-1.390293	-3.114593
H	-2.157932	-1.362379	-4.759702
H	-3.041204	-2.132232	-3.425664
H	-5.057269	-0.692184	-3.897660
H	-4.230565	0.199395	-5.182823
H	-4.922985	1.078792	-3.805209
H	-2.608382	2.165006	-3.599018
H	-1.966725	1.133352	-4.897138

H	-1.181640	1.147940	-3.302271
H	-5.347363	-2.023158	1.184008
H	-4.680217	-1.765289	2.801245
H	-3.586039	-2.117730	1.445032
H	-5.571464	1.698531	1.421822
H	-6.419949	0.226915	0.894122
H	-2.388173	-0.056112	2.409592
H	-3.169985	1.546040	2.295103
H	-3.687838	-2.892817	-1.191003
H	-6.772477	0.056112	-1.555568
H	3.504335	-0.438201	2.572048
H	4.920661	0.425134	3.210860
H	5.137249	-0.902434	2.057849
H	2.522014	1.866275	1.750175
H	6.512293	0.621754	0.603809
H	5.799684	2.108522	-0.060831
H	3.888518	-3.311264	-1.939009
H	4.780081	-2.704062	0.523869
H	6.965038	-3.864221	0.564235
H	8.829870	-3.049902	-0.859619
H	8.490732	-1.079271	-2.330181
H	6.296896	0.076032	-2.365189
H	-5.350972	-4.694027	-1.702636
H	-7.727020	-4.095253	-2.112658
H	-8.438276	-1.722511	-2.030731
H	3.955449	2.585173	2.525492
H	6.237855	2.018208	1.654350
H	2.621202	-1.924907	0.938173
H	1.548231	-2.369267	2.265876
H	-2.118674	-3.701498	1.983645
H	-0.398229	-3.786841	2.357929
H	-1.743718	-3.743717	-2.327904
H	-0.074403	-3.703959	-1.752247
H	-1.797440	-3.917113	4.442034
H	-0.768668	-2.466982	4.451065
H	-2.492962	-2.324162	4.059195
H	3.447025	-3.960486	2.163905

H	1.920196	-4.793611	1.801902
H	3.025480	-4.383967	0.482702
H	-1.210268	-5.870079	-1.196951
H	-0.709034	-4.991261	0.267749
H	-2.413608	-4.982279	-0.232071
H	0.412886	3.452094	2.248415
H	-1.287517	2.944186	2.159622
H	-1.355185	2.368838	4.499987
H	-0.188064	3.704696	4.585288
H	0.572712	1.240511	5.487337
H	1.699005	2.143921	4.452106
H	-0.229379	-0.091199	3.612884
H	1.442002	0.154316	3.083525

5.14 Intermediate 17a

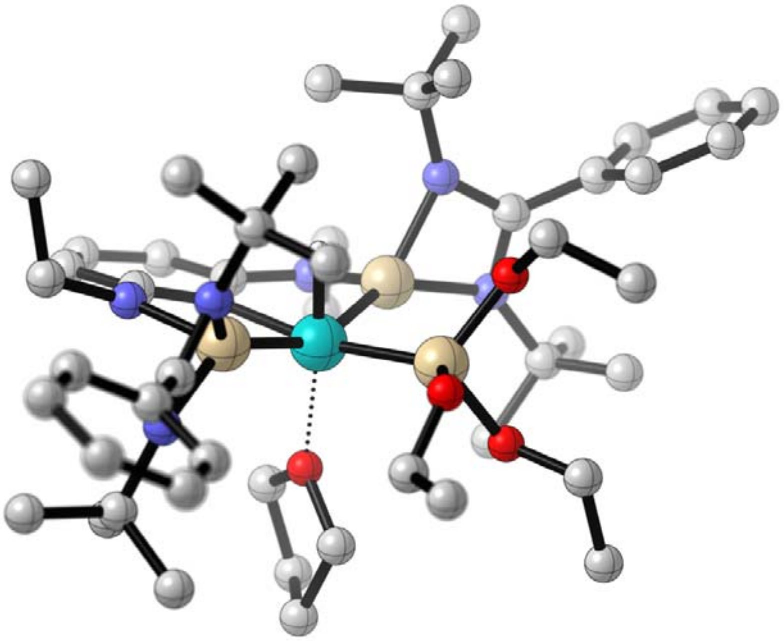

C	5.603242	-2.186979	0.340851
C	5.412895	-1.087083	-0.495755
C	6.458482	-0.646325	-1.308731
C	7.685436	-1.301256	-1.283359
C	7.872314	-2.401023	-0.449171
C	6.829500	-2.843844	0.360302

C	4.118282	-0.351884	-0.493906
N	3.119954	-0.465070	-1.381980
C	2.940949	-1.468338	-2.451074
C	3.013065	-2.897255	-1.893448
N	3.742883	0.547339	0.403191
C	4.434749	1.177307	1.529998
C	3.552319	2.361089	1.956076
Si	1.999511	0.662944	-0.365641
N	2.205827	2.281949	-1.140093
C	3.395726	2.701309	-1.861026
C	3.191726	2.757082	-3.374934
C	1.148982	3.149565	-1.008978
N	0.002977	2.641286	-0.476996
C	-1.133327	3.397482	-0.510805
C	-1.099590	4.754602	-0.869823
C	0.104404	5.291186	-1.294562
C	1.233036	4.500617	-1.398827
Fe	-0.041905	0.668844	0.240095
Si	-0.356291	-1.501847	0.689506
O	-1.099421	-1.793040	2.217847
C	-1.287990	-3.046125	2.818096
C	-1.569410	-2.851755	4.300697
N	-2.306548	2.738772	-0.214567
C	-3.579840	3.426788	-0.344135
C	-3.936949	4.264676	0.883785
Si	-2.129030	0.948396	-0.116525
N	-3.777683	0.349169	0.540806
C	-4.328401	0.039084	1.867699
C	-5.690029	0.731206	2.019643
N	-3.197125	0.354628	-1.518585
C	-3.024083	0.227744	-2.969094
C	-4.368895	0.350805	-3.696500
C	-4.079430	-0.150861	-0.664757
C	-5.097721	-1.186404	-0.967385
C	-4.661224	-2.510203	-1.058248
C	-5.580076	-3.515938	-1.334440
C	-6.925408	-3.203421	-1.524132

C	-7.355577	-1.881777	-1.432996
C	-6.441835	-0.869767	-1.151613
C	-2.118783	1.397705	-3.385275
C	-2.336770	-1.098477	-3.313655
C	-3.334888	0.618121	2.883199
C	-4.461022	-1.470659	2.111729
C	5.816167	1.711244	1.123970
C	4.576808	0.194121	2.701564
C	3.989081	-1.264383	-3.555375
C	1.544668	-1.252412	-3.043285
O	-1.349321	-2.369090	-0.415845
C	-1.043110	-3.574817	-1.070166
C	-1.406274	-4.803714	-0.246515
O	0.918901	-2.677085	0.754080
C	1.934025	-2.546634	1.704152
C	2.643510	-3.882180	1.878181
H	-6.084533	0.574436	3.029910
H	-3.726396	0.490500	3.898661
H	-0.042743	0.242465	-1.234220
H	2.156385	4.915576	-1.778743
H	0.156363	6.340130	-1.574553
H	-1.992504	5.363484	-0.841080
H	4.181929	1.975086	-1.639762
H	3.767472	3.664617	-1.482293
H	2.864988	1.776704	-3.736096
H	4.124616	3.023864	-3.884709
H	2.425810	3.487449	-3.651749
H	-4.348188	2.660396	-0.486426
H	-3.593734	4.047634	-1.251362
H	-4.006529	3.622604	1.768194
H	-4.900951	4.767648	0.747328
H	-3.176400	5.026347	1.084066
H	3.509076	3.119289	1.167817
H	0.772500	-1.460199	-2.299757
H	1.402464	-1.925097	-3.895638
H	1.409074	-0.220743	-3.386508
H	2.743880	-3.612553	-2.679154

H	2.306234	-3.006641	-1.064214
H	3.980207	-0.228333	-3.911050
H	3.762016	-1.920123	-4.403037
H	4.997108	-1.507151	-3.209700
H	-1.429729	-1.208653	-2.715569
H	-2.080345	-1.124847	-4.379170
H	-2.995402	-1.945247	-3.098215
H	-5.017950	-0.508179	-3.507919
H	-4.193911	0.409252	-4.776136
H	-4.893176	1.261588	-3.384782
H	-2.585798	2.358267	-3.141166
H	-1.940530	1.362568	-4.465314
H	-1.153707	1.340815	-2.871054
H	-5.291490	-1.907723	1.551464
H	-4.643585	-1.652649	3.176849
H	-3.530369	-1.967274	1.827879
H	-5.596082	1.809055	1.847288
H	-6.417809	0.326797	1.308666
H	-2.374889	0.099466	2.803716
H	-3.182559	1.689563	2.707265
H	-3.610209	-2.723014	-0.877580
H	-6.767798	0.164593	-1.089746
H	3.601071	-0.210120	2.986645
H	5.009526	0.701619	3.571071
H	5.232263	-0.640741	2.436895
H	2.527627	2.031908	2.154813
H	6.522044	0.904871	0.909230
H	5.734106	2.350617	0.238328
H	4.020125	-3.149817	-1.548345
H	4.786073	-2.534158	0.964130
H	6.967425	-3.703359	1.008894
H	8.829783	-2.912544	-0.431155
H	8.495310	-0.952089	-1.916394
H	6.309042	0.214458	-1.954109
H	-5.244070	-4.546499	-1.399480
H	-7.640676	-3.990827	-1.742379
H	-8.402890	-1.638100	-1.582898

H	3.958568	2.823045	2.862263
H	6.231137	2.311858	1.940841
H	2.654446	-1.768861	1.399774
H	1.528900	-2.225735	2.678940
H	-2.130614	-3.576278	2.346835
H	-0.402584	-3.685276	2.685481
H	-1.610585	-3.587808	-2.013012
H	0.020807	-3.620774	-1.334807
H	-1.753071	-3.810960	4.798385
H	-0.716858	-2.365758	4.788926
H	-2.448587	-2.212801	4.437731
H	3.447619	-3.808546	2.621529
H	1.933188	-4.644999	2.213680
H	3.071532	-4.219909	0.927730
H	-1.253093	-5.720585	-0.829037
H	-0.781818	-4.851866	0.649396
H	-2.457022	-4.762836	0.065896
O	0.089515	1.412537	2.253367
C	-0.323389	2.717808	2.657467
C	-0.411359	2.657332	4.178247
C	0.685615	1.643133	4.516323
C	0.549160	0.645889	3.370776
H	0.423039	3.447506	2.316079
H	-1.274678	2.940028	2.169454
H	-1.389825	2.269825	4.483843
H	-0.263102	3.634214	4.646355
H	0.553830	1.171932	5.494007
H	1.669520	2.126346	4.495548
H	-0.180811	-0.142282	3.573470
H	1.489458	0.173294	3.075087

5.15 Transition State 18oa‡

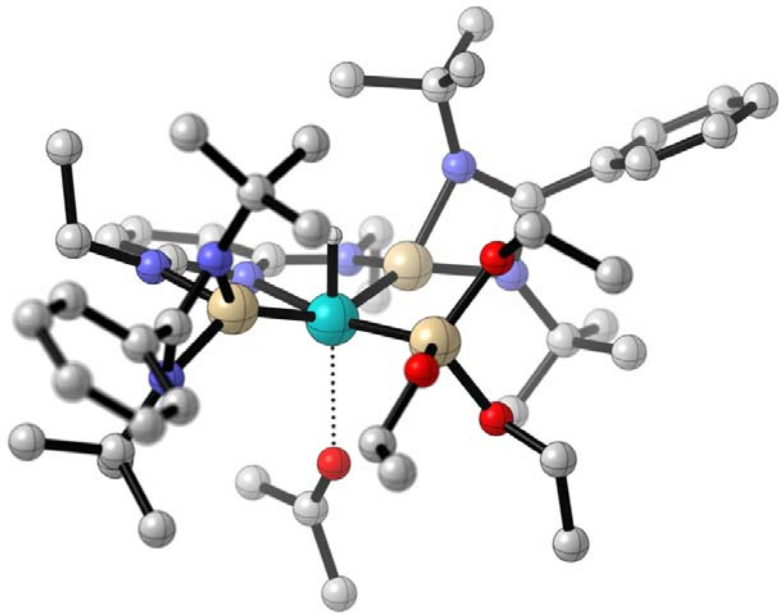

C	5.637070	-2.130804	0.453906
C	5.380239	-1.148907	-0.522687
C	6.367896	-0.841336	-1.478576
C	7.599600	-1.511465	-1.458915
C	7.850032	-2.496700	-0.489160
C	6.865663	-2.806328	0.464384
C	4.096815	-0.398551	-0.507549
N	3.061707	-0.514757	-1.369485
C	2.807280	-1.597410	-2.359294
C	3.756965	-1.441761	-3.569489
Si	1.983658	0.668233	-0.330136
N	3.746789	0.517522	0.401009
C	4.515971	1.249595	1.418893
C	5.891215	1.708907	0.889051
Fe	-0.037479	0.683675	0.342271
O	0.073599	1.310063	2.791788
C	0.015352	2.254485	3.577385
C	-0.020278	3.697899	3.110257
Si	-2.118255	0.957664	-0.077362
N	-2.331193	2.750479	-0.224206
C	-3.622214	3.420112	-0.408089

C	-4.003530	4.310121	0.789407
N	-3.114267	0.287762	-1.535597
C	-2.887905	0.102739	-2.981585
C	-1.835071	1.160895	-3.382274
C	-4.051423	-0.193876	-0.711031
N	-3.809782	0.345072	0.502181
C	-4.397036	0.027459	1.819213
C	-3.468277	0.706042	2.842319
C	-5.055403	-1.241038	-1.031785
C	-4.614613	-2.578463	-1.089420
C	-5.532766	-3.599923	-1.368082
C	-6.885351	-3.292328	-1.597467
C	-7.321079	-1.958180	-1.543032
C	-6.408145	-0.931099	-1.256243
C	-2.338134	-1.312103	-3.275295
C	-4.190727	0.357008	-3.770303
C	-4.430524	-1.492726	2.093862
C	-5.817514	0.627433	1.905214
Si	-0.319672	-1.464973	0.884268
O	-1.232195	-2.371027	-0.271606
C	-0.969417	-3.689157	-0.744181
C	-1.523996	-4.772096	0.190836
N	-0.007541	2.654959	-0.446906
C	-1.157769	3.417738	-0.526761
C	-1.128201	4.771723	-0.927987
C	0.080772	5.306960	-1.381466
C	1.214931	4.499858	-1.474598
C	1.136277	3.154870	-1.033915
N	2.190410	2.266893	-1.162325
C	3.385332	2.659739	-1.914229
C	3.142097	2.694534	-3.434286
O	1.007856	-2.574772	1.064338
C	2.012288	-2.305462	2.028230
C	2.789226	-3.595366	2.316237
O	-1.117559	-1.645226	2.399893
C	-1.221397	-2.848430	3.147500
C	-1.410456	-2.502137	4.628953

C	4.695289	0.381647	2.686658
C	3.654826	2.480182	1.773380
C	1.351676	-1.439787	-2.842502
C	2.968339	-2.996148	-1.722495
C	0.005521	1.989855	5.073956
H	-6.223677	0.472402	2.918326
H	-3.829368	0.508211	3.863637
H	-0.000999	0.167590	-1.065102
H	2.138477	4.893915	-1.891140
H	0.129487	6.352346	-1.695724
H	-2.031373	5.375916	-0.910784
H	4.168330	1.922693	-1.692588
H	3.767865	3.634991	-1.558402
H	2.835028	1.694953	-3.778635
H	4.060604	2.988134	-3.970936
H	2.342525	3.406982	-3.685790
H	-4.381603	2.634495	-0.532271
H	-3.614432	4.014510	-1.340886
H	-4.110780	3.693509	1.695275
H	-4.958844	4.829694	0.602464
H	-3.225417	5.065385	0.979481
H	3.564805	3.159485	0.914511
H	0.644296	-1.617601	-2.023799
H	1.159962	-2.169086	-3.644917
H	1.174724	-0.426873	-3.236752
H	2.649606	-3.762371	-2.448467
H	2.336626	-3.067736	-0.825010
H	3.691626	-0.422654	-3.982617
H	3.465248	-2.158227	-4.354588
H	4.798788	-1.649198	-3.289701
H	-1.523642	-1.540606	-2.577516
H	-1.966974	-1.357363	-4.312781
H	-3.126154	-2.070015	-3.155997
H	-4.955832	-0.396332	-3.535824
H	-3.975497	0.310571	-4.850120
H	-4.589498	1.357191	-3.534610
H	-2.209038	2.175503	-3.173943

H	-1.619728	1.073830	-4.458920
H	-0.905185	1.009424	-2.814243
H	-5.192739	-1.997464	1.484475
H	-4.671043	-1.662097	3.156282
H	-3.442262	-1.918539	1.881006
H	-5.790159	1.709411	1.698350
H	-6.492276	0.141030	1.184775
H	-2.449693	0.305340	2.726614
H	-3.448635	1.795281	2.675116
H	-3.565353	-2.791246	-0.884601
H	-6.738089	0.108470	-1.216359
H	3.715983	0.030696	3.044808
H	5.171180	0.979925	3.481286
H	5.333349	-0.489432	2.478692
H	2.647740	2.131198	2.045431
H	6.553810	0.857121	0.684274
H	5.767126	2.295892	-0.034395
H	4.015280	-3.197330	-1.453653
H	4.865251	-2.373153	1.182815
H	7.052463	-3.575414	1.216713
H	8.808086	-3.020430	-0.476794
H	8.363238	-1.263005	-2.198853
H	6.169837	-0.067339	-2.221264
H	-5.191864	-4.637013	-1.402229
H	-7.598237	-4.090204	-1.816432
H	-8.370654	-1.715976	-1.722655
H	4.102674	3.019288	2.622576
H	6.372011	2.348286	1.647040
H	2.698607	-1.520668	1.658547
H	1.568090	-1.923746	2.969103
H	-2.084968	-3.443743	2.790616
H	-0.318572	-3.472909	3.014137
H	-1.451862	-3.773278	-1.736767
H	0.113711	-3.844746	-0.882723
H	-1.518110	-3.417823	5.236156
H	-0.542993	-1.930807	4.998508
H	-2.311591	-1.881934	4.761048

H	3.598106	-3.410535	3.045418
H	2.115735	-4.364569	2.726958
H	3.232102	-3.991678	1.388062
H	-1.391711	-5.773777	-0.255690
H	-0.998500	-4.743843	1.155679
H	-2.598877	-4.608557	0.376736
H	1.008895	4.024864	2.875670
H	-0.600826	3.779481	2.183657
H	-0.427724	4.369192	3.882486
H	-0.913960	2.405920	5.523932
H	0.053305	0.909653	5.266849
H	0.855230	2.500956	5.563479

5.16 Intermediate 19oa

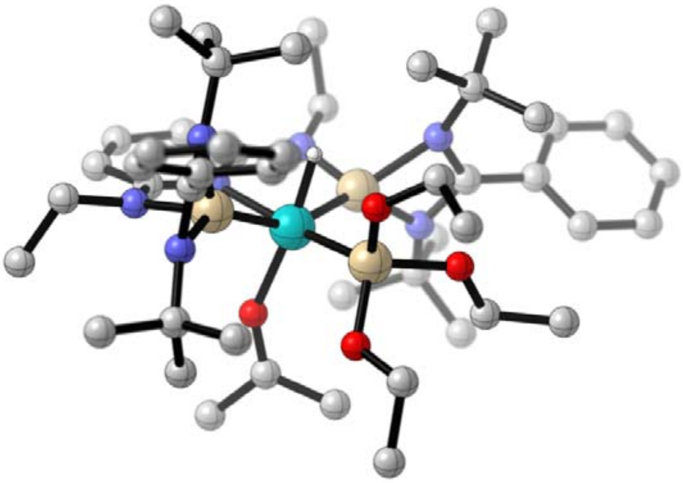

C	6.330101	-0.674130	-1.561784
C	5.344812	-1.052253	-0.629287
C	5.576026	-2.153109	0.217969
C	6.770014	-2.880246	0.118478
C	7.749752	-2.503495	-0.815424
C	7.529449	-1.395977	-1.650964
C	4.074593	-0.286072	-0.522905
N	3.723703	0.511393	0.495285
C	4.523645	1.125518	1.575255
C	5.833779	1.737589	1.029374
N	3.047189	-0.285880	-1.402864

C	2.796032	-1.232330	-2.527240
C	3.697210	-0.868351	-3.729280
Si	1.968711	0.769036	-0.238497
N	2.180900	2.459620	-0.868912
C	3.372533	2.940601	-1.572684
C	3.118501	3.175761	-3.073309
Fe	-0.062174	0.711388	0.395009
Si	-0.301773	-1.472968	0.756866
O	-1.263654	-2.264979	-0.449278
C	-0.996024	-3.482489	-1.136219
C	-1.623329	-4.699507	-0.438822
Si	-2.155314	1.023377	0.034980
N	-2.350762	2.817594	0.129009
C	-3.639190	3.513976	0.079320
C	-3.983470	4.218776	1.404912
N	-3.190718	0.529502	-1.452072
C	-2.969190	0.494098	-2.910378
C	-1.989093	1.651376	-3.210904
C	-4.095718	-0.062988	-0.661192
N	-3.821848	0.334234	0.601611
C	-4.378658	-0.141537	1.889106
C	-3.439740	0.400414	2.989011
C	-5.088358	-1.085120	-1.081573
C	-6.453520	-0.785324	-1.227147
C	-7.349130	-1.790399	-1.625400
C	-6.883259	-3.092518	-1.872054
C	-5.518021	-3.390562	-1.720693
C	-4.618249	-2.390459	-1.329071
C	-2.324431	-0.847858	-3.324274
C	-4.288941	0.730079	-3.673339
C	-4.415471	-1.685114	1.970479
C	-5.794962	0.446533	2.078486
O	-0.086697	1.490864	2.216275
C	0.244739	1.325878	3.406783
C	-0.007983	2.443209	4.405415
N	-0.034806	2.755245	-0.168402
C	1.119511	3.325997	-0.644159

C	1.203733	4.720724	-0.884493
C	0.070222	5.506767	-0.668173
C	-1.137372	4.922453	-0.278054
C	-1.175049	3.520736	-0.082712
C	1.321163	-1.069951	-2.943460
C	3.021437	-2.703352	-2.108748
C	4.850546	0.093508	2.680242
C	3.650648	2.251127	2.175580
O	1.022315	-2.609801	0.737363
C	2.029531	-2.579975	1.730791
C	2.799240	-3.908355	1.721915
O	-1.051901	-1.876736	2.268697
C	-1.223851	-3.207060	2.745175
C	-1.396826	-3.184706	4.268822
C	0.918853	0.059452	3.863034
H	-6.189695	0.148196	3.063706
H	-3.863039	0.157197	3.976791
H	-0.018733	0.348861	-1.071415
H	2.131026	5.171601	-1.229204
H	0.122459	6.586648	-0.828037
H	-2.033352	5.525615	-0.154783
H	4.150730	2.174600	-1.456552
H	3.765031	3.859396	-1.097166
H	2.782519	2.236275	-3.538588
H	4.039793	3.513285	-3.578489
H	2.335246	3.933575	-3.223268
H	-4.407849	2.758770	-0.141512
H	-3.647192	4.241028	-0.754510
H	-4.066776	3.476448	2.213510
H	-4.941434	4.760278	1.323144
H	-3.195566	4.936065	1.680964
H	3.422003	3.012909	1.416731
H	0.651743	-1.380829	-2.133311
H	1.129640	-1.695733	-3.829181
H	1.091942	-0.022809	-3.193325
H	2.683788	-3.360923	-2.926810
H	2.431446	-2.925343	-1.207714

H	3.569686	0.191820	-3.998720
H	3.413104	-1.486579	-4.596387
H	4.756144	-1.057667	-3.507688
H	-1.495385	-1.078338	-2.644798
H	-1.951844	-0.778127	-4.359442
H	-3.059668	-1.664616	-3.272779
H	-4.997012	-0.096564	-3.522815
H	-4.071663	0.810353	-4.751093
H	-4.759237	1.669388	-3.339772
H	-2.429522	2.616474	-2.915490
H	-1.769453	1.675066	-4.290023
H	-1.049531	1.511154	-2.654925
H	-5.208319	-2.107265	1.337704
H	-4.607849	-1.988764	3.013097
H	-3.443307	-2.085408	1.658203
H	-5.765782	1.547176	2.028934
H	-6.480742	0.070271	1.304648
H	-2.448011	-0.062346	2.897299
H	-3.340634	1.493822	2.906771
H	-3.559603	-2.599047	-1.180169
H	-6.806761	0.230555	-1.041082
H	3.928671	-0.384928	3.042972
H	5.338793	0.604006	3.526885
H	5.531343	-0.683370	2.305304
H	2.698826	1.846128	2.546808
H	6.501311	0.963419	0.627057
H	5.611621	2.466687	0.236098
H	4.082435	-2.916418	-1.918679
H	4.808049	-2.445178	0.931058
H	6.931715	-3.743050	0.767982
H	8.680096	-3.069903	-0.891644
H	8.290404	-1.094165	-2.373469
H	6.154638	0.189200	-2.204874
H	-5.152401	-4.403373	-1.904401
H	-7.582431	-3.872956	-2.180163
H	-8.408859	-1.556168	-1.743023
H	4.187378	2.723386	3.013258

H	6.359416	2.258024	1.845974
H	2.722899	-1.726896	1.547366
H	1.593161	-2.426257	2.734179
H	-2.120207	-3.661304	2.275754
H	-0.356272	-3.837333	2.465020
H	-1.423683	-3.380169	-2.153689
H	0.090993	-3.644893	-1.244123
H	-1.587000	-4.203376	4.652105
H	-0.494129	-2.788007	4.757156
H	-2.247053	-2.543912	4.541466
H	3.601836	-3.900424	2.481220
H	2.117603	-4.734867	1.945014
H	3.245914	-4.086423	0.731661
H	-1.479639	-5.609058	-1.049622
H	-1.155880	-4.857458	0.544982
H	-2.706332	-4.545938	-0.287010
H	0.224686	-0.784439	3.733478
H	1.766686	-0.139168	3.194381
H	1.260173	0.121353	4.903586
H	0.935189	2.733881	4.902938
H	-0.441657	3.317155	3.894202
H	-0.696353	2.106684	5.204754

5.17 Transition State 20oa‡

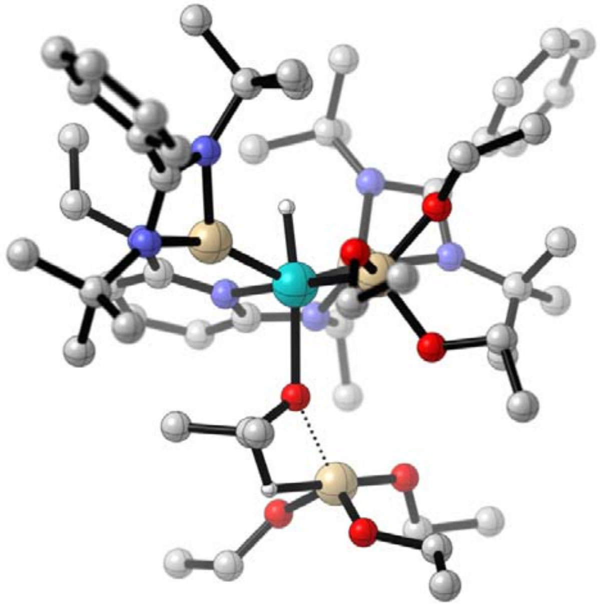

C	-6.163049	0.601169	1.620953
C	-5.796482	0.813869	0.278216
C	-6.756737	1.296092	-0.632774
C	-8.062034	1.575490	-0.204152
C	-8.420747	1.370200	1.138469
C	-7.467588	0.884935	2.049313
C	-4.438951	0.440282	-0.210422
N	-3.414513	1.277172	-0.474501
C	-3.353533	2.725856	-0.112424
C	-3.763518	2.965406	1.361521
N	-4.041488	-0.807709	-0.484213
C	-4.833535	-2.062042	-0.505713
C	-3.984210	-3.108625	-1.249235
Si	-2.185959	-0.187441	-0.772690
N	-2.137491	-0.449412	-2.584272
C	-3.275599	-0.260561	-3.488687
C	-3.146740	1.024511	-4.327335
C	-0.906263	-0.814205	-3.112022
N	0.163595	-0.735888	-2.250904
C	1.441510	-0.895498	-2.749537
C	1.657900	-1.421173	-4.044113
C	0.548748	-1.603062	-4.879529

C	-0.732139	-1.254523	-4.448335
Fe	-0.110979	-0.281385	-0.270132
Si	-0.307292	0.235336	1.891930
O	0.598838	-0.739137	3.005367
C	0.957958	-0.222668	4.285904
C	1.009841	-1.363532	5.305375
N	2.459648	-0.385279	-1.949802
C	3.791260	-0.210083	-2.541102
C	4.652968	-1.480203	-2.441673
Si	1.769852	0.674955	-0.621985
N	3.284351	1.538233	0.184447
C	4.035826	1.371570	1.460839
C	5.547168	1.544122	1.184947
N	2.031581	2.419993	-1.354453
C	1.611319	3.183112	-2.550617
C	2.825579	3.415617	-3.481707
C	2.995511	2.685206	-0.464315
C	3.567986	4.032403	-0.187827
C	2.821163	4.940618	0.588305
C	3.338007	6.211830	0.870494
C	4.596550	6.588703	0.370091
C	5.340136	5.686839	-0.407866
C	4.830248	4.407765	-0.681126
C	0.580908	2.306771	-3.294678
C	0.964913	4.536532	-2.172407
C	3.773954	-0.065622	1.931271
C	3.562124	2.344274	2.560154
C	-6.178016	-1.910080	-1.259210
C	-5.092469	-2.598738	0.921086
C	-4.257042	3.544141	-1.066027
C	-1.906757	3.225090	-0.290034
O	0.196909	1.858416	2.276203
C	-0.477190	2.776398	3.129839
C	0.517636	3.799839	3.691003
O	-1.879775	0.264768	2.649445
C	-2.398254	-0.746710	3.494117
C	-2.797215	-0.149920	4.852111

H	6.115924	1.272848	2.089484
H	4.342993	-0.254477	2.856170
H	-0.670128	1.057664	-0.645894
H	-1.581762	-1.339634	-5.121979
H	0.692587	-1.983401	-5.893962
H	2.665659	-1.618466	-4.401552
H	-4.178198	-0.203944	-2.865187
H	-3.405815	-1.140757	-4.145932
H	-3.070324	1.892018	-3.654015
H	-4.025123	1.157679	-4.982269
H	-2.241371	0.991167	-4.951987
H	4.288812	0.597356	-1.982683
H	3.702530	0.125744	-3.592950
H	4.784429	-1.733942	-1.382607
H	5.642776	-1.320671	-2.903319
H	4.161793	-2.333961	-2.932999
H	-3.682138	-2.742765	-2.240967
H	-1.214161	2.734619	0.405155
H	-1.893383	4.311409	-0.102799
H	-1.558654	3.045697	-1.316907
H	-3.493825	3.995931	1.645843
H	-3.230884	2.260035	2.014905
H	-3.973768	3.359120	-2.114437
H	-4.129085	4.618450	-0.853650
H	-5.317988	3.296959	-0.932301
H	0.186643	4.390745	-1.410505
H	0.501274	4.977692	-3.069962
H	1.711206	5.244702	-1.787988
H	3.559347	4.087993	-3.013579
H	2.485654	3.876544	-4.423752
H	3.312173	2.455874	-3.715553
H	1.060550	1.410599	-3.711892
H	0.145719	2.886350	-4.123671
H	-0.219953	1.979172	-2.618874
H	3.855428	3.382211	2.354326
H	4.018660	2.045805	3.518621
H	2.469956	2.279824	2.651140

H	5.862932	0.882548	0.361556
H	5.791248	2.583816	0.923730
H	2.705804	-0.229082	2.130837
H	4.099384	-0.782365	1.170872
H	1.847719	4.633604	0.972303
H	5.404857	3.698239	-1.278472
H	-4.139080	-2.714403	1.455492
H	-5.579852	-3.586489	0.859016
H	-5.748384	-1.927229	1.490061
H	-3.087058	-3.342734	-0.669579
H	-6.902686	-1.304478	-0.702821
H	-6.016594	-1.455580	-2.249666
H	-4.845306	2.851375	1.509260
H	-5.413559	0.237540	2.324913
H	-7.738356	0.727703	3.095464
H	-9.437471	1.586393	1.472982
H	-8.799375	1.945682	-0.919464
H	-6.478563	1.429213	-1.679050
H	2.759222	6.907815	1.481194
H	4.996468	7.581309	0.587534
H	6.317433	5.975726	-0.799968
H	-4.569389	-4.034010	-1.368384
H	-6.613265	-2.911865	-1.405544
H	-3.288862	-1.196161	3.015501
H	-1.661131	-1.553441	3.651455
H	1.950697	0.264066	4.220013
H	0.245051	0.547450	4.631015
H	-1.269856	3.299384	2.564979
H	-0.976500	2.252705	3.964676
H	1.359199	-0.995467	6.285928
H	0.010596	-1.809009	5.431876
H	1.697576	-2.151387	4.960307
H	-3.307120	-0.900538	5.482106
H	-1.901376	0.207728	5.386052
H	-3.473539	0.709061	4.706078
H	0.001873	4.511956	4.358695
H	1.307613	3.287031	4.262723

H	1.000678	4.368345	2.879917
O	0.639809	-2.298313	0.032803
C	-0.155108	-3.291225	-0.000863
C	-0.717999	-3.625196	-1.395473
C	-1.150127	-3.397624	1.157801
H	-0.607271	-3.321889	2.109016
H	-1.836358	-2.541744	1.080612
H	-1.716038	-4.341705	1.114836
H	-1.266271	-4.580602	-1.386650
H	-1.393749	-2.826605	-1.712641
H	0.114077	-3.669497	-2.110904
Si	2.197824	-3.546001	0.358074
O	3.708409	-2.902122	0.623433
O	1.867419	-4.359038	1.778895
O	2.445205	-4.386962	-1.058744
C	4.875647	-3.712499	0.374540
C	1.946744	-5.693636	-1.395363
C	2.676940	-4.192719	2.968485
C	6.077085	-3.023442	1.015765
H	4.739472	-4.719715	0.811266
H	5.012445	-3.831244	-0.711034
C	2.151247	-5.907154	-2.892962
H	2.494261	-6.448260	-0.803690
H	0.876646	-5.770904	-1.140954
C	1.966842	-4.903880	4.116717
H	3.679682	-4.620276	2.795873
H	2.794294	-3.120880	3.188566
H	6.983248	-3.633067	0.865150
H	6.238309	-2.032265	0.564716
H	5.910244	-2.889534	2.095409
H	2.550791	-4.793987	5.045470
H	0.970963	-4.464391	4.273660
H	1.850000	-5.976470	3.895267
H	1.787900	-6.905648	-3.186476
H	1.597465	-5.143221	-3.460212
H	3.219009	-5.828239	-3.150561
H	0.687009	-4.161136	0.164993

5.18 Transition State 21a‡

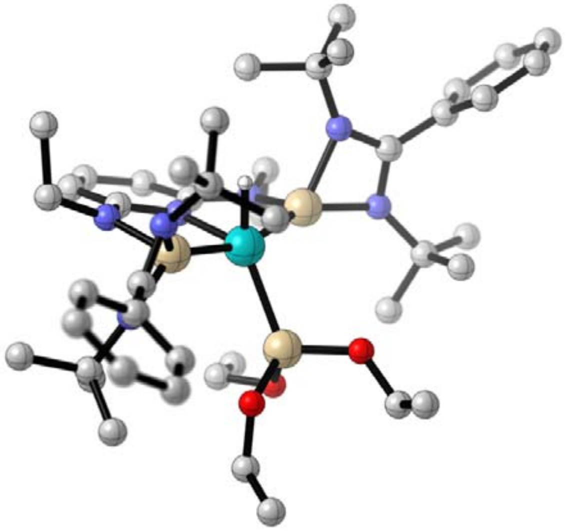

C	-0.777444	-2.291954	2.169251
N	0.257393	-1.439901	1.793272
C	1.454936	-1.541156	2.502303
C	1.568979	-2.354404	3.653414
C	0.496035	-3.157893	4.037092
C	-0.677468	-3.151693	3.283218
Fe	0.026602	-0.107321	0.312586
Si	-0.452028	2.218692	0.515451
O	0.363080	3.134313	-0.665342
C	0.192279	4.521889	-0.942466
C	-0.469730	4.691409	-2.315135
N	2.531956	-0.852385	1.977159
C	3.880468	-1.120061	2.496112
C	4.244077	-0.274116	3.728309
N	-1.927311	-2.226166	1.397616
C	-2.949601	-3.273238	1.492771
C	-2.469162	-4.621000	0.924471
Si	-1.971354	-0.867984	0.236281
N	-3.739402	-0.232138	0.411220
C	-4.587092	0.276962	1.514028
C	-5.652850	-0.767390	1.910161
N	-3.057712	-1.499502	-1.221428
C	-2.685631	-1.729453	-2.649905

C	-3.857866	-2.288405	-3.488376
C	-4.080479	-0.713810	-0.788694
C	-5.314417	-0.342384	-1.529170
C	-5.434471	0.960608	-2.045338
C	-6.591104	1.334547	-2.744244
C	-7.641249	0.416076	-2.909503
C	-7.530228	-0.880589	-2.379062
C	-6.366193	-1.262941	-1.697103
C	-1.559990	-2.784736	-2.649273
C	-2.158096	-0.409158	-3.252967
C	-5.276031	1.605607	1.124798
C	-3.626799	0.527954	2.693087
Si	2.196579	-0.072350	0.377333
N	3.853045	0.834687	0.044762
C	4.188196	2.284544	-0.042193
C	3.516151	2.953388	1.174442
N	3.343058	-1.125248	-0.733280
C	3.348250	-2.523281	-1.211344
C	2.467896	-3.332319	-0.228737
C	4.244949	-0.144797	-0.809584
C	5.445414	-0.144106	-1.686594
C	5.302426	0.000838	-3.079305
C	6.431968	-0.050026	-3.909604
C	7.705111	-0.260317	-3.354946
C	7.848346	-0.410133	-1.964841
C	6.723444	-0.346932	-1.132225
O	-2.099077	2.777942	0.481915
C	-2.574611	3.902901	1.229264
C	-3.386376	4.879960	0.373483
O	0.113432	2.866730	2.005985
C	-0.053082	2.111373	3.206611
C	1.243265	2.161463	4.019242
C	3.625463	2.870422	-1.353750
C	5.713066	2.532556	0.040137
C	2.719262	-2.565315	-2.622430
C	4.762275	-3.143938	-1.230048
H	5.880660	3.604313	0.234205

H	3.607455	4.047316	1.081176
H	0.158888	-1.269892	-0.619846
H	-1.515321	-3.789159	3.553670
H	0.579061	-3.798004	4.918135
H	2.496793	-2.375090	4.217798
H	-3.811468	-2.924554	0.908254
H	-3.296060	-3.392017	2.536218
H	-2.253732	-4.509175	-0.148820
H	-3.242070	-5.397175	1.055139
H	-1.549641	-4.948558	1.431965
H	4.593423	-0.886936	1.693831
H	3.989911	-2.197936	2.716585
H	4.268837	0.791136	3.453996
H	5.238797	-0.561302	4.110302
H	3.506336	-0.399856	4.534504
H	-3.070350	-0.388482	2.943050
H	-0.688109	-2.416426	-2.095409
H	-1.258615	-2.997389	-3.686886
H	-1.904792	-3.721770	-2.184439
H	-1.857859	-0.558463	-4.303706
H	-1.285338	-0.059577	-2.677283
H	-4.350176	-3.115738	-2.952577
H	-3.450006	-2.682197	-4.433023
H	-4.603506	-1.524497	-3.736588
H	1.709568	-2.128358	-2.592460
H	2.646881	-3.608852	-2.970977
H	3.335481	-1.997815	-3.336819
H	5.409572	-2.690018	-1.991888
H	4.667393	-4.218869	-1.452657
H	5.241805	-3.033850	-0.244162
H	2.922770	-3.350844	0.772917
H	2.376731	-4.367008	-0.595834
H	1.465450	-2.888384	-0.150281
H	4.062083	2.346289	-2.219281
H	3.892985	3.937709	-1.431010
H	2.531713	2.768330	-1.368265
H	6.156124	1.959595	0.870030

H	6.231538	2.270704	-0.891413
H	2.446381	2.713111	1.235178
H	4.010684	2.642147	2.109952
H	4.308566	0.151860	-3.503210
H	6.826410	-0.450376	-0.051176
H	-4.549093	2.256196	0.619548
H	-5.632374	2.108877	2.038675
H	-6.141793	1.436353	0.467663
H	-2.907233	1.306663	2.409371
H	-6.345733	-0.948346	1.073947
H	-5.182846	-1.715765	2.205308
H	-2.939996	0.365315	-3.219785
H	-4.624034	1.669564	-1.876021
H	-6.675092	2.344561	-3.150321
H	-8.544688	0.710034	-3.447815
H	-8.346709	-1.595181	-2.501310
H	-6.259700	-2.274125	-1.301127
H	6.316736	0.070479	-4.988687
H	8.582733	-0.306536	-4.002911
H	8.836495	-0.573209	-1.529912
H	-4.199083	0.850259	3.576275
H	-6.234062	-0.386216	2.765446
H	1.192144	4.994178	-0.940143
H	-0.407854	5.026768	-0.166097
H	-3.220296	3.526212	2.049440
H	-1.740463	4.440730	1.713684
H	-0.895699	2.525627	3.794487
H	-0.293245	1.056082	2.970863
H	1.105423	1.690787	5.008138
H	2.036067	1.626773	3.478366
H	1.556527	3.208601	4.164504
H	-0.583065	5.758910	-2.575269
H	0.143986	4.202181	-3.088757
H	-1.460594	4.213538	-2.307040
H	-3.894760	5.611053	1.026077
H	-2.742784	5.431048	-0.325746
H	-4.150958	4.348911	-0.211728

5.19 Intermediate *trans*-14a

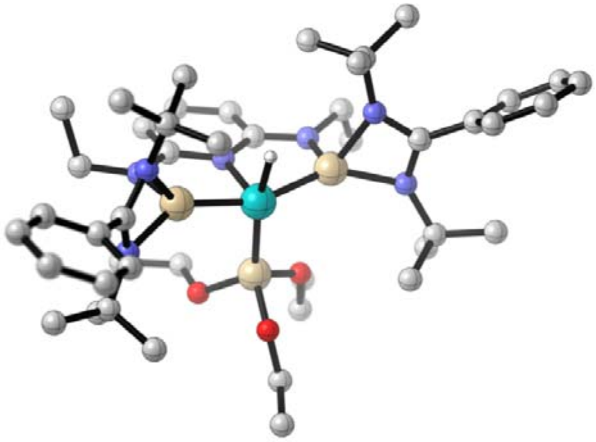

C	-6.406478	-2.243592	-0.160531
C	-5.217966	-1.984222	-0.867832
C	-5.080758	-2.430875	-2.195505
C	-6.125530	-3.138502	-2.808362
C	-7.312986	-3.393134	-2.102419
C	-7.451530	-2.943575	-0.778111
C	-4.116847	-1.213417	-0.232281
N	-3.189062	-1.684884	0.620296
C	-2.809752	-3.089970	0.910493
C	-1.790709	-3.036083	2.070470
N	-3.825948	0.079402	-0.462216
C	-4.507725	1.113986	-1.276883
C	-3.903200	1.094525	-2.699716
Si	-2.130761	-0.092400	0.373184
Fe	-0.027128	0.121680	-0.067272
Si	2.100816	-0.058542	0.175564
N	3.759973	-0.056756	-0.800786
C	4.197362	0.667981	-2.028704
C	3.002542	0.719426	-3.011752
N	0.049990	0.891577	1.795332
C	-1.108379	1.151617	2.521590
C	-1.069754	1.801943	3.775039
C	0.157361	2.197774	4.303219
C	1.335243	1.912352	3.613955
C	1.269297	1.230537	2.377707

N	-2.298121	0.745570	1.943051
C	-3.569025	0.884598	2.665217
C	-3.760725	-0.163899	3.776180
N	2.403927	0.835936	1.697252
C	3.726265	0.991216	2.310621
C	4.306814	2.407885	2.154951
Si	-0.014859	2.231024	-0.895784
O	1.520068	3.028584	-0.821959
C	1.733240	4.410484	-1.111646
C	2.531849	5.069122	0.019615
O	-1.059912	3.477933	-0.326721
C	-0.839461	4.126994	0.925638
C	-2.179162	4.407228	1.611526
O	-0.446212	2.139232	-2.550310
C	-0.269513	3.225006	-3.461791
C	-0.937964	2.879607	-4.795370
N	3.098408	-1.637994	0.524027
C	4.033506	-1.312287	-0.372086
C	5.156796	-2.194980	-0.784360
C	6.460600	-1.929354	-0.326811
C	7.511023	-2.795290	-0.660240
C	7.266075	-3.923122	-1.461569
C	5.966052	-4.185223	-1.925590
C	4.911007	-3.327198	-1.582853
C	2.932645	-2.782344	1.442767
C	2.140102	-3.892625	0.713274
C	4.276639	-3.328922	1.971428
C	2.096911	-2.270719	2.641037
C	5.403281	0.035745	-2.751290
C	4.572788	2.099705	-1.592196
C	-2.137047	-3.707023	-0.338138
C	-4.016438	-3.947629	1.349546
C	-4.205105	2.468782	-0.604960
C	-6.038800	0.920457	-1.337644
H	5.637738	0.672763	-3.618514
H	4.816399	2.706135	-2.479834
H	0.066906	-1.426928	0.201156

H	-1.990867	1.998216	4.316302
H	0.197614	2.720383	5.261362
H	2.297794	2.201835	4.025241
H	-4.370609	0.780095	1.920047
H	-3.656170	1.906296	3.074192
H	-3.812285	-1.166934	3.329318
H	-4.696072	0.026936	4.329847
H	-2.920369	-0.138886	4.486280
H	4.396351	0.269244	1.819960
H	3.677159	0.704939	3.377421
H	4.416177	2.646998	1.089264
H	5.295113	2.473894	2.641817
H	3.641556	3.160566	2.602036
H	-4.656935	2.508937	0.398701
H	-0.944259	-2.389576	1.801212
H	-1.414176	-4.051959	2.270307
H	-2.261496	-2.649203	2.986490
H	-1.788245	-4.729165	-0.114560
H	-1.274936	-3.087923	-0.630550
H	-4.570143	-3.449309	2.161476
H	-3.641218	-4.912528	1.727191
H	-4.706729	-4.150123	0.520507
H	1.192723	-3.476856	0.339447
H	1.924694	-4.720172	1.409445
H	2.722325	-4.289346	-0.132453
H	4.863163	-3.826549	1.188198
H	4.066556	-4.063305	2.765349
H	4.879749	-2.513906	2.402660
H	2.654933	-1.514816	3.212853
H	1.863687	-3.118472	3.304665
H	1.156549	-1.822025	2.291095
H	2.694160	-0.301605	-3.291204
H	3.304013	1.256388	-3.926629
H	2.147567	1.241699	-2.565007
H	6.292518	-0.008842	-2.106928
H	5.185547	-0.977611	-3.117567
H	3.725163	2.559072	-1.070157

H	5.452297	2.077101	-0.928227
H	3.897799	-3.524634	-1.936286
H	6.640438	-1.050124	0.294592
H	-2.819017	1.274890	-2.655736
H	-4.368120	1.885720	-3.311689
H	-4.095520	0.122796	-3.182701
H	-3.124828	2.644235	-0.517100
H	-6.332490	0.053923	-1.944125
H	-6.459182	0.803305	-0.325881
H	-2.853463	-3.758530	-1.173323
H	-4.159902	-2.218409	-2.740597
H	-6.013614	-3.486021	-3.837408
H	-8.127875	-3.938923	-2.582299
H	-8.373776	-3.138181	-0.226914
H	-6.504138	-1.893468	0.868221
H	5.773274	-5.058425	-2.552174
H	8.085456	-4.595597	-1.723636
H	8.519859	-2.589897	-0.296637
H	-4.637476	3.278563	-1.214330
H	-6.477260	1.822787	-1.792168
H	2.295364	4.493757	-2.062715
H	0.772684	4.938023	-1.248132
H	-0.206551	3.518345	1.594499
H	-0.304294	5.080128	0.750299
H	-0.710774	4.149624	-3.044317
H	0.810054	3.412983	-3.619267
H	-2.016785	4.940859	2.563449
H	-2.822603	5.022339	0.961653
H	-2.696224	3.460591	1.819322
H	2.706626	6.137442	-0.197746
H	1.980216	4.984291	0.968449
H	3.506406	4.572740	0.144846
H	-0.795259	3.696136	-5.524205
H	-0.503336	1.955527	-5.210343
H	-2.018057	2.719063	-4.650835

5.20 Transition State 22oa‡

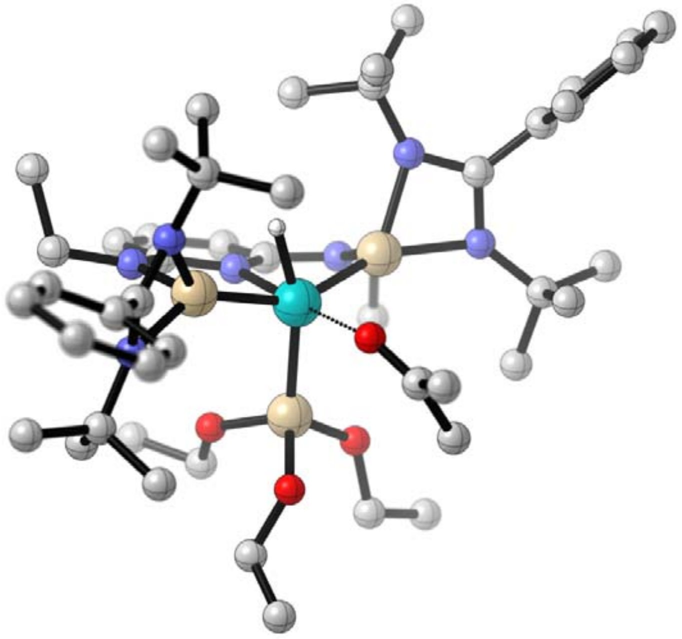

C	4.834674	-3.321014	-1.157686
C	5.148910	-2.204585	-0.359981
C	6.453064	-2.056846	0.149553
C	7.433185	-3.016773	-0.136676
C	7.118663	-4.128580	-0.936894
C	5.819092	-4.277815	-1.448033
C	4.093035	-1.210998	-0.019066
N	3.159604	-1.351749	0.915679
C	2.891163	-2.425392	1.888989
C	1.970471	-1.809619	2.967081
Si	2.167291	0.167791	0.237276
N	2.482468	1.370694	1.540684
C	3.770434	1.996049	1.848229
C	3.765410	3.504290	1.535528
Fe	0.012427	0.247554	0.005280
O	-0.338995	-0.829760	-2.279528
C	-0.150755	-0.588895	-3.483083
C	-0.658271	-1.574310	-4.523929
Si	-1.937357	-0.294686	0.705524
N	-1.974160	0.259727	2.400645
C	-3.132707	0.121117	3.290199

C	-2.917385	-0.912648	4.411147
N	-2.820199	-1.993451	0.678273
C	-2.302974	-3.382749	0.617660
C	-3.412239	-4.455862	0.684479
C	-3.913562	-1.451143	0.101356
N	-3.776740	-0.122128	0.169588
C	-4.668247	0.974309	-0.262111
C	-6.157992	0.672401	0.012482
C	-5.017561	-2.181513	-0.576887
C	-6.105921	-2.687838	0.156938
C	-7.138827	-3.368978	-0.500232
C	-7.088243	-3.551097	-1.892984
C	-6.001292	-3.048098	-2.626739
C	-4.967522	-2.361489	-1.971809
C	-1.393260	-3.569960	1.852081
C	-1.486391	-3.562116	-0.683764
C	-4.446467	1.225722	-1.770906
C	-4.259865	2.215909	0.561187
N	0.212640	0.977350	1.938137
C	-0.848284	0.943829	2.822076
C	-0.783044	1.573743	4.087829
C	0.385051	2.238060	4.455349
C	1.488624	2.235863	3.603057
C	1.396167	1.558116	2.365605
Si	-0.301517	2.398786	-0.653527
O	-1.360541	2.650966	-2.012954
C	-2.190744	3.799980	-2.157799
C	-2.673072	3.887004	-3.609135
N	3.865522	-0.023728	-0.639877
C	4.395839	0.430695	-1.946578
C	3.916024	1.883660	-2.122906
C	3.838222	-0.470322	-3.075502
C	5.941369	0.428334	-1.988630
O	1.097431	3.333767	-1.097860
C	1.034233	4.518028	-1.884250
C	2.432262	5.132503	-2.018239
O	-1.040329	3.340434	0.578538

C	-0.758718	4.681897	0.937656
C	-1.267636	4.937641	2.359960
C	2.148868	-3.575245	1.168025
C	4.177999	-2.948578	2.561797
C	0.522874	0.663511	-3.982966
H	6.268920	0.962254	-2.895513
H	4.172742	2.235157	-3.135068
H	-0.214906	-1.189832	0.638540
H	-1.637301	1.550200	4.758115
H	0.441713	2.747776	5.420116
H	2.419130	2.712740	3.897543
H	-3.983555	-0.188239	2.667070
H	-3.398726	1.106287	3.713901
H	-2.812014	-1.916836	3.975907
H	-3.776399	-0.914942	5.103910
H	-2.005520	-0.686215	4.983377
H	4.524101	1.495710	1.223775
H	4.050478	1.814011	2.903582
H	3.474728	3.657205	0.486808
H	4.762245	3.942891	1.714493
H	3.031143	4.031080	2.163610
H	-4.472197	2.048104	1.628749
H	-0.630828	-2.783389	1.903871
H	-0.889154	-4.547123	1.789747
H	-1.994826	-3.544149	2.773891
H	-1.044574	-4.572649	-0.714590
H	-0.689562	-2.808038	-0.742943
H	-4.082749	-4.274089	1.538863
H	-2.929792	-5.436676	0.824351
H	-4.011675	-4.499459	-0.233689
H	1.281635	-3.166398	0.630499
H	1.799895	-4.320814	1.901157
H	2.819832	-4.075001	0.452807
H	4.822404	-3.491544	1.857587
H	3.894299	-3.638477	3.372902
H	4.749509	-2.113653	2.998147
H	2.481737	-0.987746	3.490974

H	1.697148	-2.585346	3.699932
H	1.056394	-1.411980	2.505367
H	4.262070	-1.483131	-3.002709
H	4.098578	-0.052528	-4.062157
H	2.745406	-0.538392	-2.991119
H	6.347652	0.955347	-1.110734
H	6.356885	-0.586852	-2.021372
H	2.835093	1.994647	-1.969303
H	4.416459	2.535609	-1.390812
H	3.821509	-3.433293	-1.545959
H	6.689057	-1.191051	0.770078
H	-3.378223	1.387125	-1.968864
H	-5.010574	2.116470	-2.092985
H	-4.802819	0.359546	-2.351296
H	-3.193388	2.452632	0.453174
H	-6.550096	-0.118998	-0.639009
H	-6.308704	0.377164	1.063818
H	-2.149120	-3.443593	-1.556440
H	-4.117516	-1.968830	-2.532208
H	-5.957982	-3.188919	-3.708775
H	-7.893485	-4.082754	-2.404048
H	-7.983208	-3.757587	0.072798
H	-6.137280	-2.540799	1.237597
H	5.569877	-5.139489	-2.070776
H	7.883209	-4.875576	-1.159917
H	8.441753	-2.897807	0.264425
H	-4.849026	3.082600	0.221299
H	-6.737825	1.590300	-0.175580
H	0.968224	0.520508	-4.979964
H	-0.233841	1.467255	-4.028407
H	1.277092	0.996864	-3.258912
H	0.171001	-1.924400	-5.163683
H	-1.139012	-2.432953	-4.034922
H	-1.383649	-1.075646	-5.193569
H	0.619520	4.290154	-2.885653
H	0.363921	5.265818	-1.418697
H	0.328522	4.878121	0.885411

H	-1.251538	5.381723	0.229490
H	-3.057839	3.728531	-1.477435
H	-1.649701	4.725211	-1.892482
H	-1.055859	5.976756	2.668132
H	-2.356134	4.769191	2.410747
H	-0.777687	4.242909	3.058049
H	2.387980	6.063855	-2.609040
H	2.838332	5.368070	-1.021387
H	3.122201	4.433896	-2.515047
H	-3.348054	4.749472	-3.747267
H	-1.814000	3.999859	-4.291244
H	-3.215392	2.967842	-3.884490

5.21 Intermediate 23oa

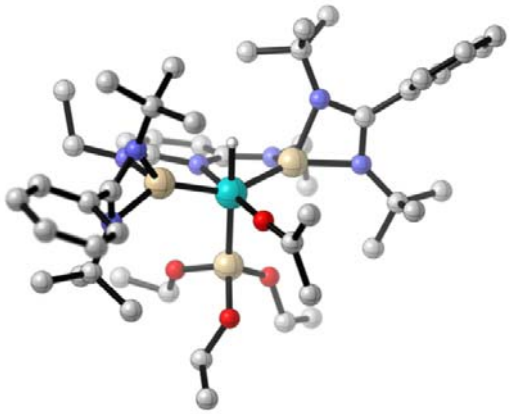

C	6.461257	-1.964535	-0.089001
C	5.134794	-2.203880	-0.494359
C	4.829998	-3.367254	-1.225429
C	5.843806	-4.279434	-1.552356
C	7.165282	-4.038902	-1.143964
C	7.472017	-2.881582	-0.410788
C	4.047460	-1.251622	-0.128133
N	3.756364	-0.077201	-0.744156
C	4.252101	0.446965	-2.037854
C	3.581714	1.819721	-2.219366
N	3.147579	-1.418310	0.839409
C	3.048444	-2.387208	1.950976
C	1.979899	-1.837687	2.924159

Si	2.134177	0.096734	0.232196
N	2.468352	1.249382	1.564388
C	3.795731	1.683126	1.999234
C	4.051518	3.172520	1.706836
Fe	0.000449	0.226310	-0.005842
O	-0.160337	-0.451318	-1.825975
C	-0.025184	-0.694404	-3.038739
C	0.110164	0.420179	-4.059756
Si	-1.974248	-0.333891	0.653120
N	-2.938008	-1.990124	0.650811
C	-2.587526	-3.428107	0.706858
C	-3.753832	-4.283408	1.255157
N	-3.782008	-0.083347	0.077176
C	-4.565859	1.069229	-0.418811
C	-6.090878	0.828510	-0.384280
C	-3.994100	-1.403892	0.043583
C	-5.128648	-2.082116	-0.645346
C	-5.084620	-2.237664	-2.043807
C	-6.171221	-2.806248	-2.724058
C	-7.308774	-3.218497	-2.012380
C	-7.353368	-3.067869	-0.616805
C	-6.267124	-2.504549	0.065743
C	-4.097614	1.373817	-1.860770
C	-4.229588	2.255443	0.511097
N	-2.025144	0.226355	2.346687
C	-0.904864	0.921911	2.767806
N	0.169953	0.932337	1.895753
C	1.357459	1.490577	2.351856
C	1.429710	2.201941	3.568685
C	0.301456	2.259009	4.387391
C	-0.862428	1.593333	4.011988
C	-3.187803	0.082961	3.227270
C	-2.966614	-0.926700	4.368019
Si	-0.164289	2.410064	-0.689345
O	-0.982703	2.716947	-2.196970
O	1.271965	3.377592	-0.795015
O	-1.092742	3.295443	0.455471

C	2.593849	-3.764066	1.415746
C	4.392244	-2.517012	2.702522
C	3.853395	-0.483911	-3.206860
C	5.784887	0.643144	-1.997798
C	-1.391567	-3.574605	1.670320
C	-2.173178	-3.922477	-0.695774
C	-0.006005	-2.157098	-3.480639
H	6.104638	1.167349	-2.911427
H	3.890819	2.259440	-3.178231
H	0.071012	-1.274908	0.428742
H	-1.732055	1.594633	4.661225
H	0.338680	2.803595	5.331918
H	2.356721	2.680770	3.866910
H	-4.029238	-0.249137	2.603456
H	-3.473059	1.068797	3.633962
H	-2.753520	-1.918915	3.945856
H	-3.866539	-0.997801	5.000031
H	-2.116517	-0.629287	4.997639
H	4.530312	1.071409	1.455212
H	3.931035	1.472970	3.075921
H	3.941749	3.359365	0.631170
H	5.062206	3.468346	2.032259
H	3.312389	3.800168	2.223550
H	-4.584896	2.048625	1.532223
H	-0.531555	-3.004865	1.300744
H	-1.118701	-4.637537	1.749511
H	-1.648914	-3.200639	2.671663
H	-1.847824	-4.973778	-0.648903
H	-1.339607	-3.304865	-1.055999
H	-4.132970	-3.850536	2.193064
H	-3.386608	-5.300182	1.463243
H	-4.578040	-4.359957	0.537067
H	1.645625	-3.656751	0.870649
H	2.440245	-4.454643	2.259538
H	3.350925	-4.193898	0.746067
H	5.159294	-2.997634	2.081997
H	4.243789	-3.128281	3.605795

H	4.749316	-1.522332	3.008986
H	2.320243	-0.899364	3.383458
H	1.800694	-2.577780	3.717865
H	1.039656	-1.639898	2.392615
H	4.375969	-1.447559	-3.145686
H	4.114554	-0.006915	-4.164165
H	2.767766	-0.662332	-3.191882
H	6.061762	1.256625	-1.127464
H	6.316999	-0.314686	-1.946189
H	2.489590	1.736443	-2.188188
H	3.865675	2.496220	-1.406098
H	3.802007	-3.548674	-1.537787
H	6.691905	-1.065005	0.480675
H	-3.007925	1.493584	-1.873706
H	-4.566660	2.297662	-2.232770
H	-4.378127	0.547318	-2.530234
H	-3.151808	2.459665	0.537358
H	-6.418717	0.096312	-1.131443
H	-6.408285	0.483358	0.611287
H	-3.015322	-3.844184	-1.397825
H	-4.201847	-1.907204	-2.590296
H	-6.130510	-2.923598	-3.807421
H	-8.155834	-3.655718	-2.541832
H	-8.236251	-3.385112	-0.060986
H	-6.299112	-2.379447	1.147383
H	5.601490	-5.176350	-2.123200
H	7.952086	-4.750958	-1.394623
H	8.496518	-2.692123	-0.089384
H	-4.742774	3.157294	0.141051
H	-6.592442	1.785626	-0.595913
H	0.165942	0.026932	-5.085909
H	-0.730658	1.120059	-3.948754
H	1.009410	1.019345	-3.848231
H	0.148307	-2.264377	-4.564483
H	0.800184	-2.688335	-2.943756
H	-0.951210	-2.649385	-3.203133
C	-0.913477	4.648942	0.827311

C	1.445652	4.484813	-1.653340
C	-1.842170	3.831894	-2.390394
C	2.712250	5.257439	-1.264603
H	1.507827	4.158056	-2.710824
H	0.587516	5.181025	-1.579450
C	-1.745976	4.913107	2.085934
H	0.153976	4.859663	1.022650
H	-1.238561	5.324927	0.008088
C	-2.494939	3.735653	-3.772689
H	-2.613086	3.870680	-1.600875
H	-1.279101	4.783067	-2.328641
H	-1.621906	5.955759	2.422124
H	-2.814292	4.726737	1.889040
H	-1.421678	4.230591	2.887796
H	2.842167	6.139394	-1.914786
H	2.635339	5.594457	-0.218734
H	3.606672	4.622492	-1.354381
H	-3.162204	4.596842	-3.946916
H	-1.722736	3.723216	-4.558981
H	-3.087987	2.811385	-3.854898

5.22 Transition State 24oa‡

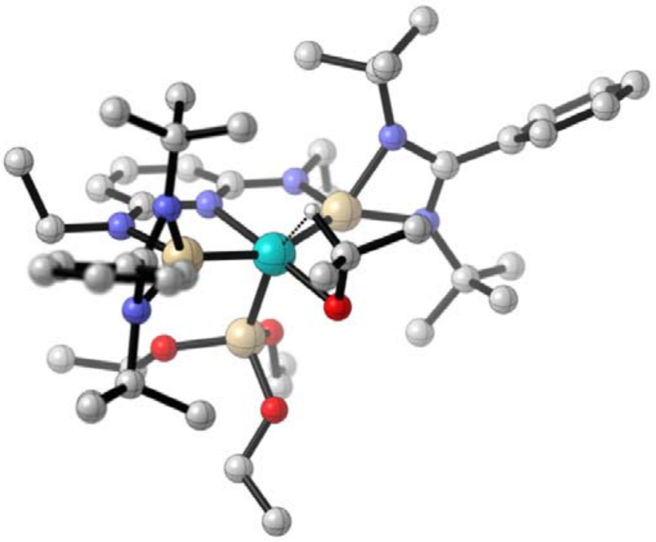

C	-1.112243	2.299103	3.550299
C	-1.108141	1.439788	2.425654

N	0.070437	1.054802	1.802996
C	1.259967	1.546769	2.323475
C	1.293146	2.426913	3.427478
C	0.095284	2.799591	4.032589
N	-2.277530	0.936209	1.889449
C	-3.573697	1.280037	2.490489
C	-3.869245	0.535615	3.806581
Fe	0.036409	0.160436	-0.176075
Si	-0.094448	2.311367	-0.793385
O	-1.367612	3.176508	-0.025604
C	-1.321139	4.546656	0.355895
C	-2.507463	4.861881	1.270664
N	2.418572	1.107835	1.719059
C	3.732716	1.382746	2.306041
C	4.312445	2.741395	1.877172
Si	2.181469	0.140276	0.250020
N	3.142554	-1.440126	0.767677
C	2.996919	-2.460659	1.823660
C	4.351589	-2.963595	2.369918
C	4.111851	-1.180182	-0.114158
N	3.868803	0.057049	-0.624393
C	4.341633	0.596034	-1.935222
C	5.856337	0.896857	-1.878976
C	5.212813	-2.107460	-0.482230
C	6.538461	-1.828376	-0.098518
C	7.559606	-2.749220	-0.372650
C	7.264656	-3.951801	-1.036040
C	5.943136	-4.232263	-1.423531
C	4.918272	-3.318729	-1.141001
C	3.582313	1.911853	-2.179192
C	4.018536	-0.376656	-3.089300
C	2.207614	-1.802166	2.980199
C	2.170339	-3.642912	1.267913
O	0.165669	-0.418955	-2.029469
C	0.020388	-1.710015	-1.597798
C	-1.275190	-2.337750	-2.158163
Si	-2.093294	0.046691	0.352628

N	-3.834471	0.044730	-0.441026
C	-4.029800	-1.244461	-0.081813
C	-5.120217	-2.149760	-0.532708
C	-4.863745	-3.132247	-1.507726
C	-5.891528	-3.994044	-1.919848
C	-7.173510	-3.884228	-1.356445
C	-7.429384	-2.904392	-0.381827
C	-6.407732	-2.036312	0.025923
C	1.232943	-2.607481	-1.943678
N	-3.014644	-1.580566	0.729203
C	-2.782870	-2.764601	1.583094
C	-2.191056	-3.932997	0.762716
C	-4.075057	-3.236732	2.285371
C	-1.738928	-2.337083	2.639214
C	-4.388351	0.839528	-1.570597
C	-4.395966	2.304056	-1.085064
C	-5.822758	0.433566	-1.966296
C	-3.456501	0.694619	-2.796559
O	-0.363700	2.520707	-2.461689
C	-1.226228	3.507259	-3.018739
C	-1.366877	3.247496	-4.521010
O	1.294941	3.257485	-0.444485
C	1.607020	4.439322	-1.178997
C	2.659678	5.247108	-0.415558
H	6.148435	1.442284	-2.790678
H	3.963079	2.377720	-3.101608
H	-0.060160	-1.736453	-0.419828
H	-2.047196	2.579890	4.025471
H	0.100879	3.483423	4.884538
H	2.240970	2.825870	3.777959
H	-4.346336	1.028208	1.751220
H	-3.631989	2.371371	2.641410
H	-3.974342	-0.542190	3.620026
H	-4.808775	0.905626	4.251368
H	-3.058299	0.677874	4.535527
H	4.410689	0.578038	1.980834
H	3.667327	1.318400	3.406561

H	4.427540	2.762322	0.784244
H	5.298225	2.909785	2.344643
H	3.637639	3.561826	2.159904
H	-5.086326	2.421093	-0.232894
H	-0.831794	-1.966967	2.136738
H	-1.471641	-3.203446	3.264516
H	-2.122835	-1.532062	3.280331
H	-2.031151	-4.801064	1.422888
H	-1.225737	-3.636357	0.331334
H	-4.573964	-2.399031	2.794991
H	-3.811365	-3.995923	3.039052
H	-4.781996	-3.685700	1.574908
H	1.201492	-3.279284	0.895317
H	1.990466	-4.381395	2.066598
H	2.702022	-4.138527	0.442735
H	4.895347	-3.575728	1.638499
H	4.161467	-3.579419	3.263500
H	4.987317	-2.112819	2.662379
H	2.797616	-1.004041	3.452193
H	1.969928	-2.567102	3.736239
H	1.269903	-1.362358	2.611513
H	4.555466	-1.329566	-2.977019
H	4.319484	0.079059	-4.047178
H	2.937199	-0.566875	-3.114877
H	6.087022	1.527830	-1.005216
H	6.454466	-0.022823	-1.825534
H	2.504629	1.733809	-2.292999
H	3.730197	2.604054	-1.339445
H	3.891184	-3.527609	-1.439622
H	6.757448	-0.896787	0.424938
H	-2.414738	0.943613	-2.548775
H	-3.802414	1.369111	-3.596975
H	-3.487052	-0.338378	-3.177428
H	-3.389238	2.617240	-0.775344
H	-5.865525	-0.569875	-2.411247
H	-6.505011	0.465702	-1.102709
H	-2.871750	-4.229668	-0.047669

H	-3.866951	-3.205394	-1.943364
H	-5.690217	-4.750014	-2.681698
H	-7.971509	-4.557372	-1.676625
H	-8.424806	-2.815971	0.058332
H	-6.595866	-1.273364	0.783091
H	5.710187	-5.163334	-1.944309
H	8.061111	-4.667614	-1.249738
H	8.584138	-2.529577	-0.065547
H	-4.735578	2.957005	-1.905125
H	-6.178323	1.157085	-2.716598
H	1.125903	-3.629619	-1.538429
H	1.314167	-2.668080	-3.041574
H	2.152151	-2.162471	-1.551264
H	-1.433614	-3.373457	-1.819523
H	-2.137642	-1.721678	-1.882559
H	-1.184531	-2.332386	-3.256831
H	1.985052	4.167420	-2.181822
H	0.711448	5.066857	-1.332637
H	-0.372690	4.758630	0.879962
H	-1.363690	5.197692	-0.540318
H	-2.217487	3.473730	-2.531886
H	-0.817448	4.524572	-2.855364
H	-2.502986	5.929549	1.549944
H	-3.458766	4.633437	0.763648
H	-2.439497	4.257053	2.187237
H	2.906211	6.174863	-0.959810
H	2.282322	5.510552	0.585792
H	3.579569	4.656219	-0.290614
H	-2.032127	3.997069	-4.984157
H	-0.380940	3.296618	-5.010086
H	-1.784871	2.243389	-4.690159

5.23 Intermediate 25oa

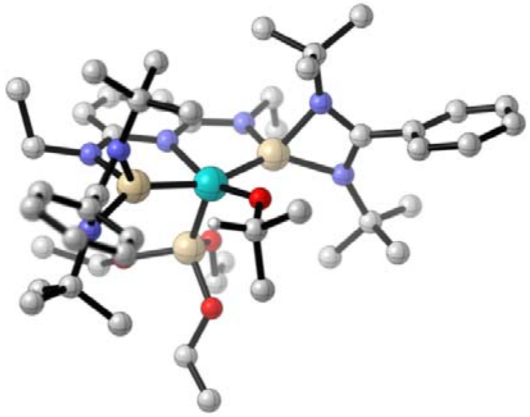

C	-6.055636	-2.627143	0.012786
C	-4.677157	-2.486873	0.255436
C	-3.965556	-3.524928	0.890249
C	-4.634760	-4.691852	1.282503
C	-6.012650	-4.831853	1.040350
C	-6.721005	-3.798859	0.405526
C	-3.934409	-1.272171	-0.170502
N	-3.796241	-0.105928	0.510347
C	-4.389134	0.273863	1.816820
C	-3.702667	1.574006	2.266314
N	-3.143577	-1.179533	-1.247667
C	-2.794693	-2.142824	-2.310451
C	-2.128054	-1.302506	-3.424350
Si	-2.245913	0.303244	-0.492451
N	-2.477467	1.573848	-1.699587
C	-3.778987	1.933153	-2.268599
C	-4.392016	3.185875	-1.619059
Fe	-0.116849	0.214306	-0.088393
O	-0.404293	-1.347152	0.839203
C	0.460328	-2.067703	1.689925
C	0.433478	-1.465397	3.102671
Si	2.028930	0.257914	-0.480432
N	2.995063	-1.239282	-1.171922
C	2.656434	-2.374910	-2.055063
C	3.879653	-2.819520	-2.885006
N	3.729312	0.073377	0.377894

C	4.382571	0.709928	1.547362
C	5.824235	0.216775	1.796888
C	3.914979	-1.132199	-0.199069
C	4.837822	-2.200030	0.271606
C	4.448741	-3.002472	1.362463
C	5.314050	-3.994158	1.846045
C	6.571924	-4.185418	1.249351
C	6.960457	-3.385207	0.162110
C	6.095682	-2.395334	-0.326632
C	3.510751	0.461732	2.797596
C	4.429016	2.221715	1.239587
N	2.233411	1.487799	-1.756667
C	1.072010	2.089064	-2.209812
N	-0.117891	1.602717	-1.682804
C	-1.304911	2.153115	-2.159051
C	-1.318575	3.214477	-3.088899
C	-0.108748	3.701552	-3.583051
C	1.093116	3.132395	-3.163687
C	3.534078	1.857442	-2.327978
C	3.763906	1.299905	-3.745185
Si	-0.038319	2.096279	1.088502
O	0.038320	1.823169	2.777887
O	-1.371421	3.152305	0.860159
O	1.307126	3.121902	0.773381
C	-1.789804	-3.194612	-1.785772
C	-4.053619	-2.828766	-2.881548
C	-4.186674	-0.790089	2.920507
C	-5.899471	0.538756	1.611800
C	1.554209	-1.859398	-3.005193
C	2.092509	-3.555208	-1.236974
C	0.003931	-3.543008	1.742195
H	-6.343066	0.910127	2.549730
H	-4.201699	1.945305	3.175472
H	2.038893	3.493480	-3.557549
H	-0.102113	4.524023	-4.301666
H	-2.262272	3.651229	-3.404682
H	4.303111	1.460056	-1.651194

H	3.648223	2.955824	-2.318721
H	3.768996	0.200872	-3.716113
H	4.733600	1.646110	-4.142120
H	2.967965	1.623432	-4.432803
H	-4.446213	1.070342	-2.111737
H	-3.682367	2.065874	-3.360977
H	-4.567314	3.001068	-0.548906
H	-5.352611	3.442657	-2.097612
H	-3.708926	4.043493	-1.702327
H	5.071204	2.412282	0.364569
H	0.691727	-1.506367	-2.421329
H	1.223592	-2.676183	-3.665703
H	1.919245	-1.023809	-3.620033
H	1.779282	-4.366050	-1.915049
H	1.221461	-3.210549	-0.668255
H	4.318651	-1.958068	-3.413033
H	3.557466	-3.561324	-3.633658
H	4.650041	-3.281870	-2.253419
H	-0.967181	-2.693188	-1.258468
H	-1.387680	-3.780333	-2.628815
H	-2.282729	-3.884752	-1.085860
H	-4.523501	-3.495703	-2.146527
H	-3.764907	-3.429126	-3.759212
H	-4.790192	-2.073321	-3.200339
H	-2.839900	-0.567498	-3.830245
H	-1.794425	-1.965899	-4.237503
H	-1.256286	-0.759885	-3.030938
H	-4.710664	-1.727216	2.694751
H	-4.603439	-0.391416	3.860527
H	-3.118921	-1.003969	3.088760
H	-6.048241	1.296797	0.826389
H	-6.425586	-0.382993	1.322623
H	-2.641136	1.403257	2.488496
H	-3.766705	2.342614	1.484958
H	-2.900709	-3.394397	1.085256
H	-6.597333	-1.826695	-0.493381
H	2.493159	0.837473	2.630003

H	3.944439	0.979422	3.669175
H	3.455637	-0.616076	3.017517
H	3.419419	2.600954	1.037813
H	5.863081	-0.812605	2.175174
H	6.424947	0.279663	0.875919
H	2.844731	-3.953010	-0.540313
H	3.471651	-2.841803	1.822494
H	5.007240	-4.615795	2.689861
H	7.246963	-4.954009	1.631259
H	7.938145	-3.528208	-0.302566
H	6.393327	-1.765271	-1.166326
H	-4.081281	-5.490453	1.780991
H	-6.531605	-5.742646	1.346184
H	-7.790579	-3.904671	0.212677
H	4.848187	2.759248	2.106017
H	6.279012	0.876706	2.552511
H	1.088156	-2.032640	3.790330
H	0.737489	-0.414351	3.078304
H	-0.600712	-1.504790	3.485811
H	0.731445	-4.159378	2.299137
H	-0.967437	-3.604369	2.262208
H	-0.116994	-3.967054	0.734406
C	1.274347	4.533194	0.576781
C	-1.714521	4.154084	1.814082
C	0.701816	2.691155	3.698772
C	-2.717707	5.124990	1.186008
H	-2.148404	3.682366	2.714670
H	-0.821773	4.717993	2.142223
C	2.451286	4.976556	-0.297716
H	0.325822	4.828941	0.096739
H	1.327431	5.046036	1.559218
C	0.875986	1.962836	5.034574
H	1.685122	2.995037	3.296213
H	0.107838	3.612862	3.848063
H	2.426022	6.071667	-0.433708
H	3.412067	4.703725	0.165231
H	2.382257	4.497129	-1.284637

H	-2.989195	5.918329	1.903388
H	-2.280372	5.591498	0.288401
H	-3.632515	4.593527	0.882943
H	1.363671	2.623045	5.772582
H	-0.104401	1.652705	5.429990
H	1.494161	1.061711	4.901881
H	1.503306	-2.024774	1.301952

5.24 Transition State 26oa‡

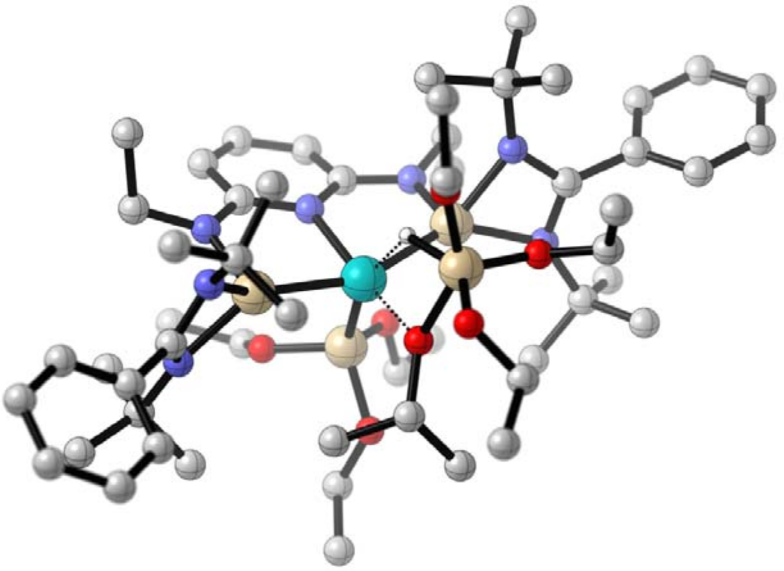

C	6.879304	-1.303275	-1.004918
C	5.823161	-1.236969	-0.078443
C	5.980860	-1.792392	1.204167
C	7.186343	-2.418944	1.553872
C	8.241402	-2.481024	0.628651
C	8.086993	-1.918680	-0.649996
C	4.533624	-0.584094	-0.440173
N	3.544232	-1.143624	-1.173030
C	3.360956	-2.605832	-1.383587
C	2.172954	-2.736251	-2.353491
N	4.140310	0.635124	-0.074301
C	4.903703	1.771324	0.505304
C	4.267017	2.169438	1.847164
Si	2.301502	0.275778	-0.688522

Fe	0.131993	0.276860	-0.064362
O	0.192691	-1.224573	1.430373
Si	-0.656295	-2.619548	0.498032
O	-0.653426	-3.515087	-0.959369
N	0.048447	1.649566	-1.740962
C	1.181058	1.977340	-2.460789
C	1.188731	3.017126	-3.422125
C	0.049898	3.799186	-3.585986
C	-1.101169	3.502302	-2.862587
C	-1.103541	2.376554	-2.003541
N	2.317877	1.243740	-2.193331
C	3.462284	1.317384	-3.113039
C	3.186257	0.633800	-4.462169
N	-2.269242	1.899196	-1.455888
Si	-2.102382	0.511911	-0.325158
N	-3.712820	0.495469	0.742123
C	-4.148960	0.911005	2.097568
C	-4.636117	-0.269245	2.973460
Si	0.276034	2.328932	1.020355
O	-1.092823	3.408149	0.932585
C	-1.287692	4.398807	1.948277
C	-2.527438	5.239828	1.641585
O	1.529368	3.352741	0.389662
C	1.347657	4.755848	0.159585
C	2.382624	5.326475	-0.813250
O	0.554698	2.292009	2.715658
C	1.302505	3.244969	3.471562
C	2.072288	2.548602	4.601933
C	-3.541282	2.457467	-1.932471
C	-3.884482	3.848570	-1.332846
N	-3.510849	-0.486919	-1.176897
C	-4.345389	-0.296739	-0.149161
C	-5.804444	-0.649314	-0.151041
C	-6.625790	0.088218	-1.032588
C	-8.007162	-0.139812	-1.090003
C	-8.596886	-1.108771	-0.263239
C	-7.794030	-1.832853	0.630620

C	-6.412706	-1.595455	0.696132
C	-3.590210	-1.356033	-2.380697
C	-3.936424	-2.822176	-2.030749
C	-4.635441	-0.797752	-3.381542
C	-2.202940	-1.289474	-3.056318
C	-5.265281	1.971699	1.966920
C	-2.912511	1.511645	2.781778
C	0.995787	-1.226927	2.657851
C	2.302313	-0.451235	2.519000
C	0.283371	-0.749875	3.944625
C	3.070950	-3.299862	-0.033162
C	4.589061	-3.266351	-2.055913
C	4.756650	2.925772	-0.508519
C	6.409390	1.519637	0.732668
O	0.127251	-3.794723	1.472720
O	-2.287377	-2.455813	0.857486
H	-5.611891	2.278533	2.967481
H	-3.213953	2.011863	3.715504
H	2.086902	3.231012	-3.993067
H	0.061114	4.645299	-4.276949
H	-1.997177	4.101946	-2.983100
H	4.308316	0.822932	-2.622686
H	3.766813	2.367970	-3.261176
H	2.977323	-0.433025	-4.299667
H	4.061554	0.728202	-5.127270
H	2.315795	1.085526	-4.960916
H	-4.319742	1.738496	-1.667051
H	-3.514855	2.503162	-3.035953
H	-4.737011	3.783318	-0.639398
H	-4.141683	4.578223	-2.118875
H	-3.021346	4.225709	-0.773377
H	5.254896	2.664558	-1.454678
H	1.288555	-2.205647	-1.975834
H	1.916990	-3.795550	-2.468179
H	2.445477	-2.332112	-3.340508
H	2.682567	-4.316975	-0.189881
H	2.329370	-2.736890	0.546325

H	4.920693	-2.670712	-2.921039
H	4.287572	-4.263103	-2.418394
H	5.431471	-3.398909	-1.367207
H	-3.029876	-3.334461	-1.691157
H	-4.325984	-3.329239	-2.929014
H	-4.705574	-2.879105	-1.245882
H	-5.661345	-0.934694	-3.014973
H	-4.539985	-1.339920	-4.336141
H	-4.456536	0.272380	-3.567533
H	-1.980277	-0.261822	-3.383571
H	-2.204141	-1.950332	-3.937635
H	-1.416898	-1.629242	-2.372792
H	-5.656433	-0.581674	2.722831
H	-4.638150	0.056457	4.026260
H	-3.946840	-1.120901	2.871636
H	-4.883287	2.858647	1.439442
H	-6.122371	1.566033	1.407134
H	-2.181754	0.725910	3.018393
H	-2.432437	2.229684	2.114762
H	-5.819872	-2.106508	1.451069
H	-6.176285	0.837327	-1.683013
H	3.206818	2.398493	1.710672
H	4.770999	3.070657	2.233516
H	4.381383	1.359213	2.581295
H	3.695915	3.112298	-0.698277
H	6.598443	0.770083	1.512937
H	6.917735	1.202391	-0.188217
H	3.996635	-3.383021	0.556661
H	5.158830	-1.730730	1.918981
H	7.302773	-2.854346	2.548472
H	9.181125	-2.964830	0.902865
H	8.905660	-1.963528	-1.371129
H	6.744869	-0.876463	-2.000162
H	-8.242636	-2.572703	1.296563
H	-9.672907	-1.288616	-0.306512
H	-8.620984	0.445815	-1.777348
H	5.220096	3.841136	-0.106528

H	6.849809	2.475750	1.056739
H	2.911409	-0.655637	3.418515
H	2.876333	-0.798670	1.653839
H	2.131093	0.622323	2.448863
H	0.957627	-0.967724	4.790208
H	0.109676	0.332577	3.892129
H	-0.678015	-1.238260	4.167892
H	-1.406020	3.910282	2.931489
H	-0.410760	5.063619	2.025644
H	0.337952	4.937919	-0.242020
H	1.430908	5.301555	1.120867
H	1.997302	3.798094	2.816334
H	0.605008	3.983576	3.911040
H	2.130594	6.381076	-1.021046
H	3.398326	5.291022	-0.394037
H	2.365142	4.769490	-1.761708
H	-2.704940	5.957237	2.461148
H	-2.399211	5.804130	0.704220
H	-3.414643	4.597323	1.540372
H	2.424395	3.295014	5.334691
H	1.411655	1.834820	5.117731
H	2.941590	1.997845	4.216934
H	1.322589	-2.259357	2.807440
H	-0.448068	-1.301060	-0.480398
C	-3.200439	-3.461313	1.295150
C	-0.350525	-3.936612	2.827565
C	-0.206495	-4.859560	-1.110320
H	-4.199275	-3.076481	1.081294
C	-3.073453	-4.858902	0.674558
H	-3.115613	-3.604122	2.383707
C	-0.594852	-5.356135	-2.504440
H	0.879880	-4.920963	-0.953572
H	-0.653254	-5.520454	-0.351593
C	0.741640	-4.492246	3.736403
H	-0.784761	-2.996653	3.179048
H	-1.195598	-4.647136	2.847416
H	0.347457	-4.636203	4.756343

H	1.080192	-5.465035	3.346409
H	1.616926	-3.827544	3.793444
H	-0.258357	-6.397504	-2.645873
H	-1.690062	-5.321442	-2.625030
H	-0.150183	-4.731039	-3.292647
H	-3.866229	-5.491270	1.112492
H	-3.188488	-4.853201	-0.414625
H	-2.110143	-5.322332	0.933435

5.25 Transition State 27oa‡

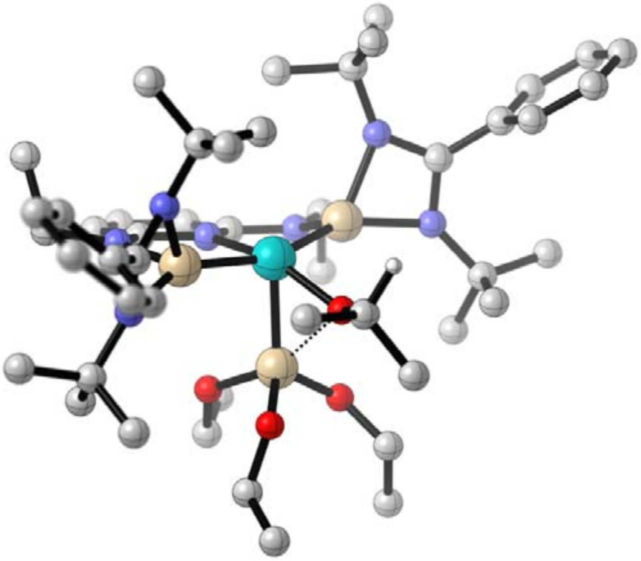

C	4.795187	-2.652740	-2.058100
C	5.197294	-2.011293	-0.869722
C	6.508825	-2.184956	-0.391272
C	7.412699	-2.992761	-1.095940
C	7.011505	-3.630547	-2.281720
C	5.700984	-3.460720	-2.759963
C	4.226570	-1.134777	-0.161291
N	3.384042	-1.503336	0.815770
C	3.195666	-2.788844	1.508766
C	2.140746	-2.518686	2.606132
Si	2.282056	0.031806	0.489012
N	3.911388	0.139746	-0.480447
C	4.389763	1.003393	-1.586549

C	4.262871	2.454425	-1.076396
Fe	0.080748	0.079980	0.157580
Si	-0.294312	2.116414	-0.598521
O	-1.518725	2.487084	-1.758052
C	-2.057174	3.804138	-1.842959
C	-2.609647	4.026906	-3.255263
Si	-2.010376	-0.160534	0.653156
N	-2.111111	0.083671	2.410735
C	-3.301485	-0.180894	3.226006
C	-3.142929	-1.396608	4.156905
N	-2.876758	-1.863397	0.334647
C	-2.355290	-3.219703	0.047154
C	-2.696681	-4.143577	1.239749
C	-3.977980	-1.235027	-0.119716
N	-3.804540	0.079689	0.104483
C	-4.759257	1.213006	0.038833
C	-6.148843	0.822753	0.588434
C	-5.145994	-1.878067	-0.766887
C	-5.967135	-2.739652	-0.013152
C	-7.060446	-3.375074	-0.617905
C	-7.330615	-3.163998	-1.980832
C	-6.506419	-2.311692	-2.735641
C	-5.418575	-1.665722	-2.132068
C	-0.817856	-3.088430	-0.059008
C	-2.884692	-3.856471	-1.259492
C	-4.881205	1.708539	-1.418588
C	-4.168731	2.339691	0.914017
N	0.192749	0.523858	2.205557
C	-0.944140	0.545721	3.001607
C	-0.933299	1.002292	4.337498
C	0.261240	1.451220	4.895708
C	1.421944	1.445088	4.124090
C	1.366571	0.992898	2.787517
N	2.507999	0.940997	1.999893
C	3.827046	1.223696	2.581319
C	4.129054	2.729000	2.701369
C	3.470849	0.770355	-2.805799

C	5.864522	0.750177	-1.968630
O	1.076802	3.007665	-1.108285
C	1.267913	3.576562	-2.398152
C	1.577981	5.073613	-2.278261
O	-0.839065	3.069160	0.742318
C	-0.429170	4.387431	1.076997
C	0.771476	4.378328	2.029951
C	2.684840	-3.868602	0.528061
C	4.505974	-3.254216	2.181658
C	-1.884647	-0.504682	-2.821200
C	-0.380225	-0.196661	-2.912935
C	-0.097316	0.694495	-4.137009
H	6.174190	1.549953	-2.659974
H	4.535515	3.152738	-1.883486
H	-1.849325	1.008179	4.921395
H	0.288104	1.809139	5.927226
H	2.358927	1.795905	4.544914
H	-4.131273	-0.361825	2.530235
H	-3.573848	0.718230	3.808747
H	-2.952278	-2.298758	3.558068
H	-4.062810	-1.551421	4.746211
H	-2.300936	-1.254052	4.850186
H	4.575628	0.762868	1.920247
H	3.920477	0.728925	3.566316
H	4.073215	3.199952	1.710046
H	5.142132	2.881341	3.111543
H	3.404356	3.237003	3.352807
H	-4.142981	2.037822	1.970632
H	-0.530818	-2.484902	-0.934587
H	-0.364574	-4.088128	-0.146958
H	-0.414989	-2.602969	0.845157
H	-2.309200	-4.776763	-1.450118
H	-2.749323	-3.178758	-2.113776
H	-2.233641	-3.764759	2.163139
H	-2.319170	-5.161620	1.048993
H	-3.786873	-4.196000	1.384014
H	1.814642	-3.485945	-0.024001

H	2.381898	-4.771353	1.084038
H	3.469483	-4.145789	-0.191323
H	5.275800	-3.492695	1.435547
H	4.307803	-4.161683	2.774766
H	4.887055	-2.470339	2.855466
H	2.485351	-1.721610	3.282156
H	1.968467	-3.435405	3.191458
H	1.189622	-2.189327	2.161861
H	3.633804	-0.242655	-3.209251
H	3.699264	1.500589	-3.599676
H	2.415893	0.867694	-2.510948
H	6.513789	0.791266	-1.079548
H	6.014129	-0.212327	-2.473679
H	3.232992	2.669666	-0.760379
H	4.948650	2.612833	-0.229543
H	3.777080	-2.511818	-2.424593
H	6.816768	-1.676972	0.524000
H	-3.878362	1.879269	-1.827281
H	-5.447853	2.653714	-1.444426
H	-5.411318	0.971380	-2.038755
H	-3.145028	2.595254	0.606674
H	-6.641203	0.065867	-0.036681
H	-6.066356	0.437369	1.617450
H	-3.946381	-4.126445	-1.187883
H	-4.756642	-1.024832	-2.714390
H	-6.707380	-2.155099	-3.797436
H	-8.178643	-3.663875	-2.453345
H	-7.699281	-4.035286	-0.027723
H	-5.746944	-2.896658	1.044130
H	5.384736	-3.955602	-3.680556
H	7.717157	-4.256665	-2.831451
H	8.430732	-3.120081	-0.722265
H	-4.804323	3.234401	0.821145
H	-6.785300	1.721951	0.606887
H	-2.218982	-1.020951	-3.739348
H	-2.086686	-1.158581	-1.966202
H	-2.449294	0.427492	-2.697134

H	-0.396152	0.180209	-5.067140
H	-0.668439	1.628719	-4.041187
H	0.973222	0.939536	-4.196468
H	2.121581	3.072824	-2.885531
H	0.385977	3.412292	-3.037443
H	-0.167387	4.968089	0.174286
H	-1.291985	4.888310	1.556953
H	-2.856433	3.941369	-1.092140
H	-1.285655	4.566232	-1.634499
H	1.039794	5.407269	2.331867
H	0.541524	3.787587	2.929512
H	1.632539	3.918665	1.525061
H	1.791508	5.503290	-3.272843
H	0.728913	5.618185	-1.835906
H	2.457017	5.227572	-1.631482
H	-3.052426	5.034031	-3.341839
H	-1.802681	3.931467	-3.999390
H	-3.384831	3.281639	-3.493443
H	0.149102	-1.165802	-3.058538
O	0.178435	0.417094	-1.761617

5.26 Intermediate 28

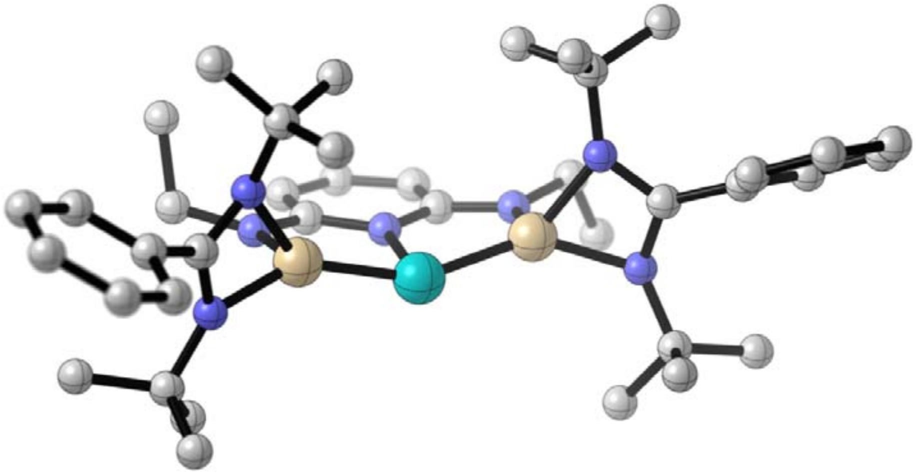

C	-4.885009	-3.087245	0.140055
C	-5.190608	-1.708690	0.120317
C	-6.537398	-1.301456	0.014092

C	-7.558751	-2.255820	-0.076906
C	-7.249567	-3.627774	-0.054456
C	-5.910025	-4.039378	0.059624
C	-4.099179	-0.708959	0.156747
N	-3.135848	-0.561405	-0.766674
C	-3.032547	-1.000956	-2.173613
C	-2.163850	0.050625	-2.903656
Si	-2.096535	0.548396	0.371227
N	-3.813167	0.171424	1.154756
C	-4.187579	0.063264	2.588385
C	-3.452141	1.207608	3.320039
Fe	0.004704	0.605883	0.857053
N	-0.005711	2.257905	-0.235451
C	1.180498	2.873238	-0.650048
C	1.188915	4.147062	-1.260057
C	-0.018522	4.801495	-1.505025
C	-1.223258	4.171692	-1.189832
C	-1.201886	2.891206	-0.594957
N	2.345786	2.152789	-0.438861
C	3.605886	2.590313	-1.047498
C	3.647865	2.374314	-2.572106
N	-2.359459	2.169117	-0.365244
C	-3.650691	2.658104	-0.850411
C	-4.309475	3.664989	0.110674
Si	2.101556	0.559749	0.365112
N	3.833868	0.217387	1.093401
C	4.628623	0.724920	2.228548
C	6.100742	0.971317	1.830549
C	4.105915	-0.708330	0.155967
N	3.123461	-0.625601	-0.765467
C	2.672229	-1.646120	-1.741580
C	3.779595	-1.995716	-2.759711
C	5.228685	-1.681351	0.182485
C	6.330455	-1.516495	-0.681414
C	7.398984	-2.422423	-0.642599
C	7.373487	-3.506169	0.252559
C	6.273711	-3.678974	1.109543

C	5.206952	-2.768794	1.078941
C	1.474022	-1.021561	-2.488676
C	2.199885	-2.919569	-1.002503
C	4.555901	-0.263112	3.416373
C	3.982381	2.064930	2.644293
C	-3.726808	-1.293612	3.169847
C	-5.707931	0.245990	2.797249
C	-2.319849	-2.372858	-2.237824
C	-4.411756	-1.079165	-2.862609
H	-5.914443	0.352484	3.874787
H	-3.676240	1.158385	4.397228
H	2.131181	4.609264	-1.542931
H	-0.021286	5.794943	-1.959332
H	-2.172302	4.655709	-1.404682
H	4.407124	2.004625	-0.574806
H	3.804338	3.648944	-0.799017
H	3.537980	1.301265	-2.787380
H	4.606338	2.726674	-2.990971
H	2.827105	2.917525	-3.064427
H	-4.307919	1.782581	-0.960711
H	-3.535690	3.100670	-1.857417
H	-4.504859	3.174529	1.076014
H	-5.266073	4.030906	-0.300849
H	-3.649416	4.527576	0.288581
H	4.018199	2.786532	1.815256
H	0.694893	-0.731637	-1.763348
H	1.055125	-1.755307	-3.195344
H	1.783396	-0.125016	-3.047612
H	1.798278	-3.649693	-1.724925
H	1.407469	-2.653383	-0.284331
H	4.194352	-1.077667	-3.205538
H	3.344054	-2.608511	-3.565774
H	4.595783	-2.566946	-2.297273
H	-1.353226	-2.317276	-1.715134
H	-2.141032	-2.651935	-3.289762
H	-2.937944	-3.154071	-1.771545
H	-5.033674	-1.884583	-2.449920

H	-4.260636	-1.271212	-3.937009
H	-4.949365	-0.123906	-2.751430
H	-2.672426	1.025011	-2.932992
H	-1.975550	-0.286124	-3.935416
H	-1.199139	0.178381	-2.390830
H	-4.247857	-2.127180	2.675163
H	-3.942660	-1.338402	4.250252
H	-2.641265	-1.411115	3.019680
H	-6.055973	1.156075	2.283193
H	-6.276408	-0.615557	2.423306
H	-2.361959	1.121879	3.177366
H	-3.775953	2.186640	2.934995
H	-3.844097	-3.399724	0.233499
H	-6.770447	-0.235993	-0.007693
H	3.502925	-0.471966	3.662651
H	5.047783	0.175549	4.300514
H	5.061761	-1.207759	3.170621
H	2.926790	1.910978	2.920137
H	6.615506	0.033988	1.579511
H	6.155941	1.649564	0.964171
H	3.037304	-3.388418	-0.462944
H	4.348345	-2.899182	1.739071
H	6.245110	-4.523176	1.801728
H	8.205481	-4.212760	0.280370
H	8.250809	-2.284094	-1.311740
H	6.340892	-0.676192	-1.377377
H	-5.664005	-5.103131	0.087373
H	-8.047064	-4.370261	-0.124515
H	-8.597105	-1.929951	-0.168044
H	4.520643	2.481480	3.509961
H	6.628560	1.442442	2.675381

5.27 Intermediate 29

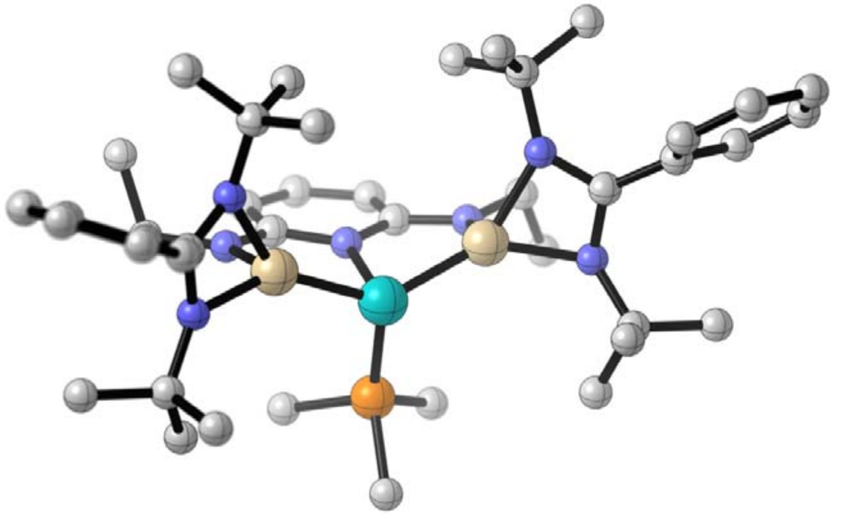

C	4.816924	2.972828	-1.476562
C	5.025306	1.980192	-0.499246
C	6.240323	1.953793	0.214274
C	7.233376	2.907359	-0.047152
C	7.021200	3.897579	-1.022172
C	5.812058	3.927180	-1.736152
C	3.963281	0.983784	-0.187967
N	2.957910	1.146551	0.675490
C	2.521079	2.333670	1.434684
C	1.852035	3.348533	0.475984
N	3.800197	-0.232015	-0.760734
C	4.517998	-0.820599	-1.909601
C	4.024130	-2.275598	-2.028132
Si	2.017965	-0.422950	-0.041605
N	2.278387	-1.585672	1.341076
C	3.570919	-1.867878	1.972484
C	3.824879	-1.055742	3.257434
C	1.125299	-2.140865	1.854750
N	-0.046282	-1.821895	1.177905
C	-1.261434	-2.201921	1.743069
C	-1.311158	-3.078381	2.850763
C	-0.115867	-3.479020	3.453586
C	1.106464	-2.992014	2.984936

Fe	0.012065	-0.996600	-0.638531
Si	-2.004629	-0.398483	-0.117378
N	-2.378591	-1.648902	1.159516
C	-3.707024	-1.908580	1.712607
C	-4.305431	-3.238638	1.219354
N	-2.985762	1.070860	0.704693
C	-2.849732	1.871758	1.937027
C	-4.208585	2.304230	2.530452
C	-3.928517	1.032676	-0.235674
N	-3.713089	-0.094033	-0.976658
C	-4.063848	-0.259104	-2.413725
C	-5.590303	-0.221980	-2.655048
C	-4.965636	2.070141	-0.458842
C	-4.591039	3.367285	-0.867490
C	-5.560326	4.369726	-1.012518
C	-6.910145	4.091357	-0.736928
C	-7.287568	2.800042	-0.327158
C	-6.322893	1.793426	-0.194055
C	-2.123251	0.979356	2.972603
C	-1.981489	3.116152	1.634248
C	-3.542330	-1.645703	-2.836754
C	-3.363800	0.829779	-3.260423
C	6.048584	-0.831302	-1.695421
C	4.164908	-0.062004	-3.211211
C	3.678902	3.007646	2.202681
C	1.464573	1.827401	2.440370
H	-5.796524	-0.556280	-3.685157
H	-3.746100	-1.807774	-3.907178
H	2.034069	-3.251739	3.488546
H	-0.139159	-4.151059	4.314636
H	-2.269750	-3.416048	3.236597
H	4.348014	-1.626255	1.232353
H	3.652867	-2.950063	2.180006
H	3.842745	0.015930	3.014594
H	4.793314	-1.334830	3.707835
H	3.029302	-1.232806	3.997245
H	-4.357584	-1.080486	1.396310

H	-3.677043	-1.887571	2.818565
H	-4.392020	-3.220445	0.122416
H	-5.307178	-3.400320	1.653441
H	-3.661255	-4.086445	1.498066
H	4.268203	-2.840742	-1.115317
H	0.661473	1.311091	1.896290
H	1.034072	2.678516	2.990041
H	1.909396	1.123968	3.158582
H	1.418362	4.183084	1.051998
H	1.051518	2.845748	-0.088913
H	4.213752	2.269516	2.821090
H	3.261290	3.780756	2.868201
H	4.397760	3.488089	1.525660
H	-1.015957	2.809182	1.208956
H	-1.797778	3.681693	2.563236
H	-2.495920	3.776239	0.919601
H	-4.710788	3.060374	1.913018
H	-4.033663	2.732673	3.530543
H	-4.875645	1.434109	2.635559
H	-2.764821	0.142626	3.282459
H	-1.870749	1.581440	3.859817
H	-1.199288	0.561261	2.552726
H	-3.735266	1.829355	-2.986973
H	-3.558006	0.667529	-4.333861
H	-2.276540	0.793001	-3.083793
H	-6.102564	-0.904071	-1.958092
H	-6.003366	0.787116	-2.534052
H	-2.458858	-1.716977	-2.656054
H	-4.045715	-2.435360	-2.257164
H	-3.540783	3.577284	-1.074708
H	-6.608284	0.789263	0.123183
H	3.071183	-0.040536	-3.341476
H	4.617900	-0.566507	-4.081025
H	4.541483	0.970529	-3.174516
H	2.933408	-2.291456	-2.166119
H	6.475086	0.179550	-1.726479
H	6.294325	-1.290904	-0.724580

H	2.588965	3.760334	-0.230004
H	3.876130	2.991598	-2.028433
H	5.642473	4.693151	-2.495912
H	7.794367	4.641738	-1.224002
H	8.171401	2.880123	0.511333
H	6.395421	1.184789	0.972953
H	-5.262111	5.368592	-1.338433
H	-7.663542	4.874854	-0.841102
H	-8.334828	2.578756	-0.110446
H	4.505850	-2.764981	-2.889361
H	6.518738	-1.428950	-2.493373
P	0.008311	-3.029955	-1.423527
C	0.270552	-3.347128	-3.261837
C	1.250371	-4.233567	-0.682370
C	-1.494079	-4.124385	-1.127484
H	1.159391	-5.231582	-1.145909
H	2.275655	-3.862402	-0.797749
H	1.037590	-4.320570	0.394019
H	0.244684	-4.427373	-3.489131
H	-0.521711	-2.843707	-3.839827
H	1.243650	-2.938323	-3.577934
H	-1.313542	-5.160396	-1.465870
H	-1.700388	-4.130007	-0.047524
H	-2.373795	-3.719034	-1.642248

5.28 Transition State 30oa‡

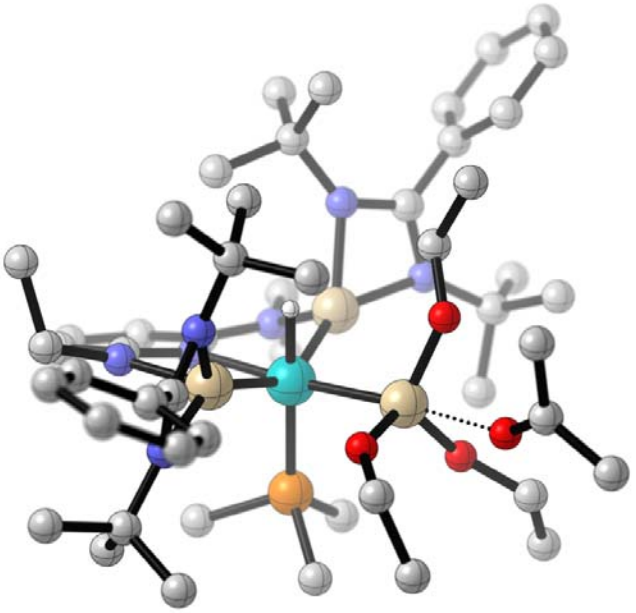

C	-6.107929	-1.074556	-2.106190
C	-5.344975	-0.091666	-1.444509
C	-5.866994	1.206921	-1.306929
C	-7.135038	1.516924	-1.823662
C	-7.893487	0.532408	-2.474165
C	-7.374794	-0.765519	-2.616035
C	-4.025122	-0.469998	-0.856843
N	-2.852086	-0.566026	-1.509353
C	-2.520759	-0.058981	-2.860627
C	-1.113862	-0.573606	-3.222081
N	-3.812024	-0.804471	0.418334
C	-4.766925	-1.093986	1.506679
C	-5.230318	0.217955	2.181883
Si	-1.874169	-0.983052	0.108743
Fe	0.061049	-0.449193	0.824232
Si	-0.172912	1.945977	0.627896
O	0.703530	2.584878	1.960291
C	0.874835	3.868890	2.537892
C	1.567324	3.764295	3.903985
N	0.323585	-2.567848	0.861311
C	-0.719825	-3.407631	0.556614

C	-0.639494	-4.801407	0.803209
C	0.537187	-5.321639	1.337278
C	1.641687	-4.494305	1.559180
C	1.527770	-3.117832	1.249597
N	-1.842351	-2.809291	0.017348
C	-2.897814	-3.652324	-0.548160
C	-2.480906	-4.320900	-1.870445
N	2.598197	-2.240197	1.272273
Si	2.155976	-0.659753	0.437091
N	3.895347	0.230934	0.379209
C	4.628761	1.249837	1.155014
C	4.466734	2.655460	0.524876
P	-0.078762	-0.453562	3.025668
C	1.376153	-0.229931	4.186116
C	-0.576632	-2.123202	3.744065
C	-1.303071	0.676935	3.882686
C	3.951661	-2.782642	1.423726
C	4.355820	-2.977960	2.898112
N	3.091948	-0.977201	-1.228833
C	4.117624	-0.193761	-0.878438
C	5.261652	0.193361	-1.751420
C	6.459693	-0.544308	-1.737986
C	7.528040	-0.166911	-2.562849
C	7.409973	0.952982	-3.402227
C	6.218369	1.694769	-3.412682
C	5.146423	1.317273	-2.590394
C	2.908638	-1.852028	-2.406352
C	2.663068	-1.004891	-3.676154
C	4.129374	-2.777442	-2.614848
C	1.673752	-2.740349	-2.122474
C	6.122608	0.886934	1.291973
C	3.971290	1.270525	2.544423
O	0.450919	2.452710	-0.896126
C	1.375668	1.790966	-1.729743
C	1.432238	2.514480	-3.082981
O	-1.919082	2.036035	0.627184
C	-3.017417	2.901197	0.815515

C	-3.076247	3.591187	2.186968
C	-2.490320	1.487790	-2.816545
C	-3.494077	-0.557297	-3.954523
C	-3.995723	-1.943237	2.535456
C	-5.997575	-1.899693	1.034057
O	-0.580260	4.140049	0.512194
C	-0.924018	5.094174	-0.188283
C	-1.189966	6.460655	0.447406
C	-1.122386	5.044214	-1.693615
H	6.587172	1.554971	2.036308
H	4.443215	2.040866	3.175643
H	-0.078355	-0.320815	-0.694919
H	-1.485177	-5.451687	0.591762
H	0.603752	-6.388285	1.567762
H	2.574438	-4.904639	1.936774
H	-3.769816	-3.011418	-0.729706
H	-3.224192	-4.414216	0.185681
H	-2.230402	-3.542896	-2.608043
H	-3.301738	-4.940241	-2.272505
H	-1.595603	-4.957721	-1.723016
H	4.644387	-2.065478	0.958776
H	4.050972	-3.737979	0.873761
H	4.317383	-2.014515	3.427421
H	5.382026	-3.377931	2.973045
H	3.669095	-3.670454	3.409154
H	-3.697559	-2.908001	2.098089
H	-0.380010	-0.233433	-2.485507
H	-0.835494	-0.184803	-4.214405
H	-1.090964	-1.673585	-3.255672
H	-2.193353	1.896120	-3.798145
H	-1.772788	1.810407	-2.049349
H	-3.616241	-1.650574	-3.890977
H	-3.062772	-0.312807	-4.939311
H	-4.480062	-0.080764	-3.890346
H	1.809977	-0.330093	-3.523084
H	2.437428	-1.667604	-4.528281
H	3.552442	-0.405022	-3.922573

H	5.007551	-2.225536	-2.977326
H	3.874723	-3.550129	-3.359084
H	4.381109	-3.279875	-1.667184
H	1.902257	-3.471088	-1.333399
H	1.402759	-3.286691	-3.039868
H	0.818399	-2.138795	-1.788131
H	5.022775	2.735794	-0.422234
H	4.854545	3.417265	1.222010
H	3.400879	2.861943	0.348868
H	6.231319	-0.152085	1.641827
H	6.658380	1.003640	0.339969
H	2.905467	1.500271	2.428872
H	4.074771	0.295709	3.039931
H	4.219512	1.889494	-2.590324
H	6.546379	-1.412260	-1.082318
H	1.750669	0.798187	4.137807
H	2.169215	-0.917697	3.860341
H	1.089246	-0.464750	5.226382
H	0.277651	-2.806065	3.637186
H	-1.411508	-2.558725	3.184326
H	-0.841157	-2.037670	4.813486
H	-4.360375	0.779873	2.547043
H	-5.888061	-0.016103	3.035093
H	-5.788872	0.845963	1.470869
H	-3.092753	-1.413434	2.849641
H	-6.685962	-1.292016	0.433998
H	-5.680553	-2.774082	0.444833
H	-3.484727	1.883147	-2.558158
H	-5.274039	1.974657	-0.812702
H	-7.528884	2.530235	-1.715758
H	-8.882255	0.773853	-2.870764
H	-7.957242	-1.537118	-3.124728
H	-5.698570	-2.080151	-2.213079
H	-1.296337	0.538160	4.978886
H	-2.314565	0.491471	3.499842
H	-1.021553	1.706300	3.628535
H	6.119607	2.567963	-4.061861

H	8.244852	1.247575	-4.041867
H	8.453427	-0.747197	-2.549261
H	-4.624751	-2.122660	3.421419
H	-6.543122	-2.260877	1.921380
H	-3.909035	2.262498	0.727177
H	-3.102513	3.668550	0.012566
H	1.467450	4.525450	1.870179
H	-0.101261	4.363094	2.690215
H	2.380507	1.774586	-1.272720
H	1.086408	0.743083	-1.886679
H	2.127407	2.003411	-3.767507
H	0.434396	2.513437	-3.549106
H	1.753180	3.560569	-2.954964
H	-4.000542	4.189116	2.275489
H	-3.062604	2.837949	2.984868
H	-2.211071	4.253068	2.325825
H	1.672858	4.773297	4.340642
H	0.961102	3.152751	4.587279
H	2.567289	3.317385	3.816903
H	-1.030142	6.417539	1.531418
H	-0.520960	7.209588	-0.007144
H	-2.220346	6.781144	0.225070
H	-0.913027	4.040567	-2.066692
H	-2.148774	5.369962	-1.927389
H	-0.442208	5.775709	-2.161038

5.29 Intermediate 31oa

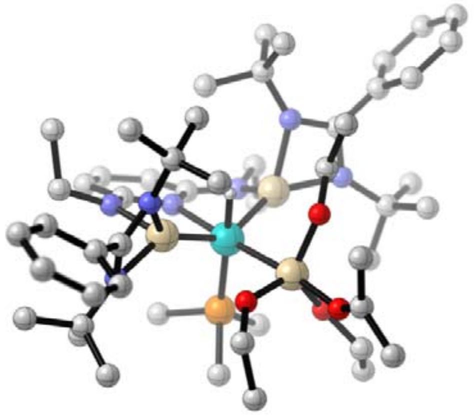

C	-1.535524	-3.013340	-1.399260
N	-0.330793	-2.481993	-0.989906
C	0.707922	-3.340297	-0.727645
C	0.631610	-4.714868	-1.062575
C	-0.544984	-5.204133	-1.625604
C	-1.651982	-4.366345	-1.789200
Fe	-0.054318	-0.355493	-0.824618
Si	0.201138	2.043950	-0.535935
O	-0.514959	2.143989	1.053114
C	-1.583393	1.425708	1.609496
C	-1.680277	1.785149	3.098851
N	1.824361	-2.776690	-0.141775
C	2.872050	-3.658079	0.380871
C	2.440003	-4.414855	1.648939
N	-2.609103	-2.142571	-1.356546
C	-3.956935	-2.693664	-1.522074
C	-4.380871	-2.797973	-2.999034
Si	-2.158130	-0.614173	-0.432209
N	-3.897055	0.260257	-0.328310
C	-4.635688	1.318216	-1.049282
C	-6.125557	0.945000	-1.227891
N	-3.092216	-1.036950	1.205701
C	-2.913636	-1.983582	2.324474
C	-4.135230	-2.919912	2.472251
C	-4.123285	-0.242486	0.900763
C	-5.274297	0.082109	1.790228

C	-5.180660	1.161857	2.686556
C	-6.265969	1.487235	3.512277
C	-7.448452	0.734812	3.450401
C	-7.542652	-0.345839	2.558673
C	-6.461591	-0.669701	1.729432
C	-1.680420	-2.849353	1.982560
C	-2.676692	-1.219550	3.647659
C	-3.966308	1.425968	-2.429926
C	-4.503502	2.685899	-0.337240
P	0.052784	-0.260619	-3.025028
C	0.546531	-1.900977	-3.815142
Si	1.873878	-0.950584	-0.118479
N	3.806099	-0.756350	-0.432728
C	4.740541	-0.955845	-1.557369
C	3.940907	-1.685066	-2.652986
C	1.278518	0.874978	-3.876053
C	-1.429557	-0.054815	-4.152198
N	2.863819	-0.657719	1.516161
C	4.033508	-0.531813	0.862447
C	5.355965	-0.201912	1.464653
C	6.121915	-1.223716	2.060664
C	7.398248	-0.949140	2.564725
C	7.923507	0.351187	2.481266
C	7.160965	1.373554	1.897202
C	5.882690	1.099639	1.388907
C	2.578156	-0.270924	2.916544
C	2.608555	1.269851	3.022660
C	3.566441	-0.911187	3.920138
C	1.166349	-0.769256	3.273561
C	5.969902	-1.812425	-1.180976
C	5.212240	0.404806	-2.116539
O	1.981480	2.027073	-0.535879
C	3.074788	2.884201	-0.794489
C	3.101537	3.480234	-2.208518
O	-0.785901	2.429274	-1.941522
C	-1.058489	3.607776	-2.694367
C	-1.797993	3.234524	-3.982670

O	0.607601	3.926938	-0.396113
C	0.970068	4.855575	0.359038
C	1.295830	6.218333	-0.229980
C	1.102160	4.734556	1.858601
H	-6.585597	1.648516	-1.942026
H	-4.441966	2.227905	-3.017925
H	0.092115	-0.301679	0.699391
H	1.479810	-5.374530	-0.894141
H	-0.608242	-6.253025	-1.926983
H	-2.588227	-4.750746	-2.186995
H	3.743084	-3.033113	0.613431
H	3.205842	-4.370498	-0.397507
H	2.179725	-3.691885	2.436348
H	3.257693	-5.059743	2.016315
H	1.558268	-5.041654	1.447049
H	-4.656885	-2.029744	-0.996409
H	-4.030483	-3.687594	-1.039351
H	-4.367639	-1.802431	-3.466932
H	-5.400065	-3.213764	-3.085374
H	-3.688775	-3.444617	-3.561465
H	3.632427	-2.687815	-2.318285
H	0.422563	-0.338469	2.596156
H	0.929585	-0.465585	4.305759
H	1.105714	-1.864756	3.206720
H	2.357868	1.590221	4.048830
H	1.875960	1.688721	2.317603
H	3.639321	-1.996689	3.746086
H	3.181854	-0.747407	4.940446
H	4.569834	-0.469110	3.861299
H	-1.831385	-0.526904	3.542412
H	-2.447071	-1.935112	4.455200
H	-3.573237	-0.647061	3.931338
H	-5.012224	-2.390855	2.871316
H	-3.879801	-3.737631	3.166771
H	-4.389639	-3.360404	1.495162
H	-1.908044	-3.528563	1.147944
H	-1.405732	-3.452940	2.862268

H	-0.828291	-2.224748	1.687667
H	-5.091864	2.711375	0.592644
H	-4.874226	3.481560	-1.004699
H	-3.446894	2.890678	-0.110613
H	-6.215268	-0.073832	-1.637551
H	-6.680296	1.003115	-0.281872
H	-2.900397	1.656732	-2.298771
H	-4.061879	0.482348	-2.983952
H	-4.261487	1.745879	2.724059
H	-6.531456	-1.504595	1.029630
H	-1.834240	0.961053	-4.066518
H	-2.194628	-0.780188	-3.841384
H	-1.146557	-0.246507	-5.202809
H	-0.296417	-2.600673	-3.726872
H	1.400959	-2.349293	-3.297484
H	0.789186	-1.762264	-4.885150
H	4.344070	1.009389	-2.408628
H	5.851913	0.243589	-3.000283
H	5.793612	0.953731	-1.359224
H	3.043646	-1.104104	-2.893449
H	6.674021	-1.266780	-0.539913
H	5.655215	-2.733895	-0.666174
H	3.609031	1.652849	2.767944
H	5.285432	1.897541	0.944821
H	7.560132	2.388755	1.834396
H	8.920814	0.565569	2.872411
H	7.983516	-1.750200	3.022574
H	5.712636	-2.234024	2.113619
H	1.236278	0.753715	-4.973915
H	2.294336	0.638689	-3.525105
H	1.041369	1.911858	-3.601968
H	-6.184660	2.328918	4.204383
H	-8.295217	0.990765	4.091278
H	-8.459756	-0.937331	2.507795
H	4.556003	-1.779206	-3.561489
H	6.495871	-2.094557	-2.107418
H	3.973199	2.258562	-0.673019

H	3.171503	3.707875	-0.052774
H	-1.644436	4.342240	-2.110682
H	-0.123859	4.109181	-3.003111
H	-2.533939	1.660288	1.102513
H	-1.436647	0.342661	1.504036
H	-2.490933	1.222120	3.587573
H	-0.736090	1.529703	3.604947
H	-1.861709	2.864179	3.228761
H	4.018899	4.077959	-2.350679
H	3.080018	2.679759	-2.960854
H	2.230303	4.127593	-2.374047
H	-1.988686	4.149756	-4.568885
H	-1.187718	2.551418	-4.592575
H	-2.762016	2.750008	-3.767836
H	1.165248	6.200627	-1.317891
H	0.638510	6.982031	0.217158
H	2.329418	6.504514	0.023533
H	0.831888	3.725449	2.175987
H	2.130783	4.998110	2.152824
H	0.432812	5.467960	2.337795

5.30 Transition State 32oa‡

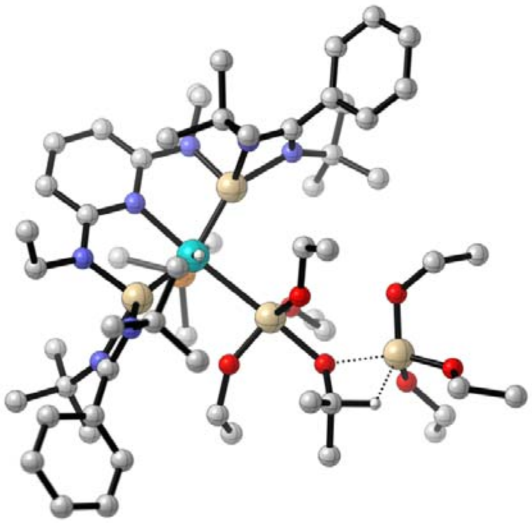

C	-0.537049	-4.180558	1.158485
N	-1.356764	-3.147759	0.747720

C	-2.677758	-3.436257	0.495763
C	-3.227152	-4.717552	0.753310
C	-2.399125	-5.719379	1.258169
C	-1.039053	-5.468264	1.457711
Fe	-0.677397	-1.114019	0.758602
Si	0.300400	1.399074	0.557193
O	1.040333	1.406319	-1.023597
C	1.670080	0.372147	-1.734806
C	1.936528	0.864991	-3.162679
N	-3.428961	-2.401182	-0.037477
C	-4.722594	-2.704181	-0.654217
C	-4.579690	-3.503849	-1.963219
N	0.806483	-3.867774	1.201550
C	1.778048	-4.943595	1.401389
C	1.994621	-5.275047	2.890775
Si	1.138778	-2.224799	0.445070
N	3.072898	-2.282405	0.478885
C	4.161622	-1.798180	1.355163
C	5.207725	-2.910495	1.600447
N	1.885831	-2.907434	-1.221121
C	1.372232	-3.594550	-2.424692
C	2.080457	-4.953423	-2.635067
C	3.134614	-2.681032	-0.808972
C	4.366755	-2.810695	-1.641732
C	4.733280	-1.765786	-2.512407
C	5.881734	-1.879445	-3.309682
C	6.669564	-3.040729	-3.249770
C	6.305546	-4.086845	-2.385277
C	5.160735	-3.971895	-1.583315
C	-0.128551	-3.862764	-2.172709
C	1.528193	-2.715917	-3.686419
C	3.511265	-1.416920	2.692150
C	4.840257	-0.543326	0.780644
P	-0.901452	-1.083498	2.958678
C	-2.117168	-2.366994	3.621389
Si	-2.663806	-0.749566	0.042694
N	-4.340670	0.245128	0.377588

C	-5.377316	0.290557	1.433310
C	-5.036822	-0.843902	2.420459
C	-1.521256	0.452126	3.836162
C	0.453708	-1.542534	4.174677
N	-3.403929	0.026173	-1.569397
C	-4.404604	0.633283	-0.903472
C	-5.410723	1.580184	-1.469018
C	-6.514507	1.089605	-2.195209
C	-7.490706	1.967452	-2.685097
C	-7.373815	3.349362	-2.459120
C	-6.274247	3.845681	-1.740538
C	-5.298493	2.966881	-1.246166
C	-2.954274	0.308053	-2.957129
C	-2.483761	1.773214	-3.056938
C	-4.074840	0.014186	-3.983048
C	-1.759223	-0.609812	-3.281522
C	-6.803780	0.037420	0.887104
C	-5.356319	1.642116	2.185668
O	-1.320436	1.978839	0.542814
C	-2.213856	2.993153	0.952393
C	-1.956292	3.534659	2.365916
O	1.158784	1.293355	2.057837
C	1.805889	2.256316	2.897828
C	1.929414	1.741177	4.335958
O	0.944966	3.231558	0.446883
C	0.452074	4.284455	-0.292369
C	-0.171234	5.453108	0.530670
C	-0.214397	4.060413	-1.649382
O	2.854404	4.674488	1.570599
C	3.361877	5.982254	1.885434
O	3.508437	2.537173	-0.183525
C	4.659415	2.345700	-1.034372
O	3.689067	5.078500	-0.873629
C	3.340053	5.663846	-2.132282
H	5.915504	-2.575617	2.376558
H	4.280731	-1.020951	3.374291
H	-0.738845	-0.887564	-0.746234

H	-4.278790	-4.917060	0.562971
H	-2.810246	-6.707320	1.480179
H	-0.377182	-6.251125	1.819317
H	-5.213727	-1.748742	-0.873106
H	-5.385722	-3.234324	0.055329
H	-3.995225	-2.914635	-2.686640
H	-5.569077	-3.724935	-2.400127
H	-4.052461	-4.452260	-1.783000
H	2.732656	-4.614814	0.968580
H	1.468580	-5.851706	0.849146
H	2.395598	-4.392943	3.413885
H	2.708115	-6.108964	3.009822
H	1.045223	-5.552487	3.373461
H	-5.072946	-1.819109	1.914316
H	-0.906669	-0.375844	-2.635990
H	-1.468664	-0.453660	-4.332907
H	-2.023834	-1.668123	-3.142196
H	-2.002429	1.956301	-4.032566
H	-1.758885	1.951915	-2.254727
H	-4.515399	-0.977779	-3.796581
H	-3.642118	0.017977	-4.996801
H	-4.869067	0.770779	-3.950253
H	0.953427	-1.786417	-3.578636
H	1.145870	-3.263871	-4.563709
H	2.583817	-2.466392	-3.866246
H	3.125789	-4.826770	-2.949063
H	1.550566	-5.518969	-3.418907
H	2.051509	-5.539229	-1.702825
H	-0.259706	-4.626277	-1.392932
H	-0.592874	-4.226828	-3.103279
H	-0.637790	-2.949038	-1.839529
H	5.310451	-0.749364	-0.190939
H	5.626282	-0.204949	1.475924
H	4.097770	0.257350	0.673714
H	4.711587	-3.828040	1.954642
H	5.780607	-3.139541	0.691792
H	2.744466	-0.649302	2.526217

H	3.047377	-2.294657	3.161890
H	4.115964	-0.868183	-2.554503
H	4.872264	-4.781812	-0.912182
H	1.253124	-0.796927	4.165747
H	0.861261	-2.515665	3.864581
H	0.048626	-1.628938	5.198617
H	-1.623535	-3.349903	3.594384
H	-3.017787	-2.432734	3.000488
H	-2.401292	-2.140596	4.663804
H	-4.385545	1.793992	2.679261
H	-6.145510	1.649164	2.956070
H	-5.540284	2.476370	1.493113
H	-4.029372	-0.711451	2.828357
H	-7.185124	0.890016	0.310976
H	-6.821290	-0.861016	0.251723
H	-3.327961	2.471125	-2.948688
H	-4.439945	3.351882	-0.698144
H	-6.174044	4.918868	-1.563813
H	-8.134324	4.033686	-2.840849
H	-8.342523	1.572600	-3.242873
H	-6.604741	0.016289	-2.365584
H	-1.574990	0.308591	4.929990
H	-2.515273	0.727597	3.458124
H	-0.827758	1.268383	3.599050
H	6.158160	-1.062208	-3.979597
H	7.561795	-3.130811	-3.872850
H	6.912526	-4.993227	-2.335107
H	-5.758632	-0.839497	3.252248
H	-7.481506	-0.128301	1.740235
H	-3.210388	2.530158	0.925834
H	-2.243317	3.817896	0.219102
H	2.820260	2.457271	2.518330
H	1.262049	3.214444	2.891228
H	2.626885	0.104541	-1.244992
H	1.050772	-0.535149	-1.743330
H	2.456557	0.096749	-3.758525
H	0.982662	1.102789	-3.662055

H	2.553349	1.779698	-3.142414
H	-2.549845	4.447825	2.547185
H	-2.233561	2.784102	3.116486
H	-0.892652	3.772132	2.503261
H	2.408860	2.521057	4.954246
H	0.948913	1.502846	4.773095
H	2.556218	0.837762	4.378788
H	0.208938	5.411047	1.557781
H	0.133969	6.413674	0.080895
H	-1.267846	5.434986	0.543494
H	0.157446	3.140673	-2.110240
H	-1.303083	3.989803	-1.534172
H	-0.003060	4.932357	-2.291682
H	1.435889	4.874563	-0.549750
C	3.168334	6.206126	3.385503
H	4.426626	6.052181	1.607115
H	2.814939	6.745437	1.303561
C	4.464325	6.610117	-2.555114
H	3.193884	4.874497	-2.894630
H	2.385387	6.218293	-2.042174
H	4.628195	1.291799	-1.338952
H	4.568732	2.972842	-1.935407
C	5.952278	2.682977	-0.292130
Si	2.781074	3.985425	0.069645
H	3.548620	7.200136	3.673428
H	2.100319	6.143715	3.646725
H	3.710071	5.436993	3.957473
H	4.221498	7.083070	-3.520540
H	4.602389	7.397032	-1.797248
H	5.409565	6.054755	-2.658441
H	6.826025	2.429811	-0.916381
H	5.979470	3.759838	-0.061940
H	6.011775	2.110567	0.644249

6 NMR Spectra

Iron(II) complex **7a**

^{1}H NMR (500 MHz, C_6D_6):

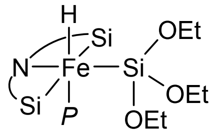

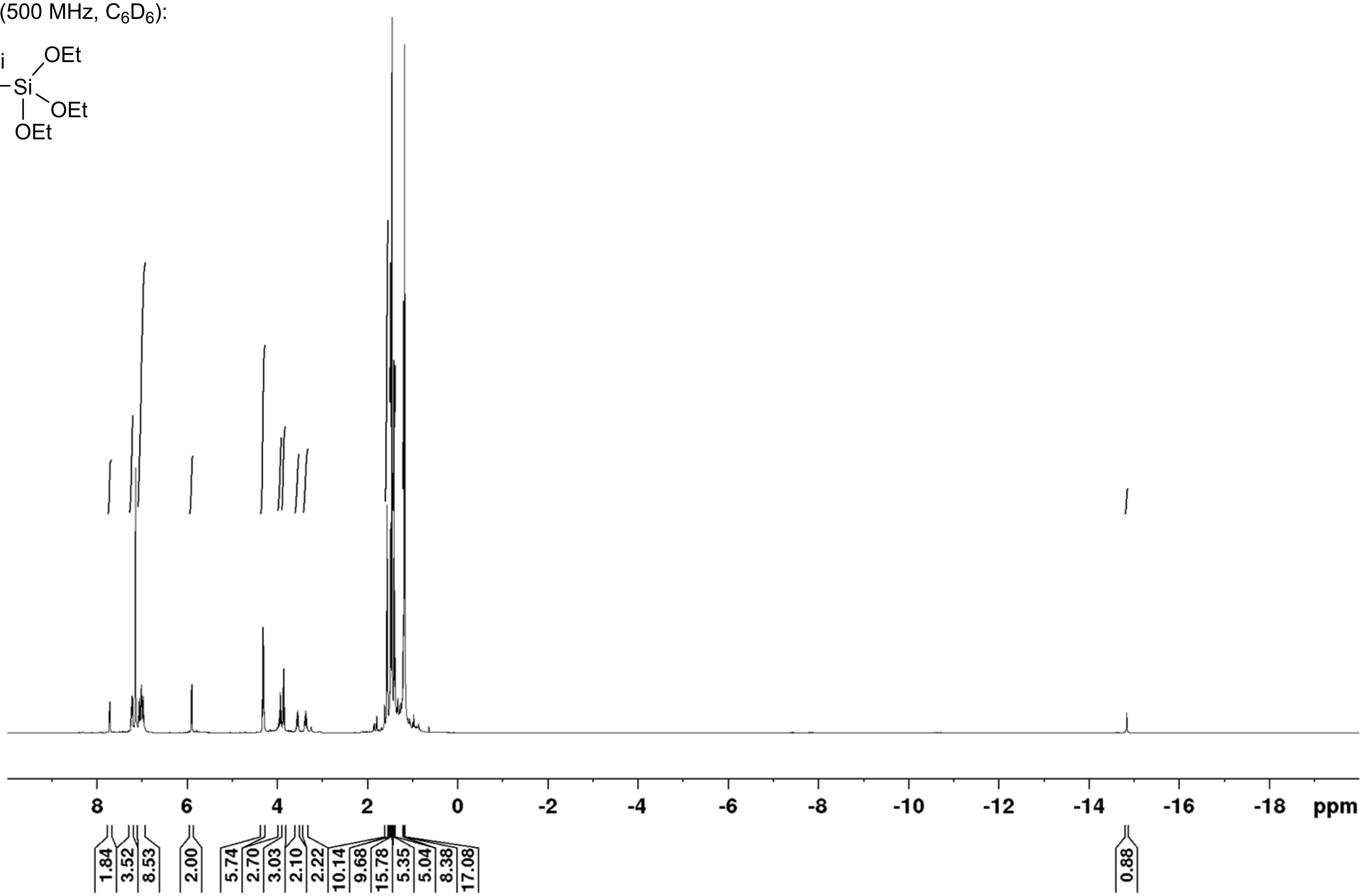

^{13}C NMR (126 MHz, C_6D_6):

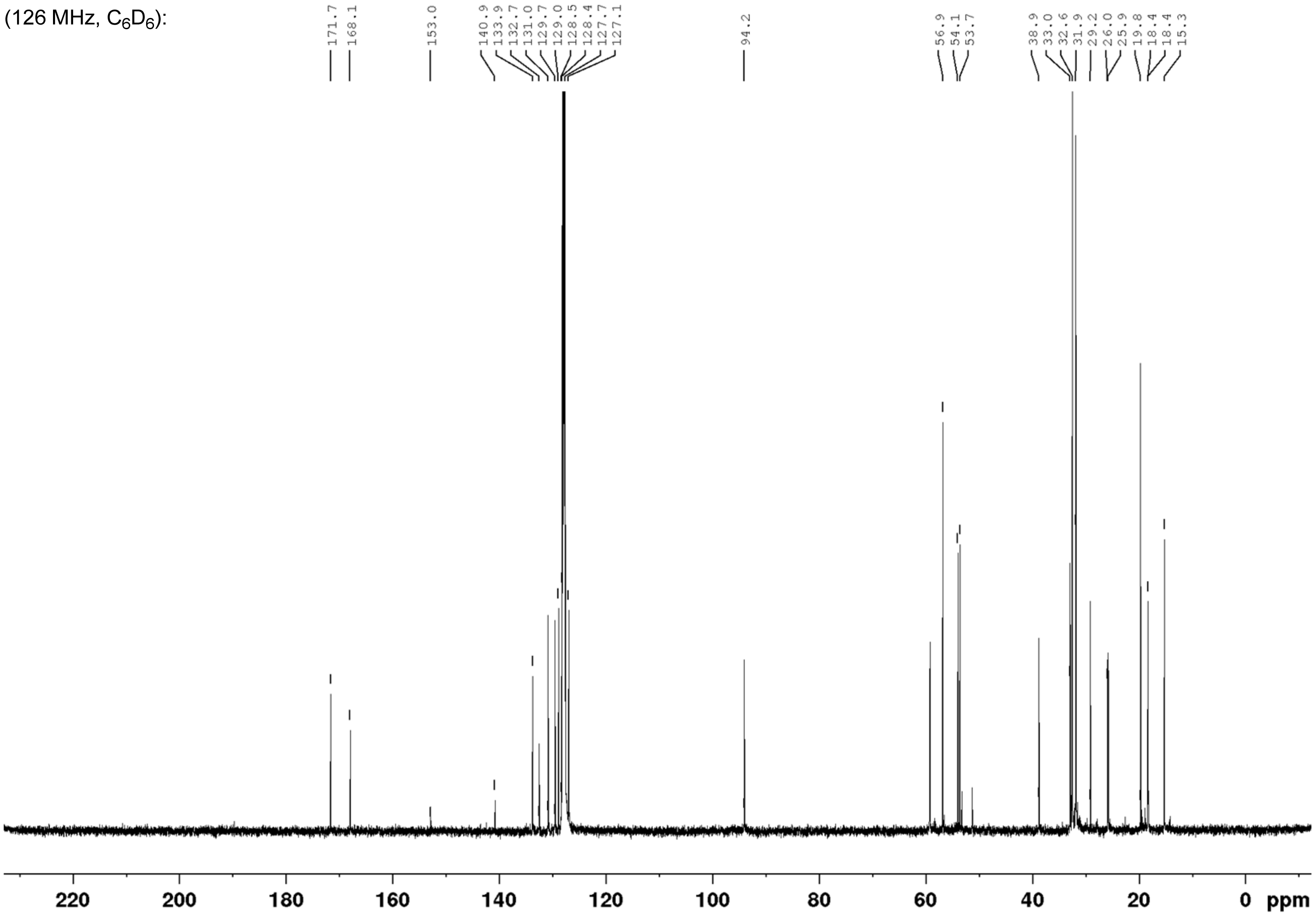

$^{31}P\{^{1}H\}$ NMR (202 MHz, C_6D_6):

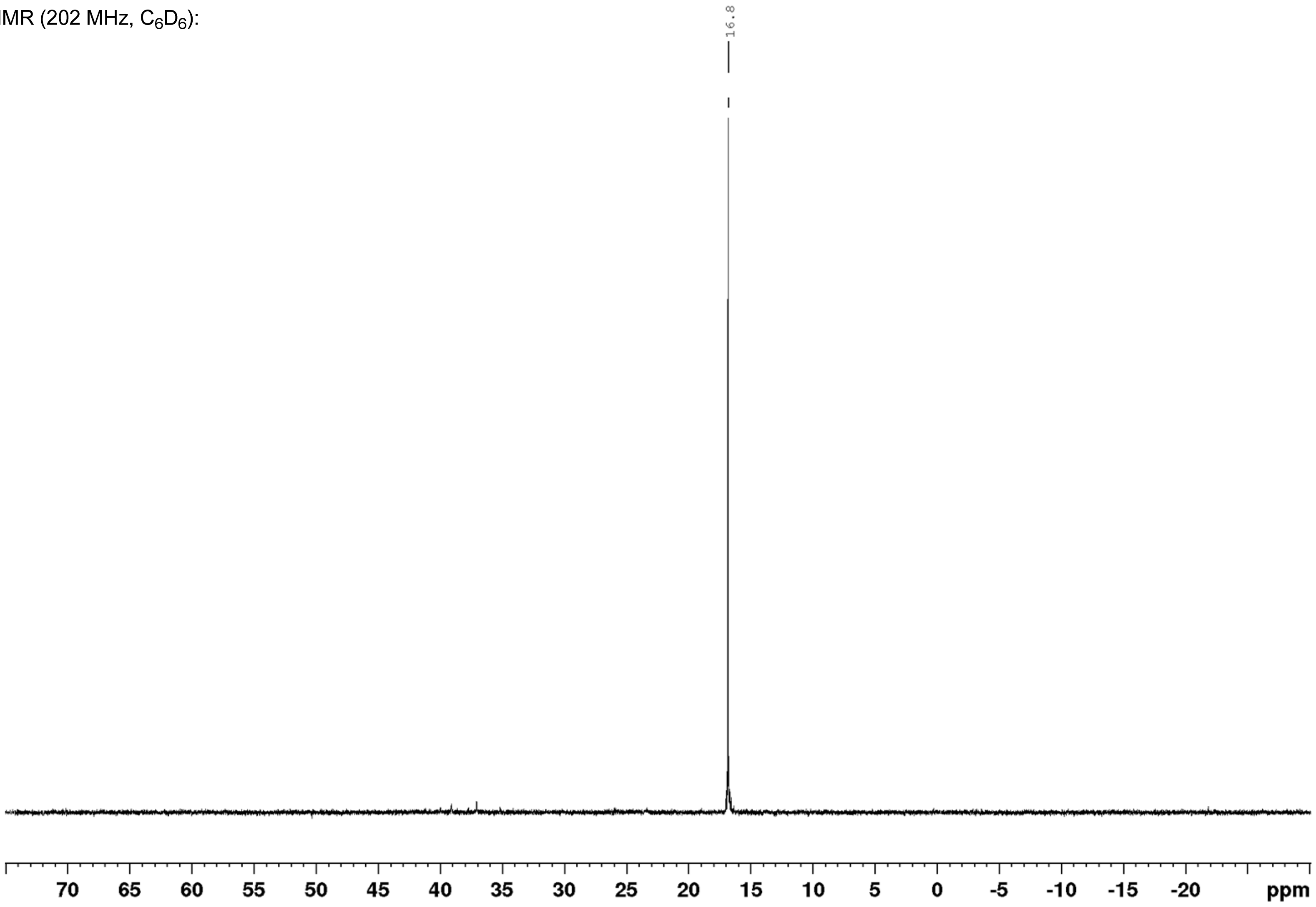

$^{1}H,^{29}Si$ HMQC (500 MHz/ 99 MHz, C_6D_6):

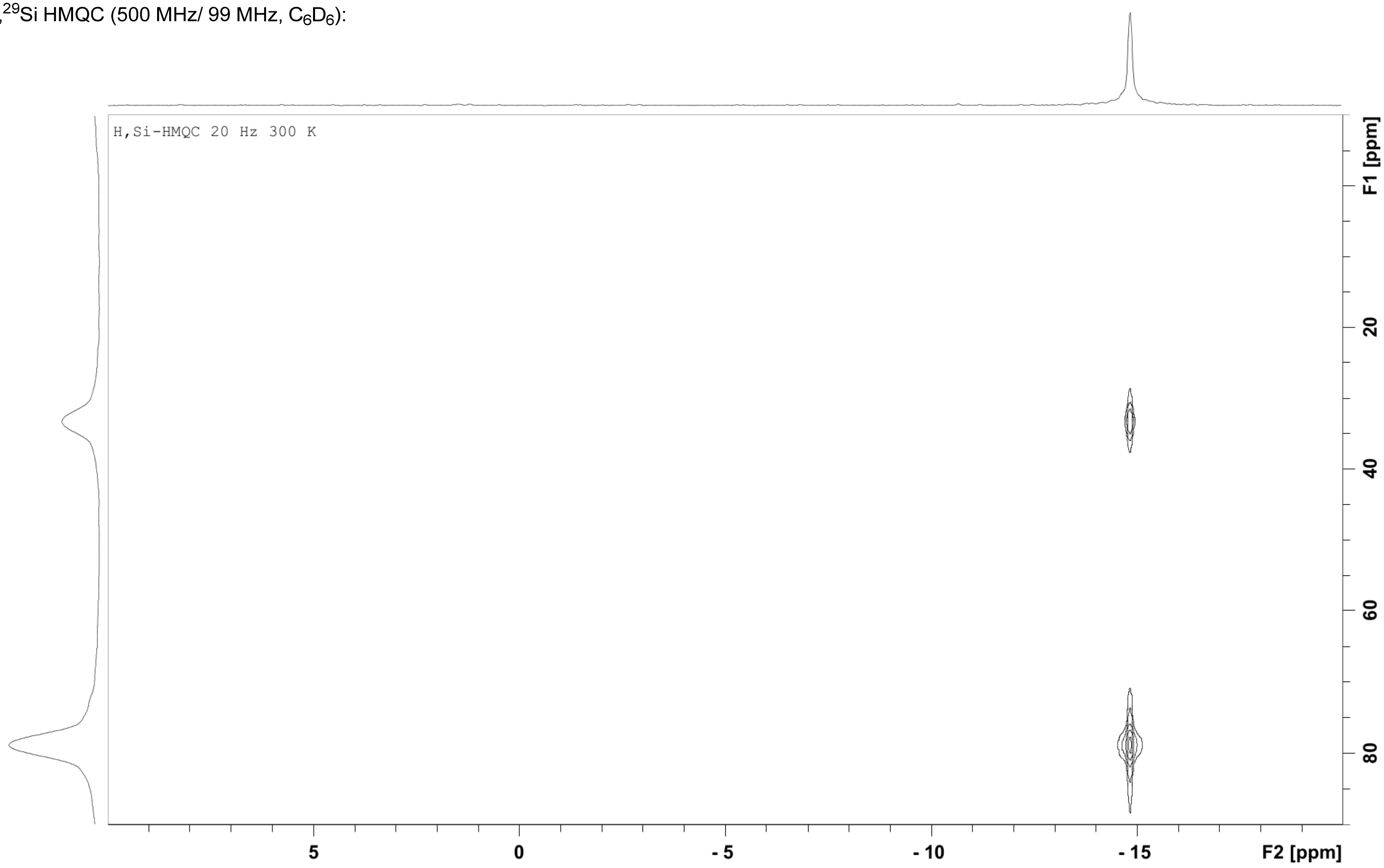

Iron(II) complex **7b**

^{1}H NMR (500 MHz, C_6D_6):

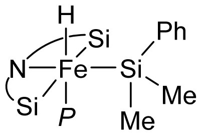

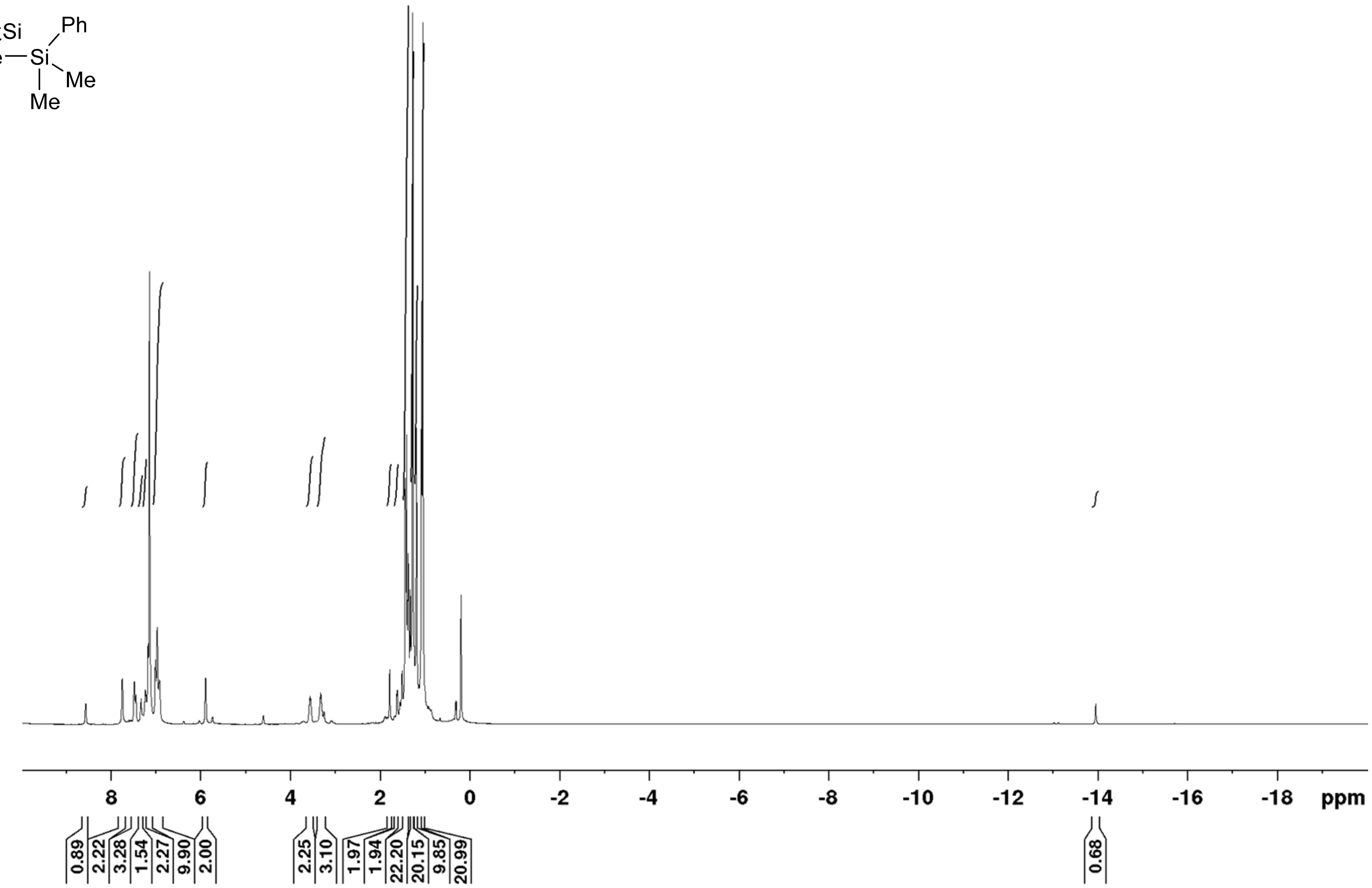

^{13}C NMR (126 MHz, C_6D_6):

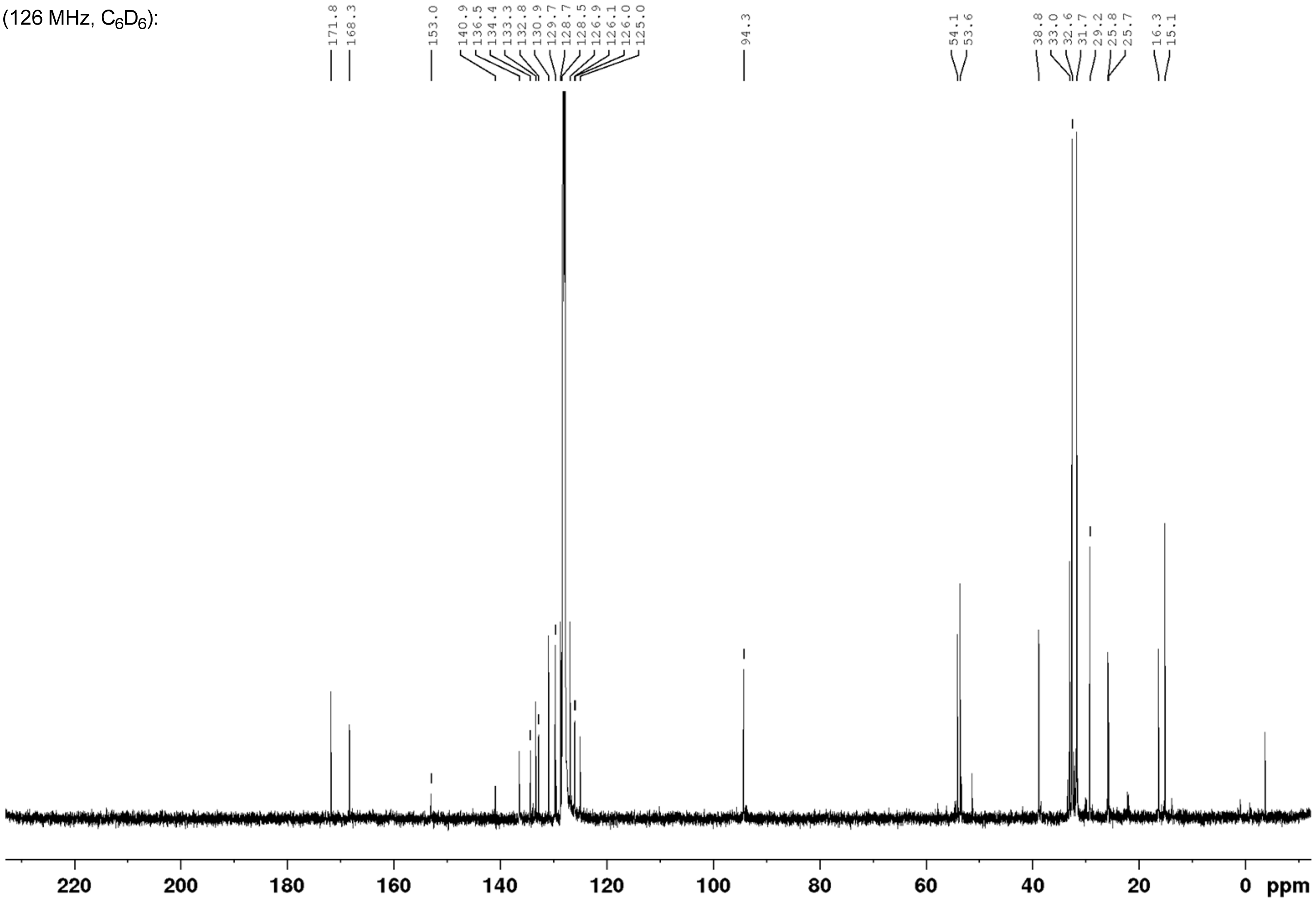

$^{31}P\{^{1}H\}$ NMR (202 MHz, C_6D_6):

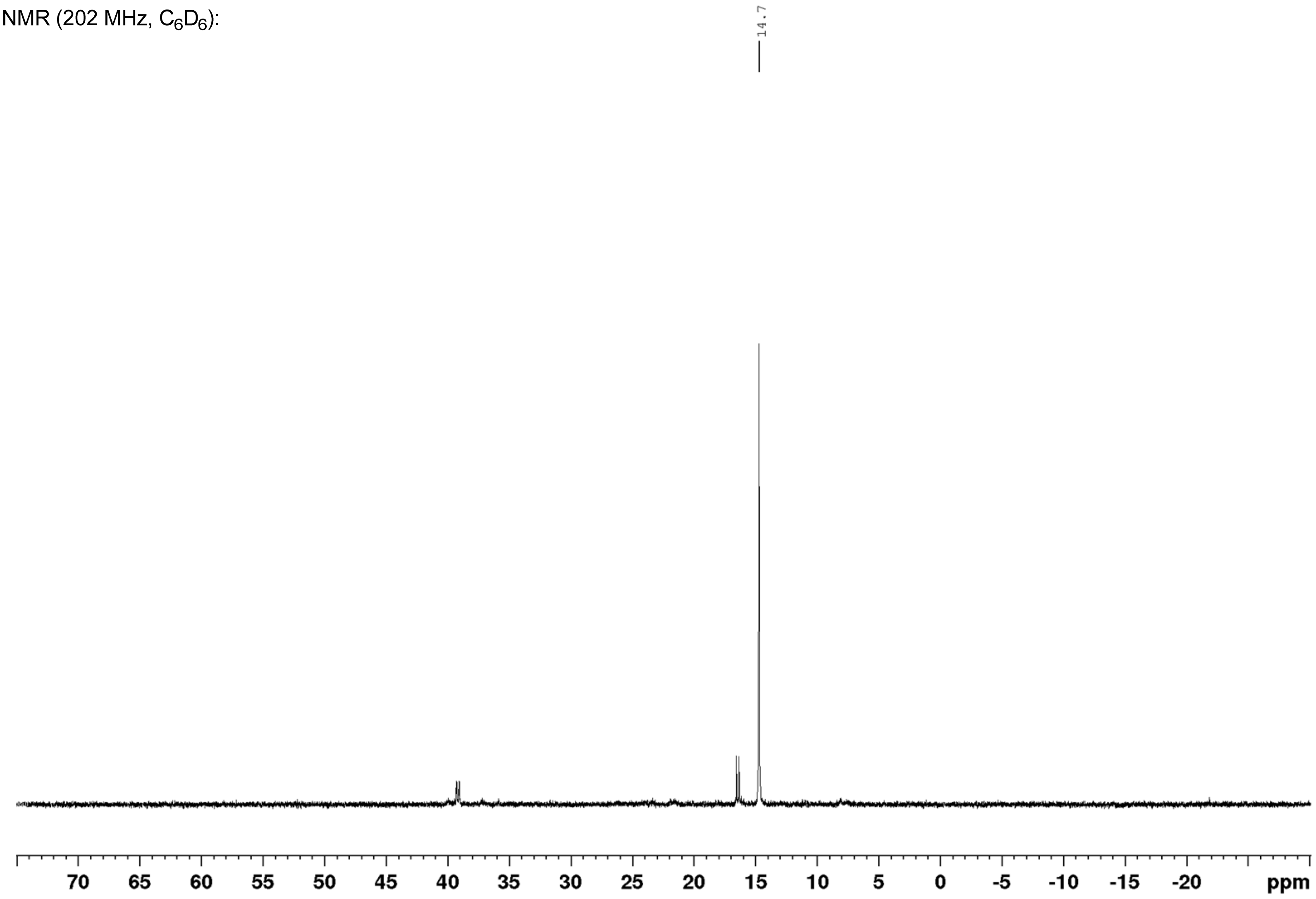

$^{1}H,^{29}Si$ HMQC (500 MHz/ 99 MHz, C_6D_6):

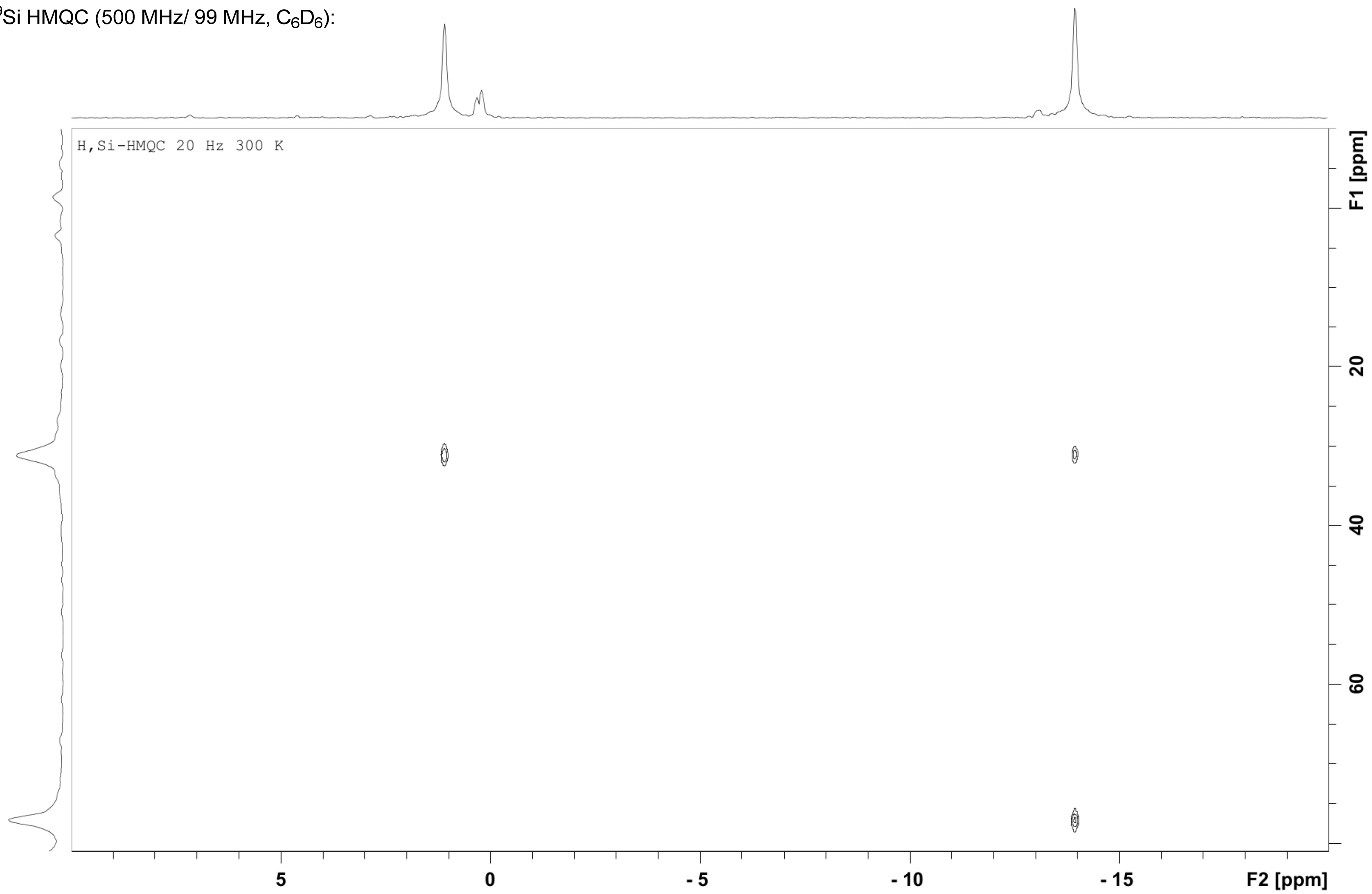

Iron(II) complex **7c**

^1H NMR (500 MHz, C_6D_6):

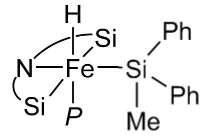

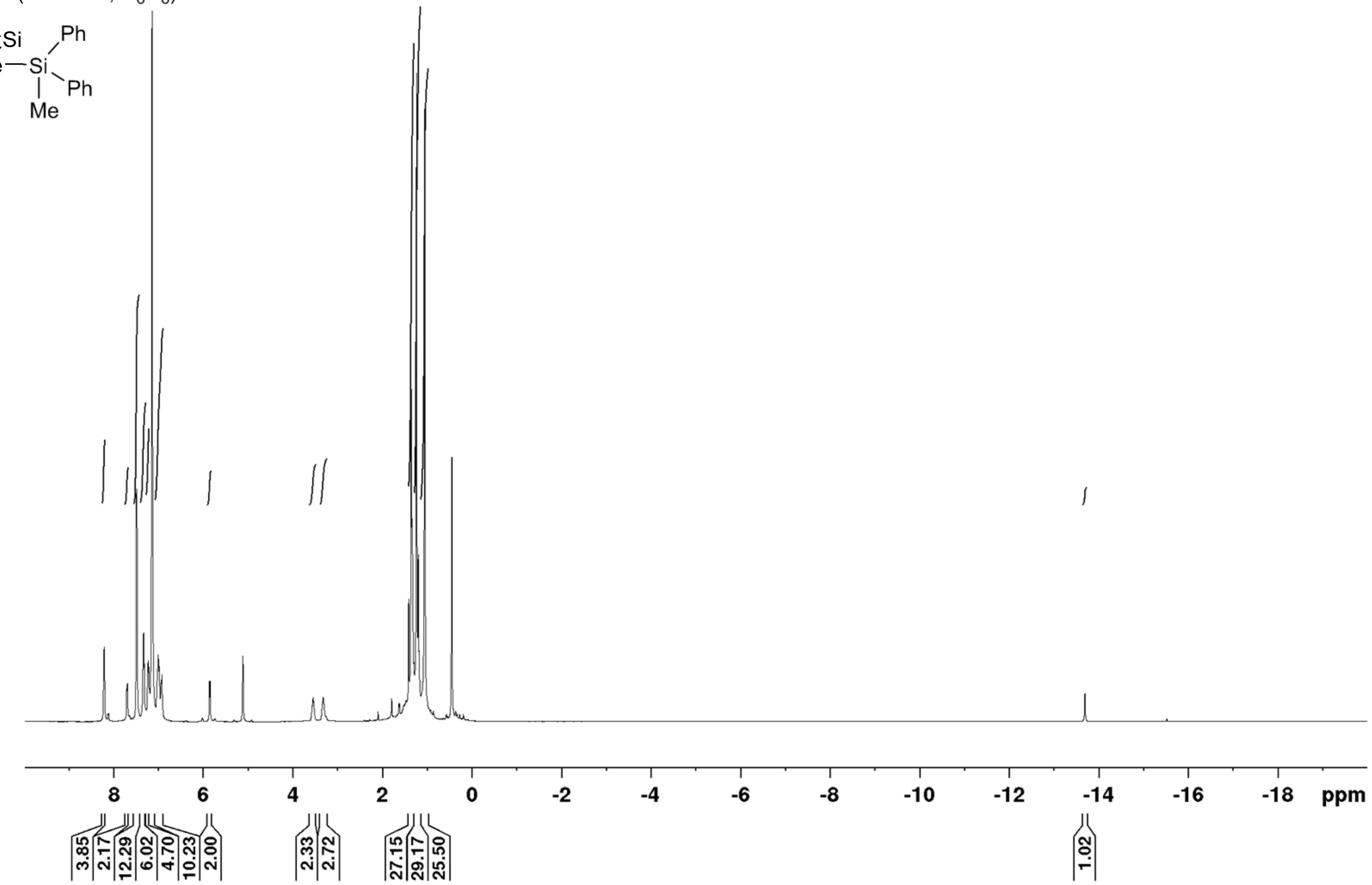

^{13}C NMR (126 MHz, C_6D_6):

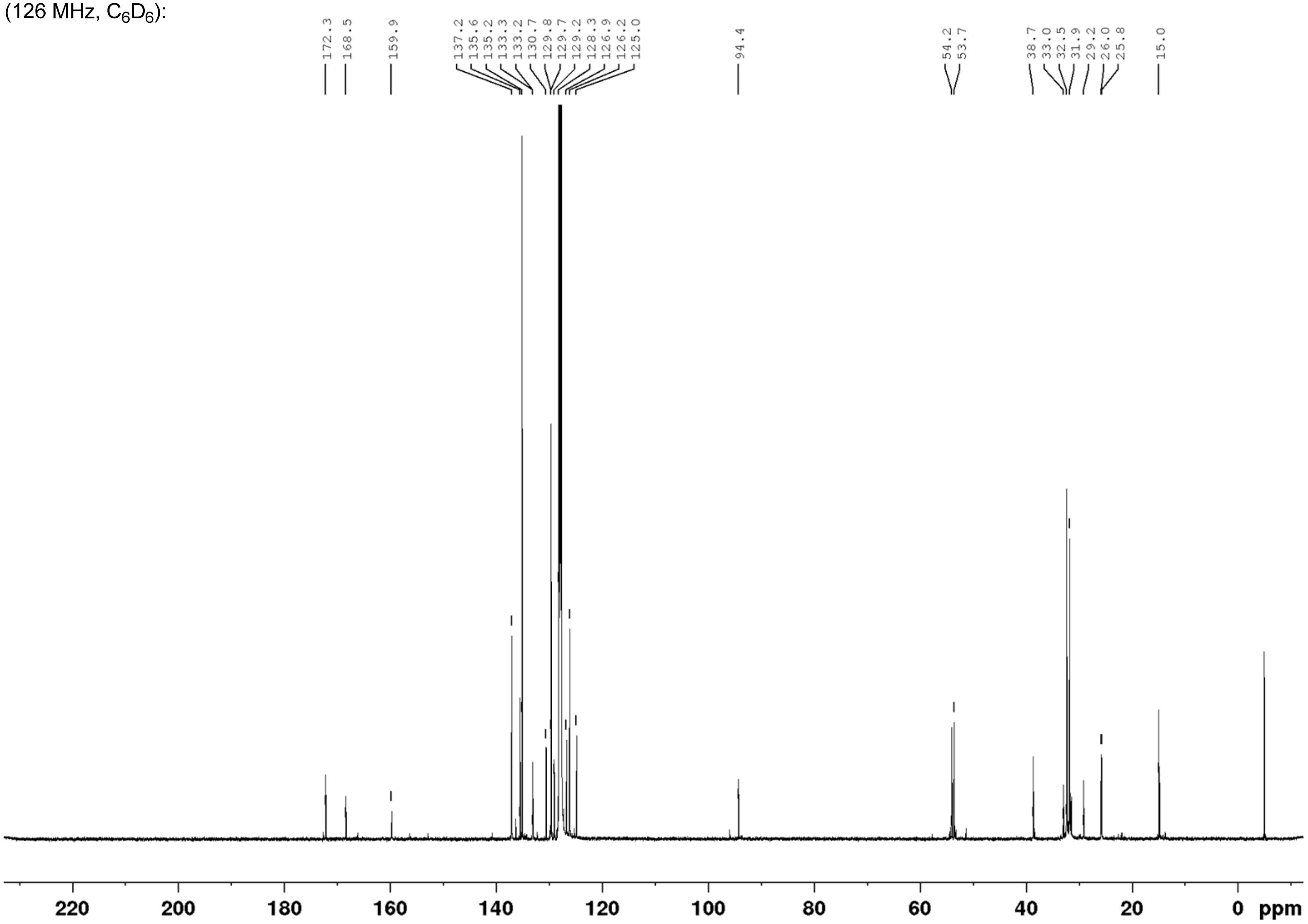

$^{31}P\{^{1}H\}$ NMR (202 MHz, C_6D_6):

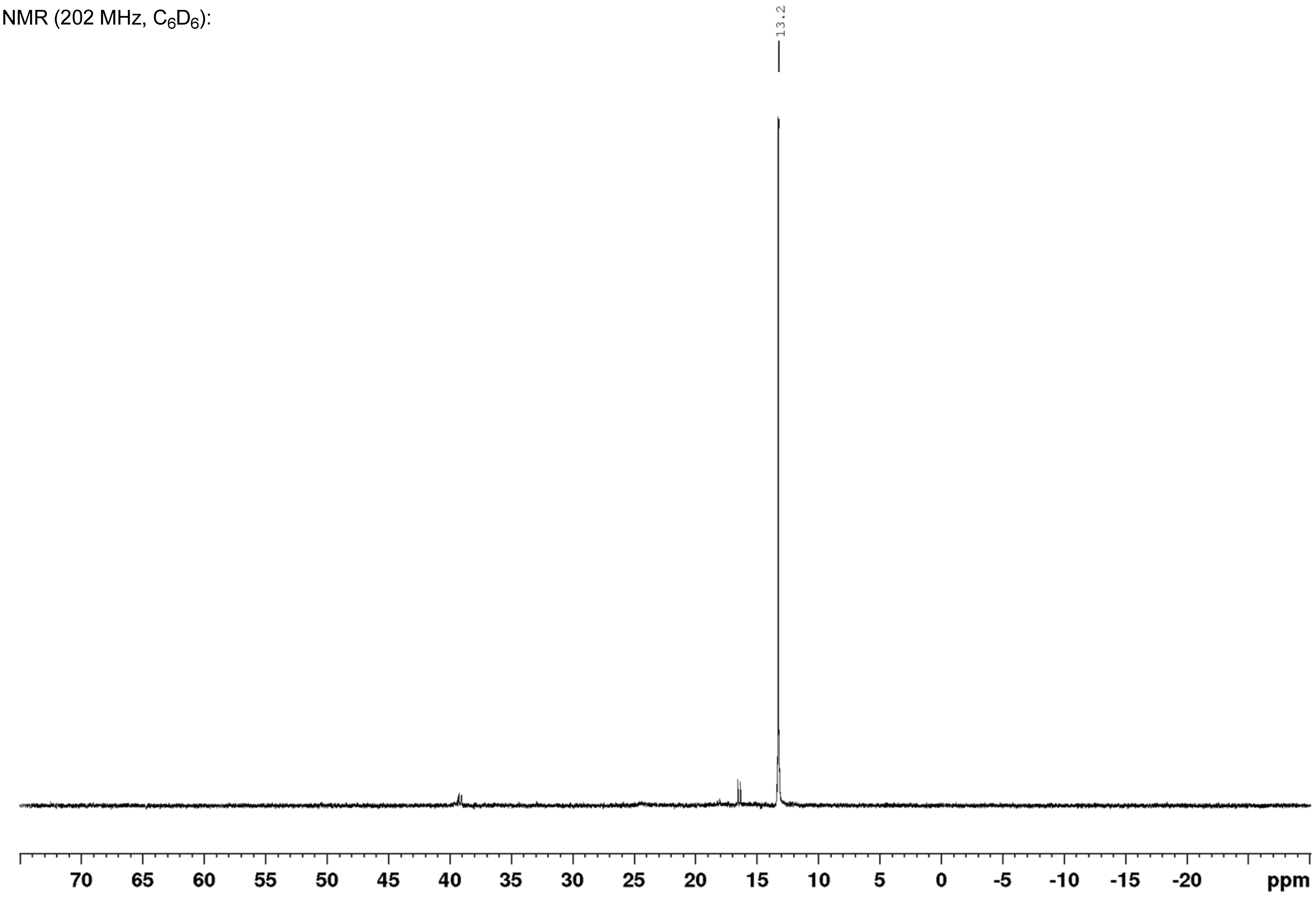

$^{1}H,^{29}Si$ HMQC (500 MHz/ 99 MHz, C_6D_6):

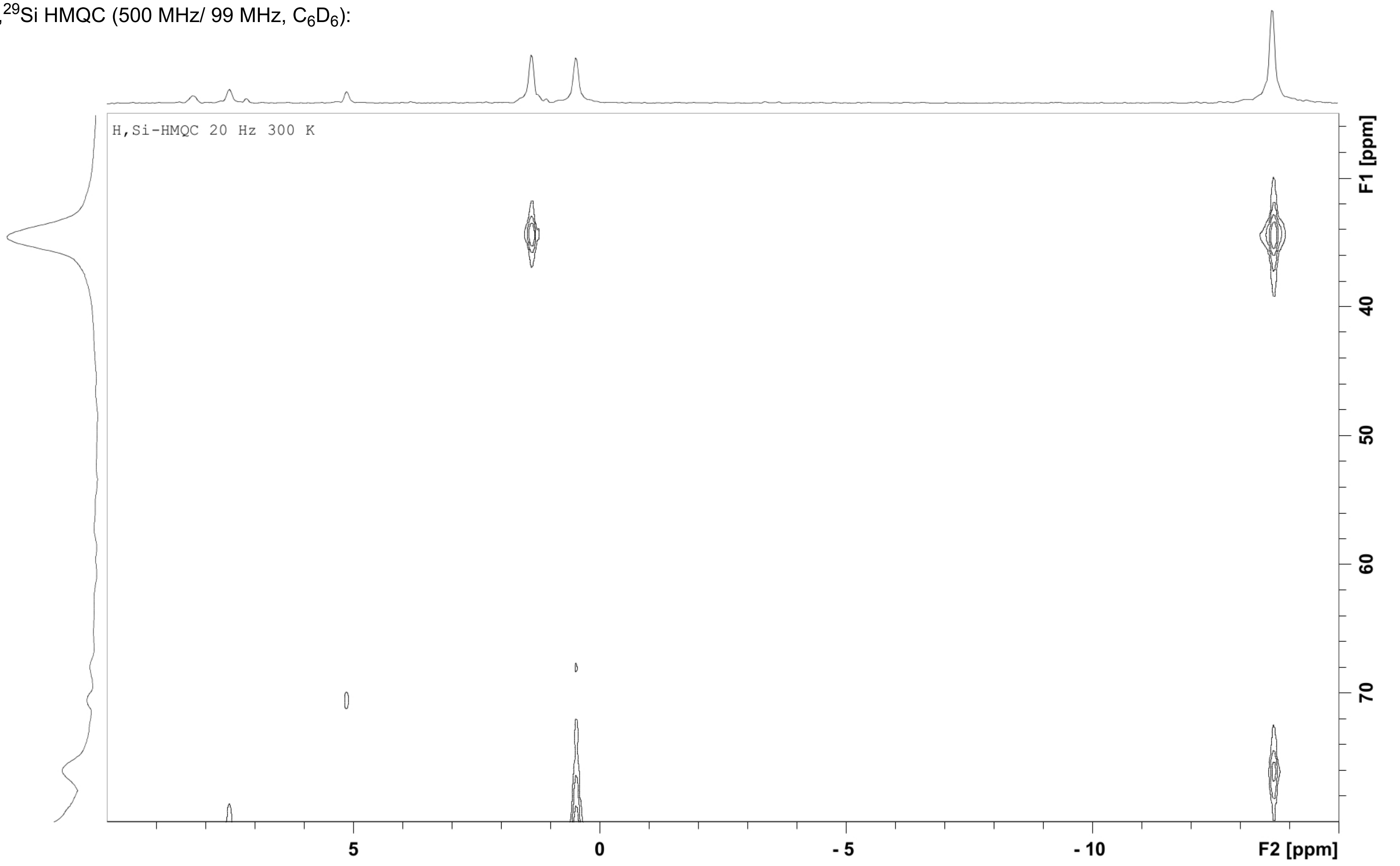

Trimethylphosphine-d_9 **6**-d_9
^1H NMR (500 MHz, C_6D_6/C_6H_6):

$P(CD_3)_3$

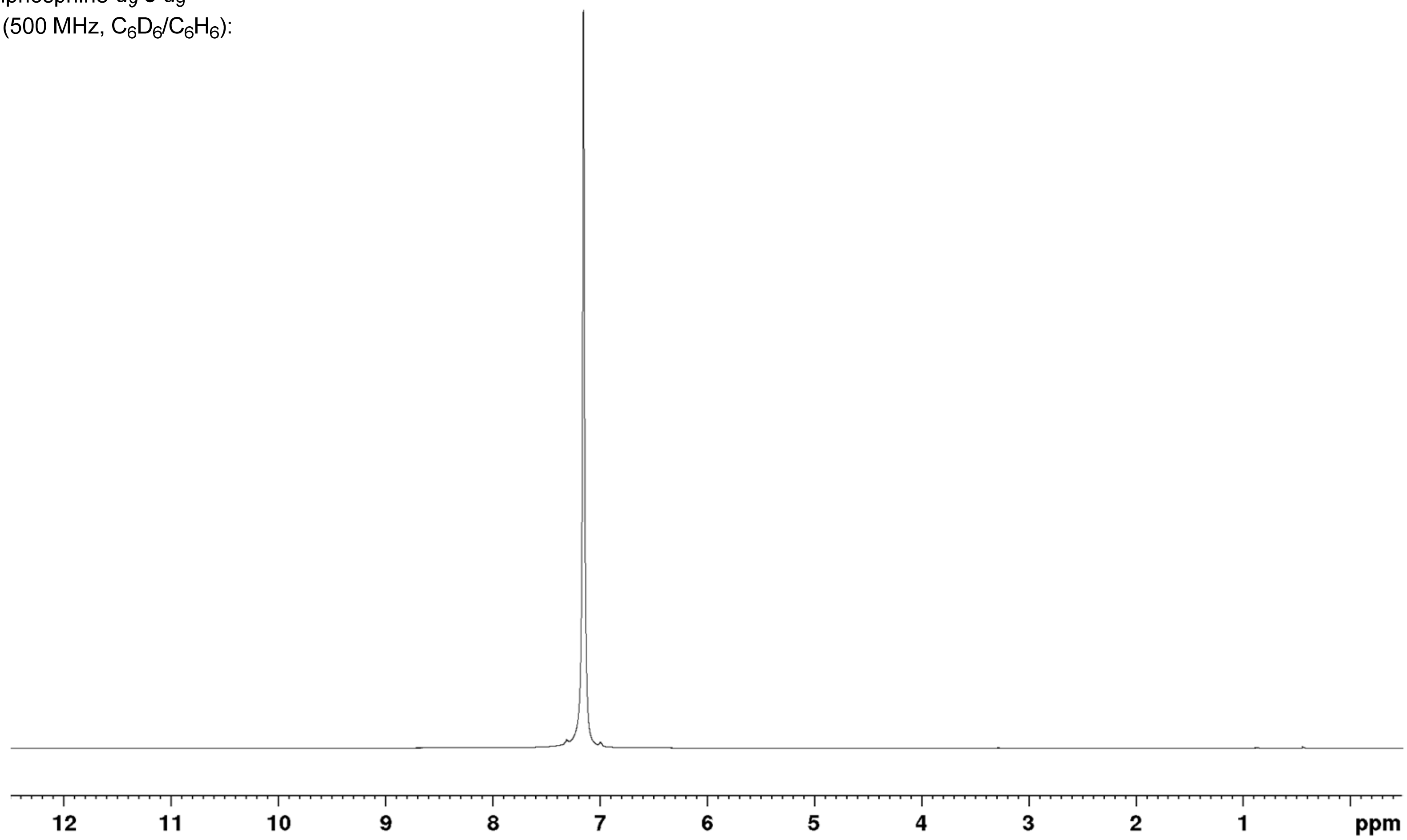

^2H NMR (77 MHz, C_6D_6/C_6H_6):

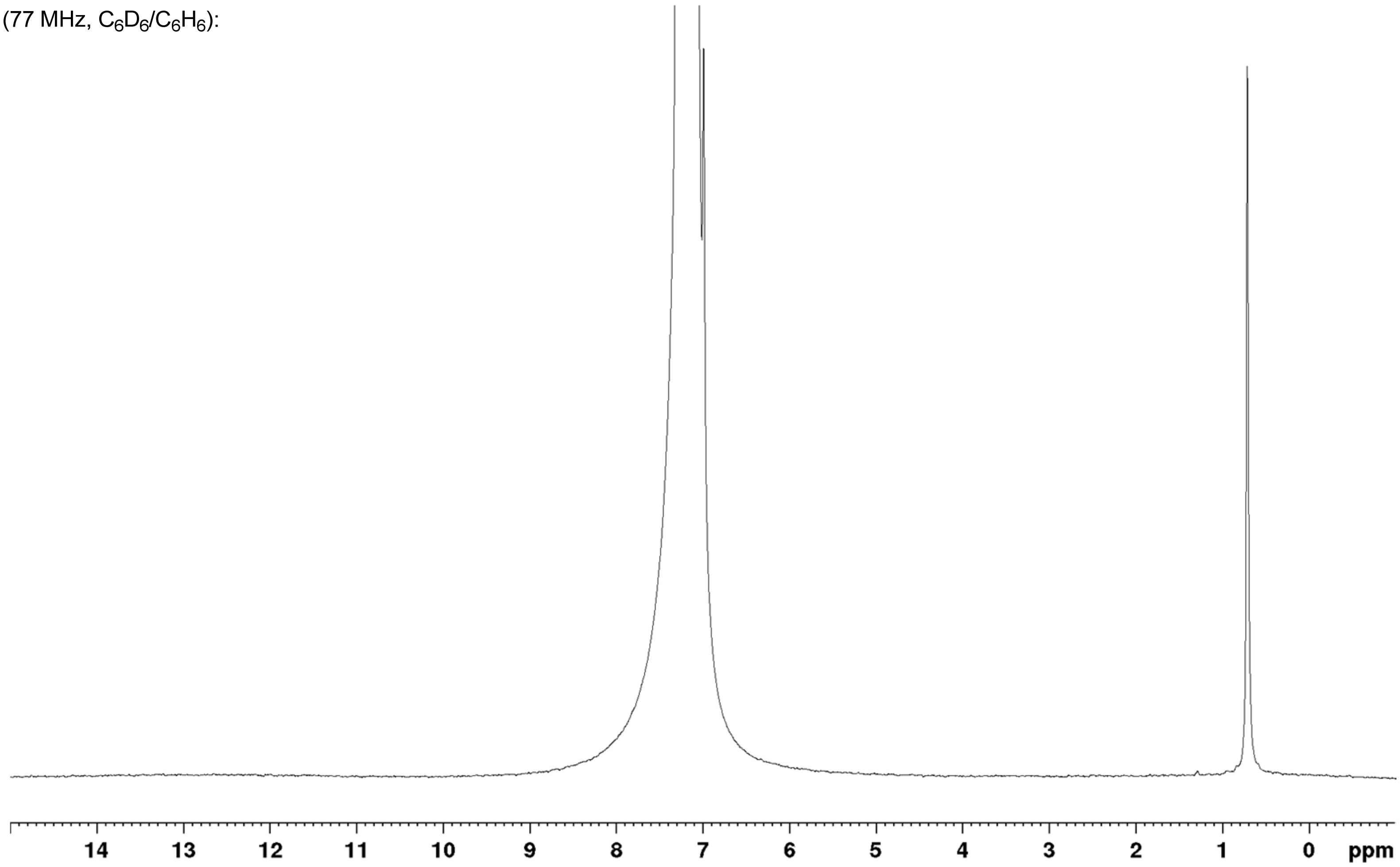

^{13}C NMR (176 MHz, C_6D_6/C_6H_6):

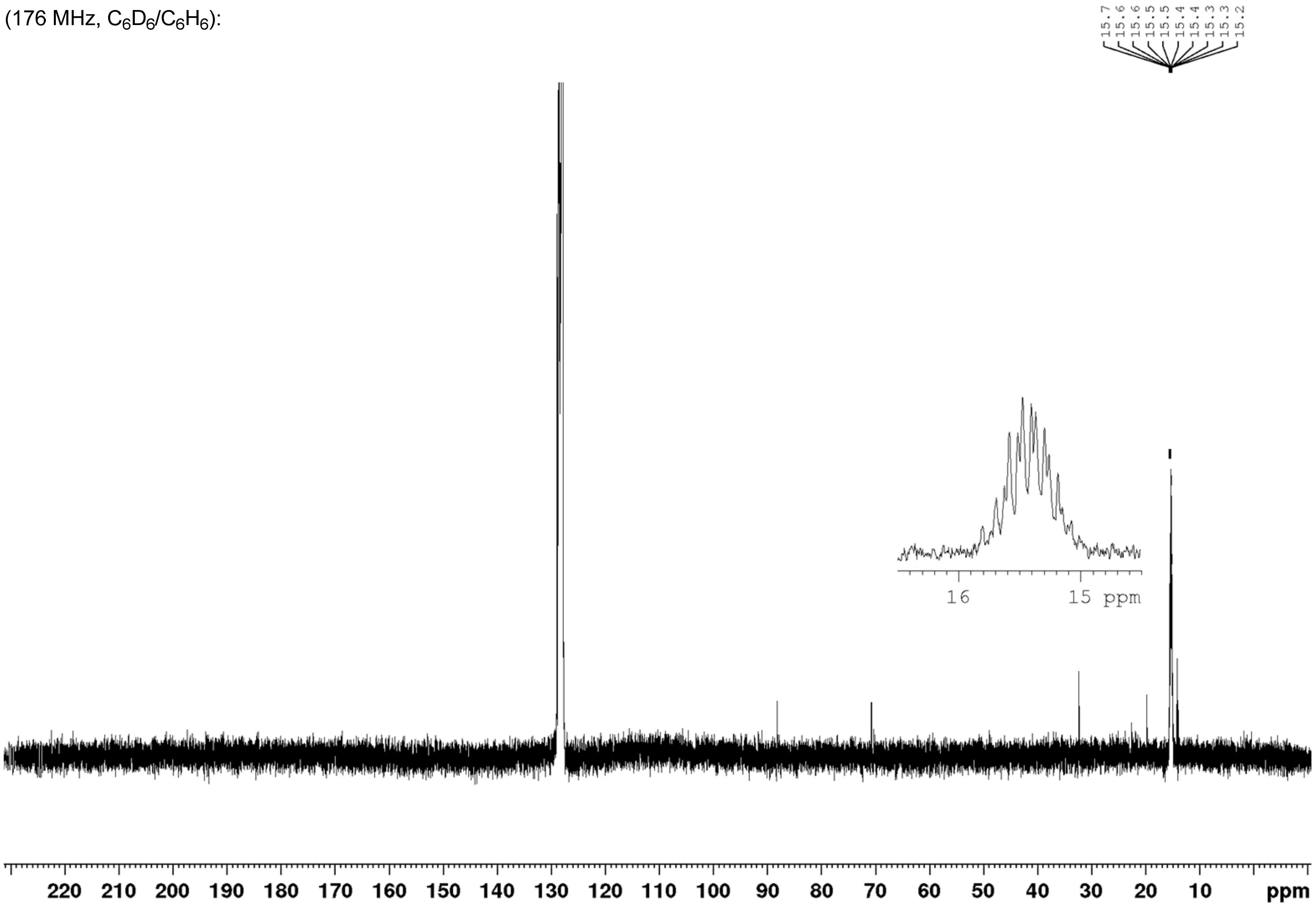

^{31}P NMR (202 MHz, C_6D_6/C_6H_6):

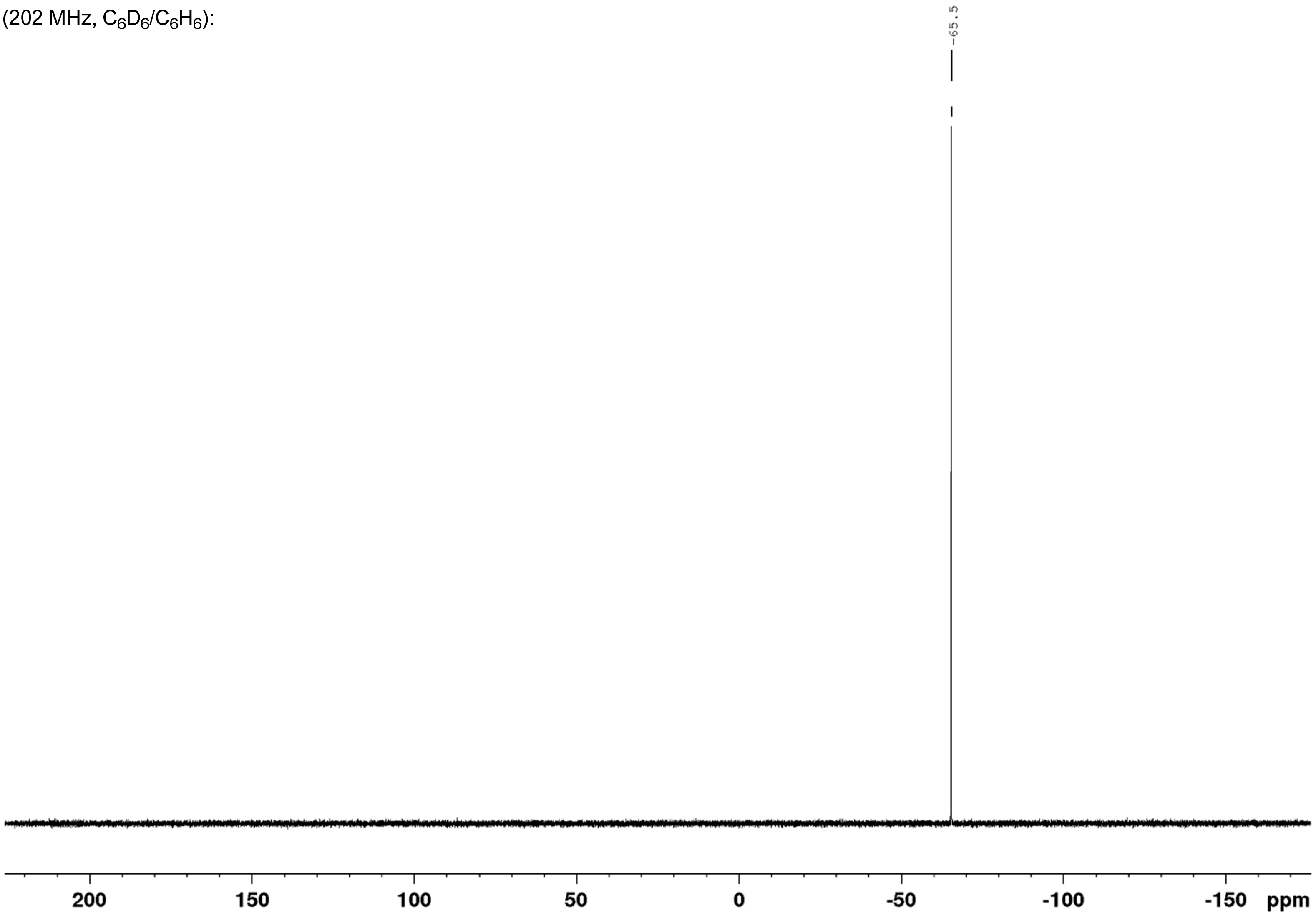

Dimethyl(phenyl)(1-phenylethoxy-1-*d*)silane (**8eb**-d_1)

^{1}H NMR (500 MHz, $CDCl_3$):

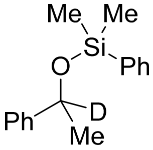

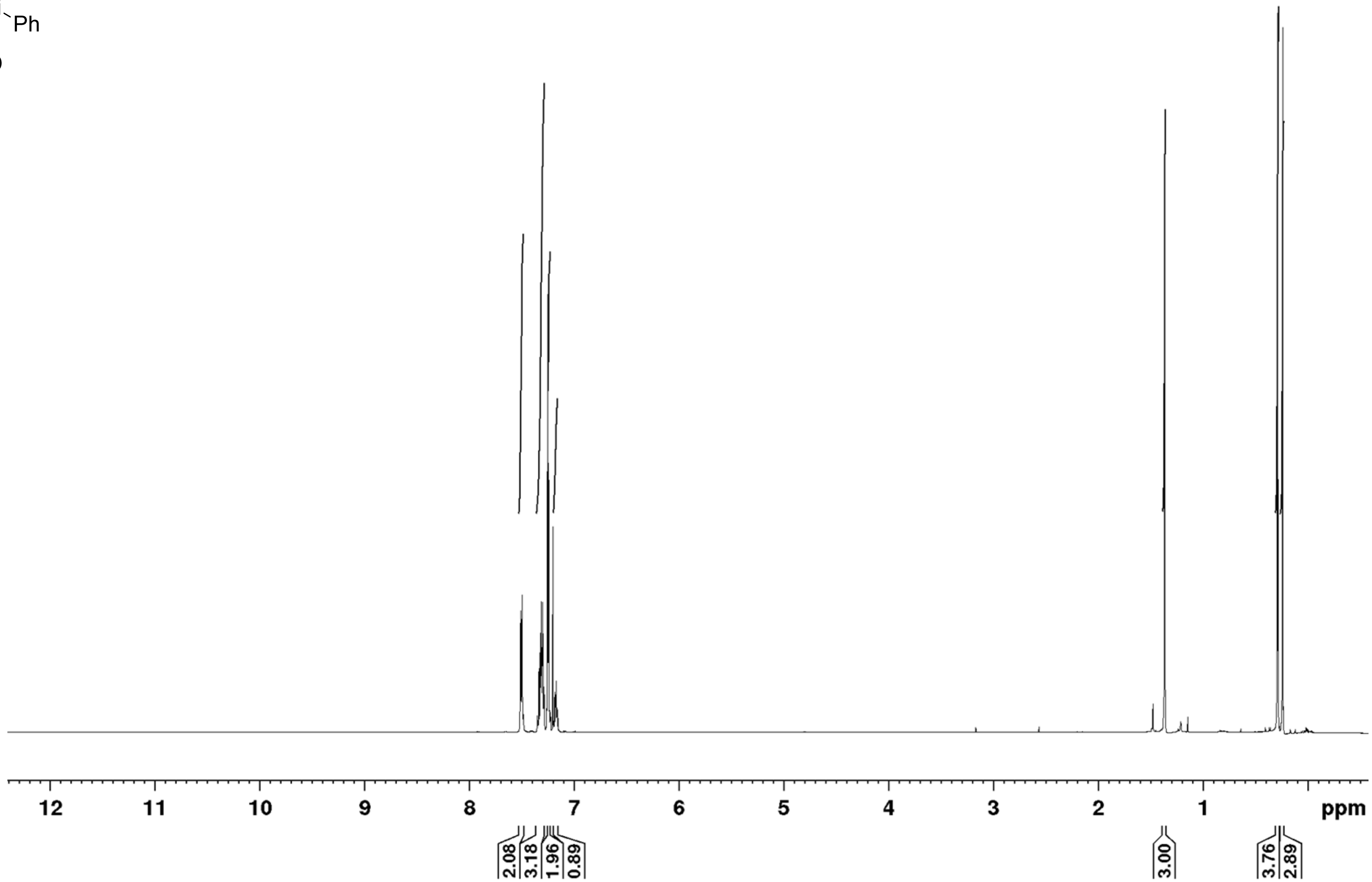

^{13}C NMR (126 MHz, C_6D_6):

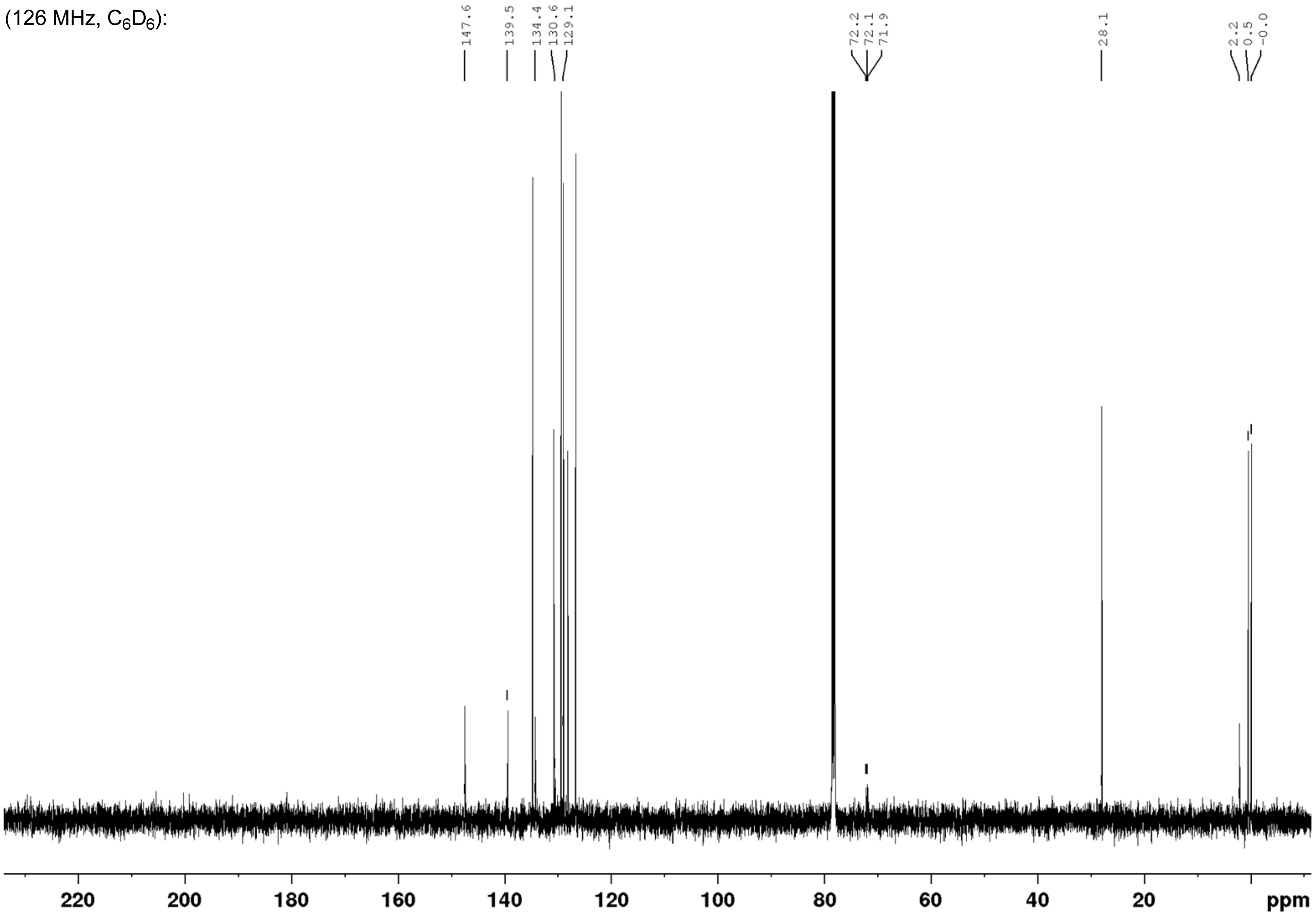

^{29}Si DEPT NMR (99 MHz, $CDCl_3$):

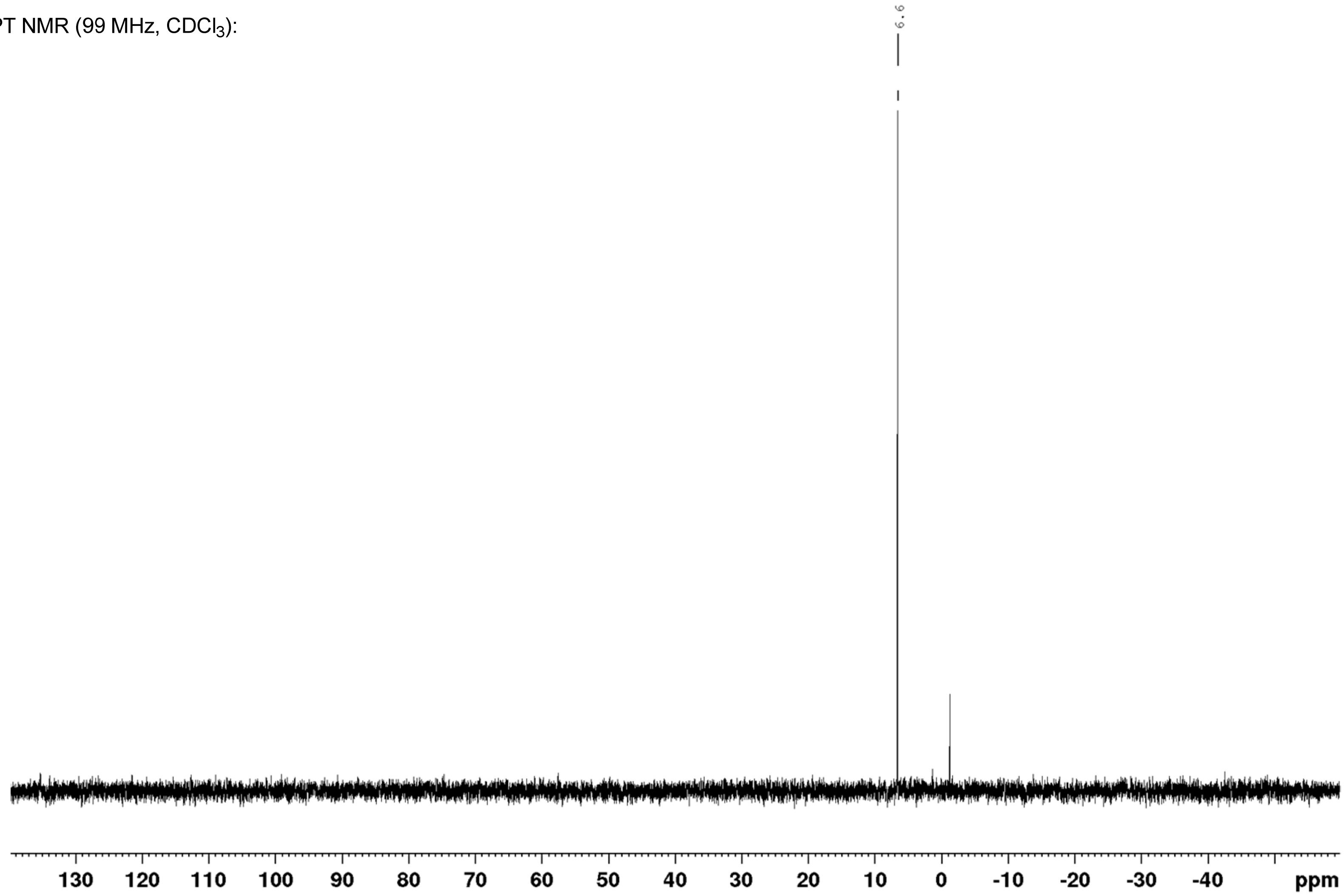

7 Gibbs Free Energy Profile

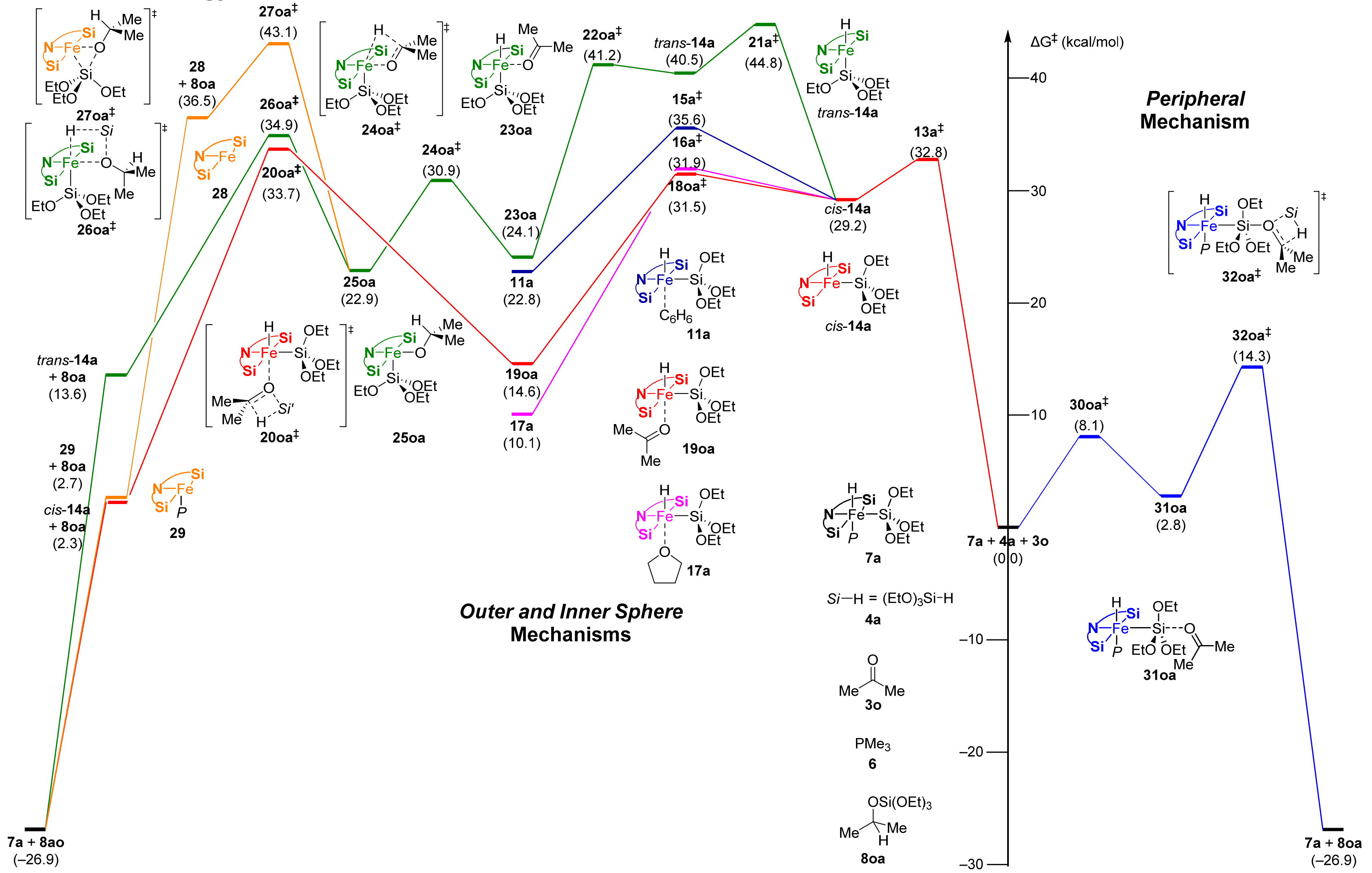

8 References

[S1] D. Gallego, S. Inoue, B. Blom and M. Driess, *Organometallics*, 2014, **33**, 6885–6897.

[S2] (*a*) H. F. T. Klare, M. Oestreich, J.-i. Ito, H. Nishiyama, Y. Ohki and K. Tatsumi, *J. Am. Chem. Soc.*, 2011, **133**, 3312–3315; (*b*) H. F. T. Klare, Ph.D. Thesis, Westfälische Wilhelms-Universität Münster, Germany, 2011; (*c*) For an alternative preparation of (^{Si}*S*)-**4d**, see: P. Jankowski, E. Schaumann, J. Wicha, A. Zarecki, G. Adiwidjaja and M. Asztemborska, *Chem. Commun.*, 2000, 1029–1030.

[S3] M. Mewald, Ph.D. Thesis, Westfälische Wilhelms-Universität Münster, Germany, 2012.

[S4] C. Wang, G. Erker, G. Kehr, K. Wedeking and R. Fröhlich, *Organometallics*, 2005, **24**, 4760–4773.

[S5] G. M. Sheldrick, *SHELX-97: Program for Crystal Structure Refinement:* University of Göttingen, Göttingen, Germany, 1997.

[S6] J.-D. Chai and M. Head-Gordon, *Phys. Chem. Chem. Phys.*, 2008, **10**, 6615–6620.

[S7] L. Goerigk and S. Grimme, *Phys. Chem. Chem. Phys.*, 2011, **13**, 6670–6688.

[S8] T. H. Dunning, *J. Chem. Phys.*, 1989, **90**, 1007–1023.

[S9] M. J. Frisch, J. A. Pople and J. S. Binkley, *J. Chem. Phys.*, 1984, **80**, 3265–3269.

[S10] Gaussian 09, Revision B.01, M. J. Frisch, G. W. Trucks, H. B. Schlegel, G. E. Scuseria, M. A. Robb, J. R. Cheeseman, G. Scalmani, V. Barone, B. Mennucci, G. A. Petersson, H. Nakatsuji, M. Caricato, X. Li, H. P. Hratchian, A. F. Izmaylov, J. Bloino, G. Zheng, J. L. Sonnenberg, M. Hada, M. Ehara, K. Toyota, R. Fukuda, J. Hasegawa, M. Ishida, T. Nakajima, Y. Honda, O. Kitao, H. Nakai, T. Vreven, J. A., Jr. Montgomery, J. E. Peralta, F. Ogliaro, M. Bearpark, J. J. Heyd, E. Brothers, K. N. Kudin, V. N. Staroverov, R. Kobayashi, J. Normand, K. Raghavachari, A. Rendell, J. C. Burant, S. S. Iyengar, J. Tomasi, M. Cossi, N. Rega, N. J. Millam, M. Klene, J. E. Knox, J. B. Cross, V. Bakken, C. Adamo, J. Jaramillo, R. Gomperts, R. E. Stratmann, O. Yazyev, A. J. Austin, R. Cammi, C. Pomelli, J. W. Ochterski, R. L. Martin, K. Morokuma, V. G. Zakrzewski, G. A. Voth, P. Salvador, J. J. Dannenberg, S. Dapprich, A. D. Daniels, Ö. Farkas, J. B. Foresman, J. V. Ortiz, J. Cioslowski and D. J. Fox, Gaussian, Inc., Wallingford CT, 2009.

[S11] C. Y. Legault, *CYLview 1.0b*; Université de Sherbrook: Sherbrook, QC, Canada, 2009. http://www.cylview.org.

[S12] P. Bhattacharya, J. A. Krause and H. Guan, *Organometallics*, 2011, **30**, 4720–4729.

[S13] A. M. Tondreau, E. Lobkovsky and P. J. Chirik, *Org. Lett*., 2008, **10**, 2789–2792.

[S14] N. S. Shaikh, S. Enthaler, K. Junge and M. Beller, *Angew. Chem. Int., Ed*., 2008, **47**, 2497–2501.

[S15] H. Nishiyama and A. Furuta, *Chem. Commun.*, 2007, 760–762.

[S16] Z. Zuo, H. Sun, L. Wang and X. Li, *Dalton Trans*., 2014, **43**, 11716–11722.

[S17] A. J. Ruddy, C. M. Kelly, S. M. Crawford, C. A. Wheaton, O. L. Sydora, B. L. Small, M. Stradiotto and L. Turculet, *Organometallics*, 2013, **32**, 5581−5588.

[S18] S. E. Denmark and Y. Ueki, *Organometallics*, 2013, **32**, 6631−6634.

[S19] S.-F. Hsu and B. Plietker, *Chem. – Eur. J*., 2014, **20**, 4242–4245.

[S20] R. Lopes, J. M. S. Cardoso, L. Postigo and B. Royo, *Catal. Lett.*, 2013, **143**, 1061–1066.

[S21] T. Taniguchi and D. P. Curran, *Org. Lett*., 2012, **14**, 4540–4543.

[S22] J. Mohr, M. Durmaz, E. Irran and M. Oestreich, *Organometallics*, 2014, **33**, 1108–1111.

[S23] (*a*) T. T. Wenzel and R. G. Bergman, *J. Am. Soc. Chem.*, 1986, **108**, 4856–4867; (*b*) A. Kornath, F. Neumann and H. Oberhammer, *Inorg. Chem.*, 2003, **42**, 2894–2901.

[S24] T. T. Metsänen, P. Hrobárik, H. F. T. Klare, M. Kaupp and M. Oestreich, *J. Am. Chem. Soc.*, 2014, **136**, 6912–6915.
